# Cord blood–derived V_δ_2^+^ and V_δ_2^−^ T cells acquire differential cell state compositions upon in vitro expansion

**DOI:** 10.1126/sciadv.adf3120

**Published:** 2023-06-16

**Authors:** Jeremy Wee Kiat Ng, Kar Wai Tan, Dian Yan Guo, Joey Jia Hui Lai, Xiubo Fan, Zhiyong Poon, Tse Hui Lim, Alvin Soon Tiong Lim, Tony Kiat Hon Lim, William Ying Khee Hwang, Shang Li, Connie J. Eaves, Yeow Tee Goh, Alice Man Sze Cheung

**Affiliations:** ^1^ Department of Molecular Pathology, Translational Pathology Centre, Singapore General Hospital, Singapore, Singapore.; ^2^ Department of Clinical Translational Research, Singapore General Hospital, Singapore, Singapore.; ^3^ Tessa Therapeutics Ltd, Singapore, Singapore.; ^4^ Department of Haematology, Singapore General Hospital, Singapore, Singapore.; ^5^ Critical Analytics for Manufacturing Personalized-Medicine, Singapore-MIT Alliance for Research and Technology, Singapore, Singapore.; ^6^ Department of Molecular Pathology, Cytogenetics Laboratory, Singapore General Hospital, Singapore, Singapore.; ^7^ National Cancer Centre Singapore, Singapore, Singapore.; ^8^ Cancer and Stem Cell Biology, Duke-NUS Medical School, Singapore, Singapore.; ^9^ Terry Fox Laboratory, BC Cancer Agency, Vancouver, Canada.

## Abstract

Human cord blood–derived γδ T cells (CB_γδ_) display a highly diverse TCR_γδ_ repertoire and have a unique subtype composition different from fetal or adult peripheral blood counterparts. We expanded CB_γδ_ in vitro using an irradiated Epstein-Barr virus–transformed feeder cell–based modified rapid expansion protocol (REP). Single-cell RNA sequencing tracked progressive differentiation of naïve CB_γδ_ into cells expressing neoantigen-reactive tumor-infiltrating lymphocyte as well as tissue-resident memory precursor–like and antigen-presenting cell–like gene signatures. TCR_γδ_ clonal tracing revealed a bias toward cytotoxic effector differentiation in a much larger proportion of V_δ_2^−^ clones compared to V_δ_2^+^ clones, resulting in the former being more cytotoxic at the population level. These clonotype-specific differentiation dynamics were not restricted to REP and were recapitulated upon secondary nonviral antigen stimulations. Thus, our data showed intrinsic cellular differences between major subtypes of human γδ T cells already in operation at early postnatal stage and highlighted key areas of consideration in optimizing cell manufacturing processes.

## Teaser

Cord blood derived Vδ2^-^&nbsp;cells preferentially adopt CTL bias differentiation program in vitro.

## INTRODUCTION

γδ T cells represent a special class of unconventional T cells defined by their expression of the somatically rearranged T cell receptor (TCR) γ and δ chains. These cells have been shown in preclinical models to elicit potent cytotoxicity against various types of cancer cells including chemotherapy-resistant leukemic cells (*1*). Early reports (*2*, *3*) suggesting that reconstitution of donor-derived γδ T cells improved disease-free survival in patients with leukemia receiving αβ T cell–depleted allogeneic bone marrow (BM) transplants have resulted in burgeoning of interest in the antitumor effect of these cells. Tumor-infiltrating γδ T cells (γδTIL) have also been identified in various types of cancers and positively associated with prognosis (*4*–*6*). In some cases, the isolated γδTIL had also demonstrated cytotoxic activity against autologous tumor cells ex vivo (*7*, *8*). Notably, an in silico study of 18,000 patients with cancer established that a tumor-associated γδ T cell gene signature (GS) correlated significantly with improved overall survival across 39 different malignancies (*4*), further advocating the application of γδ T cells in cancer therapy.

The choice of γδ T cell source and subsets directs subsequent manipulations needed for clinical scale manufacturing and is an important consideration for maximizing the antitumor properties of γδ T cells in adoptive cellular immunotherapy (ACT). Most initial efforts have focused on the adult peripheral blood (PB)–derived semi-invariant V_γ_9V_δ_2 T cell subset activated indirectly by phosphoantigens (P-Ag) via butyrophilin 2A1 and butyrophilin 3A1 (*9*–*12*). However, the objective response rates from these early clinical trials were generally low and demonstrated suboptimal treatment efficacies (*13*). This is partly contributed by the functional heterogeneity observed in V_γ_9V_δ_2 cells in healthy donors (*14*, *15*). At the same time, the identification of the mucosal-associated invariant T (MAIT) cell–like V_γ_9V_δ_2 population in human cord blood (CB), displaying differential phenotypic and functional profiles from PB-derived V_γ_9V_δ_2 (*15*), calls for better characterization of CB-derived γδ T cells (CB_γδ_). CB is enriched with the more clonally diverse V_δ_2^−^ (particularly V_δ_1^+^) cells (*16*–*19*). Preclinical studies of adoptively transferred healthy donor–derived V_δ_1^+^ cells documented a promising cytotoxic activity against a variety of solid tumors (*20*–*22*) and leukemias (*22*–*24*), strengthening the case for their use in ACT. The low frequencies of these cells in adult circulation and their apparent insensitivity to P-Ag stimulation had hampered the clinical development of V_δ_2^−^ cells for therapeutic purposes. Nevertheless, improved characterization of these cells in a series of seminal studies (*16*, *25*, *26*) has led to the description of various in vitro expansion protocols to expand these cells from adult PB and even tissue samples (*22*, *27*).

Despite progress in large-scale γδ T cell expansion, there is little understanding of the effect of culture-induced differentiation and a paucity in the characterization of in vitro–generated γδ T cells. While it is recognized that stimulatory expansions drive effector maturation, the extent to which changes in transcriptional cell states are being induced in different γδ T cell subtypes remains unclear. To fill this gap, we focused on the mostly naïve, clonally diverse CB_γδ_ and performed single-cell characterization to track their in vitro activation and differentiation into mature effectors.

## RESULTS

### Expansion of leukemia targeting CB_γδ_ in vitro

We first characterized the γδ T cell populations in our local collection of CB and compared to those found in adult PB. Consistent with previous reports (*28*, *29*), majority of the γδ T cells in PB (60.55 ± 6.99%, *n* = 11) were of V_δ_2^+^ subtype that was present at a low level in CB (4.25 ± 0.92%, *n* = 19; fig. S1A). Conversely, more than half of the total CB_γδ_ were of the V_δ_1^+^ subtype (52.75 ± 2.65%, *n* = 19) and the remaining (38.81 ± 3.05%, *n* = 19) expressing neither the V_δ_1 nor V_δ_2 chain (fig. S1A). Consistent with the neonatal origin of CB, CB_γδ_ displayed a predominately central memory (T_CM_; CD45RA^−^CD27^+^) and naïve (T_N_; CD45RA^+^CD27^+^) phenotype regardless of their subtypes (*n* = 19; fig. S1B and table S1). In contrast, the bulk of PB_γδ_ showed CD27^−^ effector phenotype (T_E_) (*n* = 11; fig. S1B and table S1). Together, our data showed that CB presents more enriched and less differentiated source of V_δ_2^−^ γδ T cells compared to adult PB.

**Fig. 1.**
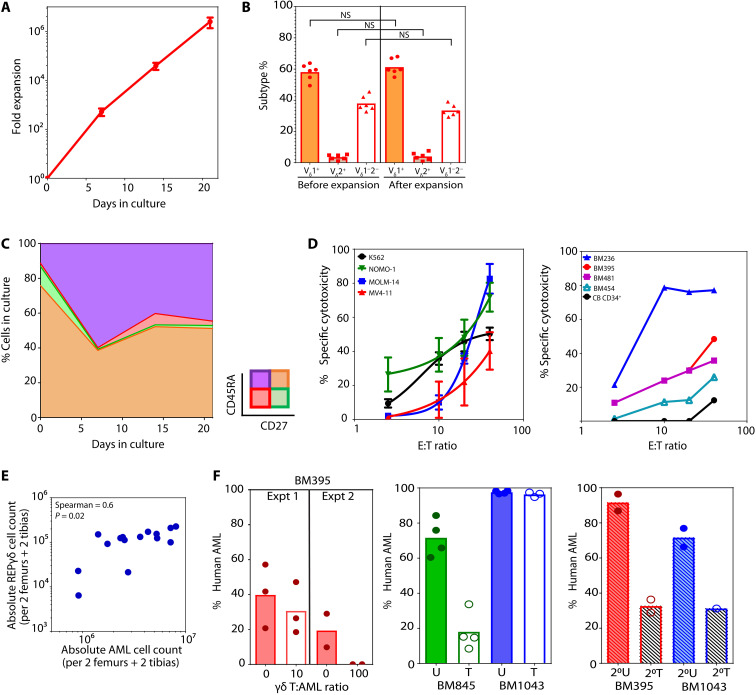
Expansion of leukemia targeting CB_γδ_. (**A**) Average (± SEM) fold expansion of CB_γδ_ over 21 days in REP cultures (*n* = 6). (**B**) CB_γδ_ subtype composition before and after in vitro expansion. NS, not significant. (**C**) Average proportion of CB_γδ_ with differential cell surface expression of CD45RA and CD27 over 21 days of REP cultures (*n* = 6). (**D**) Chromium release assay of AML cell lines (left) and primary AML patient samples or allogeneic CD34^+^ CB cells (right) at the indicated effector-to-target (E:T) cell ratio. Graph shows mean ± SEM of three or four independent experiments for each cell line. (**E**) Calculated absolute number of human AML and γδ T cells in BM of REP_γδ_ infused AML-PDX (*n* = 15). (**F**) Human leukemia burden in AML-PDX mice BM with (T) or without treatment (U) of D14-REP_γδ_. Each point represents data from individual mice, and bars show the average value for each group.

We expanded the different subtypes of γδ T cells in vitro by a modified rapid expansion protocol (REP) (*30*, *31*) using feeder cells composed of irradiated PB mononuclear cells (PBMCs) and Epstein-Barr virus–lymphoblastoid cell line (EBV-LCL). An average of 2.5 ± 1.2 × 10^6^-fold expansion of the starting CB_γδ_ was achieved in 3 weeks (*n* = 6; Fig. 1A and Table 1). In contrast, parallel REP cultures of CB_γδ_ and PB_γδ_ for 14 days (D14) showed minimal expansion of PB_γδ_ (4.7 ± 1.4 × 10^3^ and 7.1 ± 6.3-fold expansion in CB_γδ_ and PB_γδ_, respectively; *P* = 0.01, *n* = 3 each; fig. S1C and table S2). The lack of expansion in PB_γδ_ was not due to the failure to activate PB_γδ_ as a similar, if not greater, increase in activation markers CD69, CD25 [interleukin-2 receptor α (IL-2Rα)], and CD107a [lysosomal associated membrane protein 1 (LAMP-1)] was recorded in PB_γδ_ compared to CB_γδ_ upon REP culture (*n* = 3 each; fig. S1, D and E, and table S3). It was also not due to preferential expansion of V_δ_2^−^ cells because the proportion of V_δ_1^+^, V_δ_2^+^, and V_δ_1^−^_δ_2^−^ T cells was maintained before and after expansion (Fig. 1B, fig. S1F, Table 2, and table S4). Time-course analysis of CB_γδ_ in culture (REP_γδ_) revealed extensive differentiation into CD27^−^ effector phenotype regardless of the V_δ_ subtype (Fig. 1C; fig. S1, G and H; and Table 3), suggesting that the poor PB_γδ_ expansion was likely the result of exhaustive differentiation upon REP culture. Together, our data showed that CB_γδ_ is superior to PB_γδ_ for REP expansion.

**Table 1.** Calculated absolute number of γδ T cells (×10^6^) in REP culture.

	CB12	CB14	CB26649	CB39382	CB39677	CB40933
**Day 0**	0.05	0.165	0.024	0.021	0.038	0.037
**Day 7**	7.7	4.77	25.558	15.055	36.495	9.296
**Day 14**	969.408	518.056	1302.444	1219.504	3227.85	624.067
**Day 21**	49500	57820	71703.429	46666.667	301968	21634.309

**Table 2.** Percentage of V_δ_1^+^, V_δ_2^+^, and V_δ_1^−^2^−^ cells within total γδ T cell population over 21 days in REP culture.

V_δ_1^+^%	CB12	CB14	CB26649	CB39382	CB39677	CB40933
**Day 0**	49.4	59.6	61.9	58.9	63.8	54.7
**Day 7**	61.2	54.8	69.3	63.4	66.3	63.6
**Day 14**	64.2	55.9	65.2	54.6	63.2	61.2
**Day 21**	67	59.5	68	58.6	59.7	54.8
**V_δ_2^+^%**	**CB12**	**CB14**	**CB26649**	**CB39382**	**CB39677**	**CB40933**
**Day 0**	4.7	4.8	1	5.3	2.7	2.7
**Day 7**	5.7	7.1	0.7	3.2	2.5	1.9
**Day 14**	3.51	7.08	1.2	6.5	3.5	4.6
**Day 21**	2.1	3.18	0.8	7.6	5.8	4.5
**V_δ_1^−^2^−^%**	**CB12**	**CB14**	**CB26649**	**CB39382**	**CB39677**	**CB40933**
**Day 0**	45.5	35.6	36.8	35	32.7	42.1
**Day 7**	27.5	34.3	29.8	33.2	30.9	34.5
**Day 14**	31.5	35.9	33.4	38.6	32.3	32.6
**Day 21**	29.3	36.3	31	32.8	32.3	39.1

**Table 3.** Percentage of T_N_ (CD45RA^+^CD27^+^), T_CM_ (CD45RA^−^CD27^+^), and T_E_ (CD45RA^+/−^CD27^−^) within γδ T cell population over 21 days in REP culture.

Sample	Days in culture	% Within total γδ T			% Within V_δ_1^+^			% Within V_δ_2^+^			% Within V_δ_1^−^2^−^		
		T_N_	T_CM_	T_EM_	T_N_	T_CM_	T_EM_	T_N_	T_CM_	T_EM_	T_N_	T_CM_	T_EM_
**CB12**	**0**	73.2	1.7	25.2	77.6	0.9	21.6	90.9	0	9.1	66.4	2.8	30.8
	**7**	41.1	0.2	58.6	39.1	0.3	60.6	73.3	0	26.7	27.6	0	72.4
	**14**	60.4	0.4	39.2	64.1	0.3	35.7	59.8	1.2	39	52	0.6	47.5
	**21**	44.6	1.7	53.7	46.8	1.7	51.4	57.6	3	39.4	35.9	1.4	62.8
**CB14**	**0**	98.1	1.9	0	96.7	3.3	0	100	0	0	100	0	0
	**7**	36.5	0.4	63.1	40.2	0.7	59.1	48.5	0	51.5	21.5	0	78.5
	**14**	64.3	0.1	35.6	67.4	0	32.5	63.3	0.5	36.2	59	0.1	40.9
	**21**	42.2	0.7	57.2	44.6	0.6	54.8	50.2	2.3	47.5	36.3	0.5	63.2
**CB26649**	**0**	51.4	1.8	46.7	55.6	2.2	42.2	62.5	0	37.5	43.5	1.3	55.2
	**7**	20.9	0	79.0	21.8	0	78.2	39.8	0	60.2	18.1	0	81.9
	**14**	28.1	0.1	71.7	30.5	0.1	69.4	30.7	0	69.4	23.2	0.1	76.7
	**21**	69.6	0.2	30.3	72	0.2	27.8	66.1	0.9	33.1	64.2	0.2	35.6
**CB39382**	**0**	83.7	15.1	1.2	93.2	6.8	0	93.5	3.2	3.2	92.5	6.1	1.4
	**7**	45.3	0	54.7	32.1	0	67.9	29.9	0.2	69.8	27.4	0	72.6
	**14**	28.2	0.4	71.4	32.7	0.4	66.9	29	1.5	69.5	23.1	0.2	76.7
	**21**	57.8	0.9	41.3	62.5	1	36.6	51.6	0.8	47.7	49.8	0.8	49.4
**CB39677**	**0**	72.8	23.9	3.3	70.7	26	3.3	73.7	18.4	7.9	76.9	20.1	3
	**7**	56.6	2.2	41.3	60.2	2.4	37.5	64.4	4.2	31.5	48	1.6	50.4
	**14**	62.1	4.2	33.7	65.9	3.7	30.4	62.3	7.5	30.3	54	4.7	41.3
	**21**	62.9	4.6	32.5	67.2	4.3	28.6	60.6	5.8	33.6	53.8	5	41.1
**CB40933**	**0**	75.8	21.8	2.4	74.9	23.6	1.5	68.7	27.7	3.6	77.4	19.1	3.5
	**7**	31.2	1.2	67.6	34	1.3	64.7	28.8	1.5	69.7	26.2	0.9	72.9
	**14**	70.1	1.4	28.4	73.9	1.4	24.7	59	1.8	39.2	63.5	1.5	35
	**21**	30.1	2	67.8	34.1	2.2	63.7	27.2	0.8	72	23.6	2	74.4

To test whether REP_γδ_ displayed cross-reactivities against cancer cells, we coincubated the expanded cells with a panel of acute myeloid leukemia (AML) cell lines and observed dose-dependent cytotoxicity against all the tested cell lines (Fig. 1D, left). Similar results were also obtained when REP_γδ_ was tested against four different primary AML patient samples (Fig. 1D, right). Cytotoxicity against two solid cancer cell lines, Hep3B and HCT116, were also observed (fig. S1I), confirming that REP_γδ_ were reactive against various cancer cells in vitro. We next turned to establish the utility of REP_γδ_ in targeting leukemia in vivo. We first evaluated the ability of these cells to home to the mouse BM, spleen (SPN), and liver (LV). Nonobese diabetic–severe combined immunodeficient IL2Rg^null^ (NSG) mice xenografted with primary AML patient sample (AML-PDX mice) were intravenously infused with REP_γδ_ (fig. S1J, left). REP_γδ_ could be readily detected in mouse BM, SPN, and LV as well as circulation on days 1 and 5 after infusion (fig. S1J, right, and table S5), confirming the homing and maintenance of REP_γδ_ in mouse hematopoietic tissues. In addition, the level of REP_γδ_ significantly correlated with the amount of AML cells present in the BM [Spearman correlation coefficient (*r*) = 0.6, *P* = 0.02; Fig. 1E and table S6) and SPN (Spearman *r* = 0.64, *P* = 0.01; fig. S1K and table S6). Together, these data suggest chemotaxis of REP_γδ_ toward human AML and their subsequent in vivo expansion following leukemic cell encounter at the hematopoietic tissues. Fluorescence-activated cell sorting (FACS) analysis showed that cell surface expression of both CXCR3 and CXCR6 in REP_γδ_ was significantly increased following culture (*P* < 0.05; fig. S1L and Table 4), suggesting that CXCR3- and CXCR6-related signaling axis may play a role in directing the in vivo trafficking of REP_γδ_.

**Table 4.** Mean fluorescence intensity (MFI) of CXCR3, CXCR6, and CCR5 cell surface staining in γδ T cells before and after REP culture.

	MFI_stain_ − MFI_isotype_					
	CXCR3		CXCR6		CCR5	
**Sample**	**Day 0**	**Day 21**	**Day 0**	**Day 21**	**Day 0**	**Day 21**
**CB1**	58.2	436.3	0.2	576	49.9	24.2
**CB9**	44	578.2	4.6	828.1	81.5	82.1
**CB8**	50.2	431	0	422	29.8	32.3
**D0 versus D21 *P* value**	0.01		0.04		0.49	

Because infused REP_γδ_ were able to home to mouse hematopoietic tissues that were grafted with human AML cells, we next sought to determine whether the infused REP_γδ_ could also suppress leukemia in vivo. Co-infusion of REP_γδ_ together with primary human AML cells into NSG mice led to dose-dependent leukemia suppression, with full abrogation of AML repopulation to undetectable level when cells were co-infused at effector-to-target (E:T) ratio of 100 (Fig. 1F, left, and fig. S1M). In established AML-PDX mouse models, REP_γδ_ infusion resulted in sustained decrease of leukemia burden at 1 month after REP_γδ_ infusion in one of the two primary AML samples tested (BM845; Fig. 1F, middle, and fig. S1N). Intriguingly, leukemic cells harvested from mice nonresponsive to REP_γδ_ treatment (BM1043 and BM395 at E:T ratio of 10) displayed reduced regeneration ability when transplanted into secondary recipients as compared to untreated BM (Fig. 1F, right, and fig. S1O). In addition, for BM1043 that harbors a *t*(9;11) translocation, we were able to detect *t*(9;11) negative human B cells in secondary recipient (fig. S1P), suggestive of the re-emergence of nonleukemic human hematopoiesis. Our results suggest that the infused REP_γδ_ likely targeted leukemic cells in vivo, causing an increased leukemic cell turnover and resulted in decreased leukemia repopulating potential. In contrast, minimal cytotoxicity of REP_γδ_ against normal hematopoietic cells from allogeneic CB samples in vitro was observed (Fig. 1D, right, black line) and infusion of up to 5 × 10^8^ REP_γδ_/kg into NSG mice xenografted with allogeneic CB cells resulted in no significant graft-versus-host disease (GvHD) symptoms (fig. S1Q). Thus, our data confirmed that a subset of REP_γδ_ was able to cross-react and specifically target leukemic cells.

### Innate- and adaptive-like expansion characteristics of CB_γδ_ in culture

**Fig. 2.**
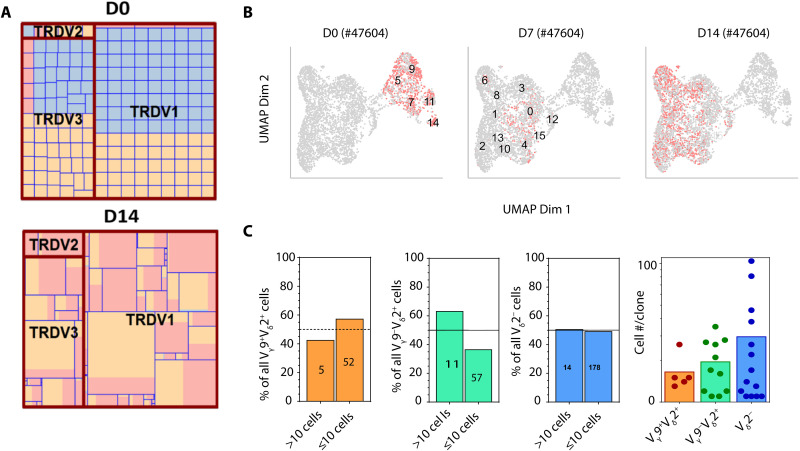
Expansion characteristics of CB_γδ_ in REP cultures. (**A**) Relative size of single–γδ T cell clone from two CB samples (CB40145 and CB40259) at D0 and D14 of REP culture represented by each individual square. Beige, light blue, and pink fill represents phenotypically defined T_N_ (CD27^+^CD45RA^+^), T_CM_ (CD27^+^CD45RA^−^), and T_E_ (CD27^−^) cells, respectively. (**B**) Uniform Manifold Approximation and Projection (UMAP) of single-cell RNA sequencing (scRNA-seq) of nonmanipulated (D0) and D7 and D14 REP_γδ_ cultured from a single CB sample (CB47604). (**C**) Cellular representation of large (>10 cells) versus small (≤10 cells) clones among V_γ_9^+^V_δ_2^+^ (left), V_γ_9^−^V_δ_2^+^ (middle left), and V_δ_2^−^ (middle right) cells. Number within bar indicates the number of clones in each group. Right: Average clone size of the indicated clonotype. Each point represents a single clone, and bars show the average value for each group.

While conventional polymerase chain reaction (PCR)–based spectratyping showed that REP_γδ_ were polyclonal (fig. S2A), the extent of heterogeneity in expansion potential in REP culture among different CB_γδ_ clones could not be determined. To evaluate the extent of CB_γδ_ clonal focusing in REP cultures, we set up 382 single–γδ T cell cultures from four CB samples. All the cells had proliferated (albeit at different rates) by day 14 of expansion to give rise to a wide range of clone sizes. Nevertheless, only a small subset of REP culture–stimulated V_δ_1^+^ and V_δ_1^−^_δ_2^−^ CB_γδ_ cells displayed strong adaptive clonal expansion characteristics, with only 19.4% (74 of 382) of the clones generating >500 cells (Fig. 2A and table S7). The low frequency of V_δ_2^+^ cells in our single-cell cultures precluded accurate evaluation of this subset. To further characterize REP_γδ_ throughout expansion, we performed single-cell immune profiling (scTCR-seq) and single-cell RNA sequencing (scRNA-seq) in FACS-purified γδ T cells from a CB sample (#47604) before (D0), at midpoint (D7), and at the end of the D14 of REP expansion. scRNA-seq and scTCR-seq from two other cords (#41357 and #41365) were also obtained at D14 of REP culture. Combining the scRNA-seq and scTCR-seq from all three cords following quality filtering to remove poor quality cells and unproductive cells, a total of 4574 cells with paired scRNA-seq/scTCR-seq were analyzed. We identified 16 cell clusters following dimension reduction and unsupervised clustering, with the D0 nonmanipulated CB_γδ_ clearly segregated from cultured REP_γδ_ (Fig. 2B). Gini coefficient confirmed some extent of clonal focusing and correlated well with the frequency of >10 cells clones observed in each cluster following expansion (fig. S2B). Consistently, the top 30 largest clones (ranging 11 to 104 cells per clone) among the 317 clones detected in D14 REP_γδ_ made up approximately half (52.7%) of total cells (fig. S2C), confirming an adaptive immune cell response. Despite this, we noted an increased extent of “innate-like,” non–clonotype-specific expansion specifically among V_γ_9^+^V_δ_2^+^ cells. The semi-invariant V_γ_9^+^V_δ_2^+^ clonotype comprised many small clones (≤10 cells), which collectively formed the bulk (57.4%) of the V_γ_9^+^V_δ_2^+^ population among D14 REP_γδ_ (Fig. 2C, left). This contrasted with both V_γ_9^−^V_δ_2^+^ and V_δ_2^−^ cells with a lower representation of small clones (Fig. 2C, middle panels). Likewise, the average number of cells among clones with >10 cells was smaller in V_γ_9^+^V_δ_2^+^ clones when compared against other clonotypes (Fig. 2C, right). Thus, similar to earlier reports on PB_γδ_ (*32*, *33*), we observed the adoption of adaptive- and innate-like expansion modes by different subsets of CB_γδ_ in REP culture.

### Enrichment of activation-primed innate-like cell state among V_γ_9^+^V_δ_2^+^ CB_γδ_

**Fig. 3.**
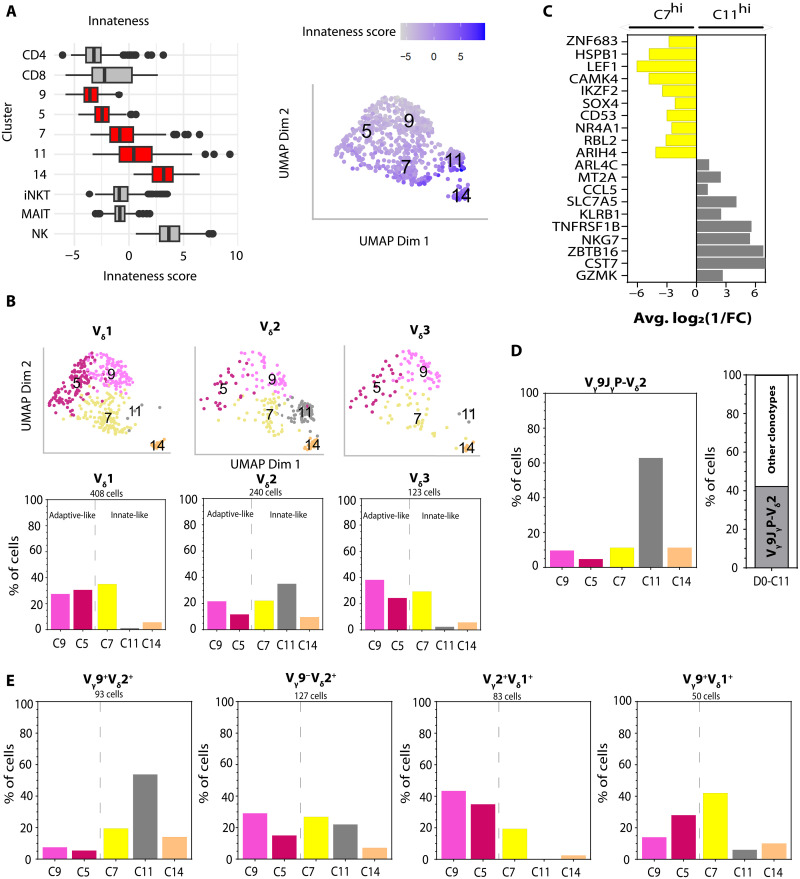
Variable innate- and adaptive-like cell transcriptome profiles among CB_γδ_. (**A**) Innateness score of nonmanipulated (D0) CB_γδ_ cell clusters (red boxes). Left: Line within box indicates the median, with box limits extend from the 25th to 75th percentiles of all cells. Whiskers capture scores within 1.5× of the interquantile range. Cells with scores larger or smaller than the range are plotted individually. For comparison, the calculated innateness score of prototypical adaptive cells (CD4 and CD8) and innate cells (NK cells) are also plotted in gray. The reference dataset was obtained from previously published work (*34*). Right: Innateness score of each individual cell shown on UMAP plot. iNKT, invariant natural killer T. (**B**) Distribution of V_δ_1/2/3 cells across nonmanipulated D0 CB_γδ_ cell clusters as visualized on UMAP (top) or summarized in bar chart (bottom). (**C**) Selected list of differentially expressed genes (DEGs) between C7 and C11. Fold change (FC) represents average gene expression in C7 relative to that in C11. (**D**) Left: Distribution of V_γ_9J_γ_P-V_δ_2^+^ cells across all D0 clusters. Right: Clonal representation of V_γ_9J_γ_P-V_δ_2^+^ cells within D0-C11. (**E**) Distribution of V_γ_9^+^V_δ_2^+^, V_γ_9^−^V_δ_2^+^, V_γ_2^+^V_δ_1^+^, and V_γ_9^+^V_δ_1 cells across D0 cell clusters. The total number of single cells within each clonotype is indicated.

Given our observations of differential CB_γδ_ cell expansion modalities in REP culture, we asked whether such characteristics were already reflected in preexisting cell states before in vitro stimulation. Examination of D0-CB_γδ_ found that these cells displayed an “innateness gradient,” with median scores of C7, C11, and C14 being similar to invariant natural killer (NK) T, MAIT, and NK cells while that of C5 and C9 were more similar to CD4/8 cells (Fig. 3A and Table 5). The innateness-associated GS (particularly MAIT-like profile) (*34*) was more enriched in C7, C11, and C14, as opposed to C5 and C9 displaying higher resemblance to an adaptive, naïve CD4-like signature (Fig. 3A and fig. S3A). We excluded C14 cells in further characterizations due to the small size of this population (51 cells, comprising only 8% of total cells in day 0). Consistent with the innate characteristic of being a rapid effector (*34*), we found an enrichment of cells expressing granzyme K (*GZMK*) or interferon-γ (*IFN*-γ) in C7/C11 compared to C5/C9 (*GZMK*: 43.7 versus 4.8%; *IFN*-γ: 17.9 versus 0.5%; *P* < 0.01; fig. S3C and table S8). Conversely, significantly higher percentage of cells in C5/C9 expressed the prototypical naïve/central memory T cell marker CCR7 compared to C7/C11 (22.8 versus 8.9%, *P* < 0.01; fig. S3C and table S8).

**Table 5.** Summary statistics of innateness scores in D0 cells. IQR, interquartile range; iNKT, invariant NKT cells; MAIT, mucosal-associated invariant T cells.

	Quartile			
Cell type	Q1	Median	Q3	IQR
**CD4**	−3.94	−3.21	−2.58	1.36
**CD8**	−3.37	−2.24	0.27	3.64
**C9**	−4.38	−3.82	−3.08	1.3
**C5**	−3.29	−2.6	−1.9	1.39
**C7**	−1.83	−0.88	0.43	2.26
**C11**	−1.03	0.36	2.02	3.05
**C14**	2.25	3.18	4.14	1.89
**iNKT**	−1.37	−0.89	−0.12	1.25
**MAIT**	−1.26	−0.84	−0.4	0.86
**NK**	2.82	3.65	4.62	1.8

Mapping the distribution of the D0-V_δ_1/2/3 subsets across the transcriptionally defined cell clusters revealed that all three subsets comprised both “adaptive-like” and innate-like cells (Fig. 3B and table S9). Notably, we found that C11 was almost exclusively V_δ_2^+^ (Fig. 3B). Thus, there were two distinct clusters of V_δ_2^+^ innate-like cells (C7 and C11) as compared to V_δ_1^+^ and V_δ_3^+^ innate-like cells being concentrated only in C7. Differential gene expression analysis between C7 and C11 showed that the former displayed higher expression of T cell regulatory genes such as lymphoid enhancer binding factor 1 (*LEF1*), IKAROS family zinc finger (*IKZF2*), and SRY-box transcription factor 4 (*SOX4*), while C11 had enhanced expression of inflammatory/cytotoxic genes, including cystatin F (*CST7*), natural killer cell granule protein 7 (*NKG7*), granzyme K (*GZMK*), and C-C motif chemokine ligand 5 (*CCL5*) (Fig. 3C and table S10), indicating an activation-primed cell state. Majority (>50%) of the predominantly innate-like V_γ_9^+^V_δ_2^+^ cells were found within C11 (Fig. 3E and table S11) and were associated with the use of V_γ_9-J_γ_P chain. More than 60% of the public V_γ_9^+^J_γ_PV_δ_2^+^ clonotype known to have strong P-Ags reactivity (*35*, *36*) was found within C11, contributing to >40% of all D0-C11 cells (Fig. 3D). This contrasts with the V_γ_9^+^J_γ_1V_δ_2^+^ clonotype that was enriched in C7 (fig. S3D and table S11). On the other hand, V_γ_9^−^V_δ_2^+^ and other V_δ_2^−^ clonotypes were more heterogeneously spread out along this adaptive-innate axis (Fig. 3E and table S11). Thus, our transcriptomic data confirmed variable extent of preexisting innate- and adaptive-like cell states among different CB_γδ_ clonotypes. In particular, the enrichment of activation-primed cell state (C11) among V_γ_9^+^V_δ_2^+^ is consistent with their increased extent of an innate-like expansion mode observed in our REP cultures (Fig. 2C).

### Generation of CTL, T_RM_ precursor–like, and APC-like γδ T cells in REP culture

**Fig. 4.**
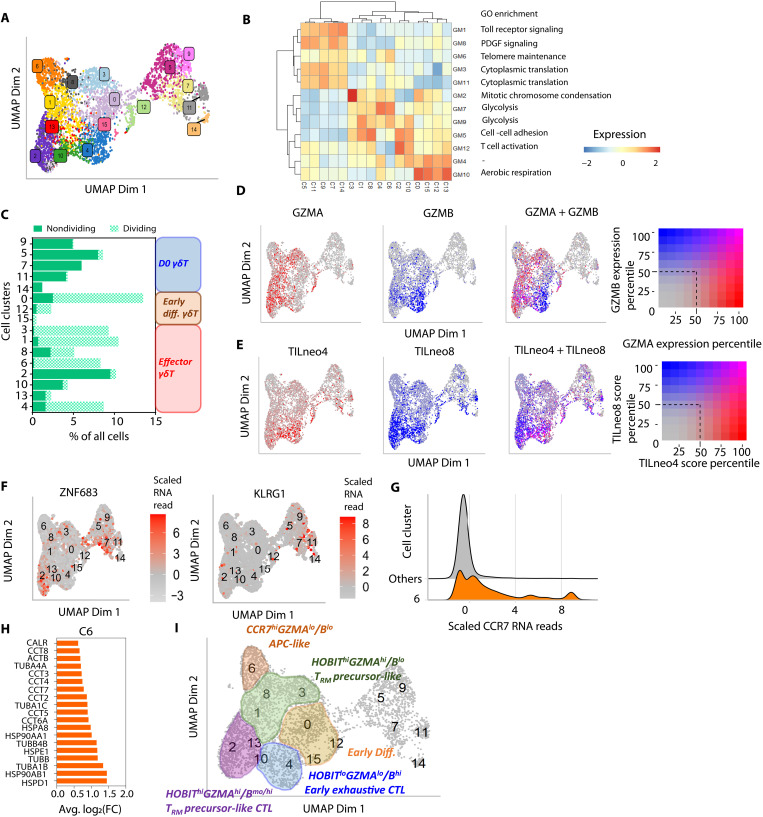
Differentiation of CB_γδ_ into CTL, T_RM_ precursor–like, and APC-like cells in REP culture. (**A**) UMAP of integrated scRNA-seq profiles of nonmanipulated (D0) CB_γδ_ and in vitro–expanded REP_γδ_. (**B**) Twelve distinct gene modules (GMs) identified via unsupervised clustering of marker genes expressions across all clusters. Heatmap indicates the aggregated gene expression score per GM in each cluster. Gene Ontology (GO) enrichment analysis was used to identify significantly enriched processes for each GM. (**C**) Cumulative percentage of cells in G_1_ (nondividing) and S/G_2_-M (dividing) phase of cell cycle within each cell cluster. (**D** and **E**) Relative *GZMA*/*GZMB* RNA expression (D) and neoantigen-reactive tumor-infiltrating CD4/8^+^ (TILneo4/8) GS scores (E) across all cell clusters. (**F**) Scaled RNA read counts of *ZNF683 *and *KLRG1* in individual cells across all cell clusters. (**G**) Ridge plot of scaled RNA read counts of *CCR7* showing increased level in C6 compared to all remaining REP_γδ_ cell clusters. (**H**) Selected list of marker genes of C6 cells. (**I**) D14 cell cluster groupings and annotations identified in our dataset.

To compare and characterize transcriptional changes among in vitro–generated REP_γδ_, we analyzed the single-cell transcriptomic profiles of all samples and time points together (Fig. 4A, fig. S4A, and table S9). Compared against the published single-cell transcriptome data (*19*), majority (56.4%) of our REP_γδ_ were not mapped to any of the described cell states in nonmanipulated γδ T cells regardless of developmental origin (fig. S4B, dark blue bars). None of the REP_γδ_ resembled the exhausted cytotoxic T lymphocyte (CTL) PB_γδ_ phenotype described in the same study (fig. S4B). Instead, we found REP_γδ_ expressing high surface protein level of Fas (CD95) and CD62L and low cell surface expression of CD69, resembling the stem-like T cell (T_SCM_) phenotype (fig. S4C and table S12) (*37*, *38*). Differentially expressed gene (DEG) analysis showed that T cell activation and differentiation GSs were heterogeneously spread across REP_γδ_, and overlapping marker gene sets were observed across the different cell clusters (fig. S4D and table S13). Unsupervised clustering of these DEGs resulted in 12 gene coexpression modules (GM) enriched with genes defining distinct cellular biological processes (Fig. 4B and tables S14 and S15). Enrichment of the aerobic respiration GM (GM10) in C0/12/13/15 was consistent with the strong induction of mitochondrial genes found in these cells (Fig. 4B and fig. S4E), presumably to cope with increased metabolic requirements upon activation in culture. Notably, a switch toward a glycolysis-prominent metabolic gene program (GM7/9) was seen in majority of the remaining REP_γδ_ clusters (Fig. 4B and fig. S4E) and is consistent with the documented metabolic rewiring during effector T cell differentiation (*39*). Thus, a projected transition from early differentiating cells (C0/12/15) to differentiated effectors (C1/2/3/4/6/8/10) was observed in our REP culture. Cell proliferation status was concordant with the metabolic changes and proposed differentiation stages, with D0 cells being mostly quiescent while D7 and D14 REP_γδ_ were predominantly actively proliferating except C2/10/13 (Fig. 4C and fig. S4F). The loss in proliferative capacity in C2/10/13 was accompanied by the up-regulation of cytotoxic T cell genes [IL-32, cathepsin W (*CTSW*), and granzyme B (*GZMB*); fig. S4D].

Intriguingly, we found that expression of *GZMA* and *GZMB* did not correlate with each other. Instead, differentiated effectors displayed highly variable ratio of both genes (Fig. 4D and fig. S4G). C4 was *GZMA*^lo^/*B*^hi^ in contrast to C1/3/8 being *GZMA*^hi^/*B*^lo^ that resembled the cytotoxic enzyme profile of long-persisting CD4^+^ CAR-T cells in patients with chronic lymphocytic leukemia (CLL) (*40*). Likewise, C6 showed low expression of both genes (*GZMA*^lo^/*B*^lo^), while C2/10/13 were *GZMA*^hi^ with moderate to high average expression of *GZMB* (*GZMB*^mo/hi^; Fig. 4D). We found that only *GZMB*^mo/hi^ cell clusters (C2/10/13/4) showed high enrichment of the reported neoantigen-reactive tumor-infiltrating CD4^+^ and CD8^+^ (TILneo4 and TILneo8) GSs (Fig. 4E) (*41*), suggestive of these cells being tumor-reactive CTL. In addition, C4 (*GZMA*^lo^/*B*^hi^) cells also showed enrichment of the exhaustive T cell GS (fig. S4H) (*42*), indicative of an early dysfunctional phenotype. This was further corroborated by the highly proliferative nature of the cells in the cluster (Fig. 4C), similar to early dysfunctional intratumoral CD8 T cells (*43*).

Other than cytotoxic effector genes, our analysis showed that C1/3/8/2/10 also up-regulated the “cell-cell adhesion” GM (GM5; Fig. 4B), suggestive of enhanced potential for tissue trafficking. In line with this, only C1/3/8/2/10 displayed tissue-resident memory T (T_RM_) precursor-like phenotype (*44*, *45*) of high *ZNF683* (HOBIT) expression together with the absence of *KLRG1* (Fig. 4F). Conversely, the secondary lymphoid organ-homing receptor *CCR7* was found to be specifically enriched in C6 cells among REP_γδ_ (Fig. 4G). In addition, a range of genes involved in antigen processing and presentation including heat shock and chaperone genes were also found to be up-regulated in C6 (Fig. 4H and table S13), resembling classic antigen-presenting cells (APCs). Together, our data highlighted the progressive differentiation of CB_γδ_ into four major cell states upon stimulation in REP culture: T_RM_ precursor–like, APC-like, T_RM_ precursor–like CTL, and early exhaustive CTL (Fig. 4I).

### Enrichment of CTL bias clones among in vitro–differentiated V_δ_2^−^ cells

**Fig. 5.**
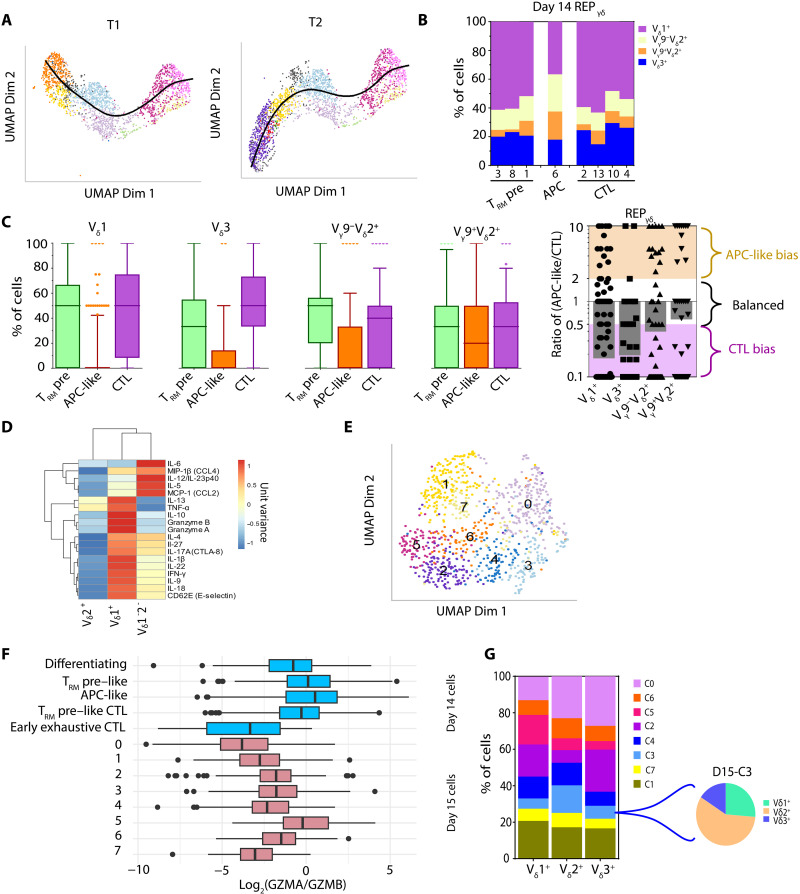
Effector differentiation trajectory of CB_γδ_ in REP culture. (**A**) Slingshot inferred differentiation trajectories (T1 and T2) of CB_γδ_ in REP culture. (**B**) Percentage of the indicated clonotypes of REP_γδ_ within each of the D14 cell clusters. (**C**) Percentage of T_RM_ precursor–like cells, APC-like cells, and CTL (left) and ratio of APC-like cells to CTL (right) within each individual REP_γδ_ clone of the indicated clonotype (V_δ_1^+^, V_δ_3^+^, V_γ_9^−^V_δ_2^+^, and V_γ_9^+^V_δ_2^+^). Left: Line within box indicates the median, with box limits extend from the 25th to 75th percentiles of all clones. Whiskers are drawn down to the 10th percentile and up to the 90th. Right: Each point represents single-clone data, and bar shows the geometric mean value of all clones within the same clonotype. (**D**) Relative amount of the indicated human cytokine secreted into D14-REP culture supernatant. Cytokines and γδ T cell subtypes (V_δ_1^+^, V_δ_ 2^+^, and V_δ_1^−^2^−^) were clustered using Euclidean distance and average linkage. MIP, macrophage inflammatory protein (**E**) UMAP using scRNA-seq of D15, K562-exposed cells. (**F**) Boxplot showing the ratio of *GZMA* to *GZMB* in annotated D14 cell types and D15 cell clusters. Line within box indicates the median, with box limits extend from the 25th to 75th percentiles of all cells. Whiskers capture scores within 1.5× of the interquantile range. Cells with scores larger or smaller than the range are plotted individually. (**G**) Left: Distribution of V_δ_1^+^/2^+^/3^+^ γδ T cells across all D15 cell clusters. Right: Proportion of V_δ_1^+^/2^+^/3^+^ γδ T cells within D15-C3.

Previous studies in PB_γδ_ found that V_δ_1^+^ cells showed distinct cytotoxic hallmarks (*46*) and were functionally more cytotoxic than V_δ_2^+^ cells at the population level (*47*). Leveraging on the single-cell in vitro CB_γδ_ differentiation data obtained in this study, we sought to examine whether the reported differences between γδT cell subtypes could be attributed to differential effector cell state acquisition. scRNA-seq–guided trajectory inference identified two major differentiation paths in REP_γδ_, tracing the transition of the early differentiating cells through the T_RM_ precursor–like cell state to either APC-like (T1) or CTL cell states (T2; Fig. 5A). Mapping of TCR clonotype across the different cell states showed preferential enrichment of V_δ_2^+^ cells in the APC-like cell cluster (C6), with 43% of these V_δ_2^+^ cells also being V_γ_9^+^ (Fig. 5B). This suggested that V_δ_2^+^ (especially V_γ_9^+^V_δ_2^+^) cells had enhanced ability to differentiate into the less cytotoxic APC-like cells compared to V_δ_1/3^+^ cells. At the clonal level, most γδ T cell clones regardless of their V_δ_ subtype generated both cell states as they differentiate in REP culture. However, we observed that majority of V_δ_1^+^ and V_δ_3^+^ clones were bias toward producing CTL than APC-like cells (Fig. 5C). This differentiation bias was more pronounced among large (>10 cells), adaptive-like V_δ_1^+^ and V_δ_3^+^ clones as we did not observe any such clones being APC-like bias (fig. S5A, top). In contrast, a more balanced mix of both APC-like bias and CTL bias clones was found within V_δ_2^+^ cells, especially among V_γ_9^+^V_δ_2^+^ clones (Fig. 5C, right, and fig. S5A). Together, our data highlighted the enrichment of CTL bias clones specifically among V_δ_1/3^+^ cells, contributing to their overall increased cytotoxic activities over V_δ_2^+^ cells. When we separately expanded FACS-purified V_δ_1^+^/2^+^/1^−^2^−^ cells (*n* = 4), we confirmed that the expanded V_δ_2^+^ cells produced relatively lower level of a whole range of cytokines compared to V_δ_1^+^/1^−^2^−^ cells (Fig. 5D and table S16).

We also asked whether the preferential acquisition of CTL bias differentiation program by V_δ_1/3^+^ clones was induced specifically by the EBV antigens presented on the feeder cell line. To address this, we further challenged D14 REP_γδ_ cells with EBV-negative K562 leukemic cell line. After quantification of gene expression, we performed clustering of both K562-challenged (D15) and D14 REP_γδ_ cells. Changes in transcriptomic profile were observed following 24 hours of exposure of to K562 (fig. S5B, left). Cells were well separated by treatment condition, and there was an enrichment of genes involved in various immunoregulatory processes including effector differentiation and antigen presentation in the D15, K562-exposed population (fig. S5B, middle and right panels, and table S17). This confirmed that D14 REP_γδ_ were cross-reactive to K562. We next performed reclustering and low-dimensional embedding of the D15 cells, identifying eight cell populations following K562 challenge (Fig. 5E). To examine the cytotoxic potential of these cells, we evaluated their relative *GZMA*/*B* expression and observed that majority of D15 cell clusters had a low *GZMA*/*GZMB* expression ratio resembling that of D14-CTL (Fig. 5F). Compared to D14 REP_γδ_, D15 cells showed a general increase in *GZMB* expression while maintaining high *GZMA* expression (fig. S5C). These data suggested further effector differentiation of D14 REP_γδ_ into D15 cells upon K562 exposure, resulting in a more homogeneously cytotoxic population. In line with this, one of the D15 cell clusters (C0) emerged to show an NK-like dysfunctional T cell phenotype (*42*), with elevated cell surface expression of NK receptors (NKG2D, NKp30, and CD56) and immune checkpoint receptors [T cell immunoglobulin and mucin domain-containing protein 3 (TIM3), lymphocyte activating 3 (LAG3), and programmed cell death protein 1 (PD1); fig. S5D]. Consistently, when we compared the expression profiles of D15 cell clusters to the GSs of our previously identified D14 REP_γδ_ cell states (differentiating; T_RM_ precursor–like, APC-like, T_RM_ precursor–like CTL, and early exhaustive CTL), we also found that D15-C0 showed a high resemblance to early exhaustive CTL (fig. S5E). On the other hand, the transcription signature of D15-C3 was particularly similar to APC-like cells (fig. S5E), whereas the remaining D15 cell clusters were more like T_RM_ precursor–like CTL (fig. S5E). Notably, in addition to differentiated effector GS, all D15 cell clusters also displayed GS of less differentiated (T_RM_ precursor–like/early differentiating) cell states (fig. S5E). These data advocated that the bulk of the cells present in culture for at least 24 hours after K562-challenge was likely derived from recent differentiation of D14-early effectors/precursors and not contributed by the persistence of D14 mature effectors.

Clonotype analysis revealed that V_δ_2^+^ comprised a much higher fraction of D15-C3 cells (15.1% of V_δ_2^+^ versus 5.6 and 7.1% of V_δ_1^+^ and V_δ_3^+^, respectively), resulting in an enrichment of V_δ_2^+^ (57.9%) within D15-C3 (Fig. 5G and tables S18 and S19). To further rule out that V_δ_2^+^ enrichment was contributed by the persistence of residual D14-V_δ_2^+^ APC-like cells in culture, we turned to focus on only D15 cells with a T_RM_ precursor–like expression profile (i.e., less differentiated than APC-like cells) and found similar observations (fig. S5F and tables S18 and S19). Together, our data showed that both EBV-LCL and K562 stimulation induced a discordance in effector differentiation between γδ T subtypes in vitro, with the preferential acquisition of a CTL bias differentiation program specifically by V_δ_1^+^ and V_δ_3^+^ cells.

## DISCUSSION

ACT relies heavily on in vitro cell expansion to generate sufficient immune effector cells to induce treatment response. In the γδ T cell space, success in P-Ag–induced V_δ_2^+^ cell expansion has led to the development of γδ T cell–based cancer immunotherapy (*48*). However, past suboptimal clinical experiences in V_δ_2^+^-based cancer therapy trials (*13*) coupled with the lack of characterization of the different subtypes of γδ T cells had significantly impeded its clinical application. We argue that better characterization of both the starting cell source and in vitro–generated γδ T cell products with respect to its repertoire composition and functional and exhaustion status can guide process design to improved potency.

In this study, we demonstrated a scalable in vitro expansion of all subtypes of CB_γδ_ using a modified REP culture. Comparison between parallel CB_γδ_ and PB_γδ_ cultures confirmed the significantly higher expansion potential in the former due to their less differentiated nature. A substantial fraction of the expanded CB_γδ_ displayed a T_SCM_-like phenotype that has been associated with superior antitumor activity in adoptively transferred CD8^+^ T cells (*37*, *49*, *50*). Consistent with earlier studies (*16*, *32*), we showed that both innate- and adaptive-like expansion characteristics are differentially adopted by various γδ T cells subtype. Moreover, such cell expansion traits can also be correlated to their preexisting transcription cell states. Specifically, our data confirmed that CB-derived V_γ_9^+^V_δ_2^+^ cells were associated with a strongly innate-like transcription program as opposed to other γδ T cell clonotypes spreading more heterogeneously across the adaptive-innate axis. This has implications in expanded γδ T cell product repertoire complexity, dependent upon the relative ratio of innate- and adaptive-like γδ T cells present in the starting cell materials.

Progressive differentiation and acquisition of heterogeneous functional states were seen across the expanded cells in REP culture and could potentially recapitulate the developmental trajectories of newborn γδ T cells following antigen encounter as previously described (*29*, *51*, *52*). Further investigations are required to elucidate how the different developmental programs are initiated and regulated. Nevertheless, our REP culture starting from CB_γδ_ produced a spectrum of cells capable of eliciting complementary functions at the population level, including CTL, T_RM_ precursor–like, and APC-like cells. The presence of T_RM_ precursor–like cells suggests preferential tissue-homing ability and is a beneficial characteristic for adoptive cell therapy products. In addition, these cells also showed a high expression of *GZMA* that has been demonstrated to display noncytotoxic immune regulations (*53*, *54*), enhancing the role of these T_RM_ precursor–like cells in orchestrating tissue homeostasis and repair that are often attributed to V_δ_1^+^ cells (*55*). On the other hand, antigen-presenting V_δ_2^+^ cells have also been previously reported to induce both CD4 and CD8 T cell response (*56*, *57*). Thus, adoptive transfer of these cells may have the potential to cross activate host immunity to elicit a sustained treatment response.

The γδ T cell subtype–specific differentiation programs observed in our cultures substantiate previous observations in PB_γδ_ (*46*). In particular, the preferential adoption of CTL bias differentiation program displayed by V_δ_1/3^+^ cells provides a mechanistic insight to early observations that both freshly isolated and in vitro–expanded V_δ_1^+^ cells were relatively more cytotoxic compared to V_δ_2^+^ cells (*47*). Our data suggest that the observed differences in differentiation programs are not solely dictated by external stimuli but are more likely dependent upon preexisting transcriptional programs inherent to the specific γδ T cell subsets. With a predominantly innate-like expression profile particularly among V_γ_9^+^V_δ_2^+^ cells, one could speculate that the mostly small-sized CTL and APC-like bias V_γ_9^+^V_δ_2^+^ clones were derived from the two major innate-like cell clusters D0-C11 and D0-C7, respectively. The lack of D0-C11 cells among V_δ_1/3^+^ suggested that CTL bias V_δ_1/3^+^ clones were mainly derived from adaptive-like D0 (C5/9) cells, whereas V_δ_1/3^+^ D0-C7 cells could have similarly contributed to the small APC-like bias V_δ_1/3^+^ clones. This could also explain why no APC-like bias clones were seen among large V_δ_1/3^+^ clones. Further investigations are required to validate such causal relationship. Despite variable differentiation programs, note that our clonal tracing confirmed that majority of the activated γδ T cell clones followed more than one differentiation trajectories to generate multiple effector cell types. This implies that, even in an extreme clonally focus γδ T cell expansion culture, functional diversity is always seen among the expanded cells, although the proportion of cells with different cell states can vary significantly between γδ T cell clonotypes. In this regard, one can deduce that, while functional efficacy in cancer targeting is likely dictated by the specific TCR-clonotype, the overall potency of conventionally expanded V_δ_2^+^- and V_δ_1^+^- focused cell products will be determined by an ideal cell state ratio that is probably context or disease dependent. With respect to this, studies in CD8^+^ T cells have already shown that less differentiated effectors (*37*, *49*) or even a more primitive cell source of effectors (*50*) make more potent adoptive cell therapy products.

In summary, we provide evidence to show that CB_γδ_ comprising a complex TCRγδ repertoire with variable innate- and adaptive-like cell states is an attractive alternative immune cell type for ACT. Our identification of transcriptionally distinct cell states among both pre- and post-culture expanded γδ T cells calls for more robust characterization of the starting cell source and expanded cell products, with the larger implication that specific cell state compositions can confer superior cancer targeting potential in a context-dependent manner.

## MATERIALS AND METHODS

### Study design

To expand CB_γδ_ and PB_γδ_, REP cultures were set up for 14 to 21 days for a total of 28 CB and three PB samples. In vitro cytotoxic activity of REP_γδ_ was assayed for 12 batches of REP_γδ_ upon fresh cell harvest at D14 and D21 of culture. Cytokine secretions were assayed for three batches of REP_γδ_. In vivo tissue-homing capabilities and cytotoxic activities against human AML cells were assayed for two and four batches of the REP_γδ_, respectively. To determine heterogeneity in γδ T cell clonal expansion kinetics, single–γδ T cell REP cultures were set up for four of the CB samples. To characterize the γδ T cell state composition and repertoire complexity upon expansion in REP cultures, scRNA-seq coupled with surface protein quantification and TCRγδ sequencing were performed in D14 REP_γδ_ generated from three different human CB samples using the 10X Genomics Chromium platform. Among these three samples, one of the samples also had aliquots of cells harvested at serial time points (D0 and D7 of REP culture as well as 1 day after K562 exposure, “D15”) for multiplex single-cell immune profiling.

### Primary human cells

CB from anonymized normal, full-term infants were obtained from Singapore Cord Blood Bank (SCBB), and the mononuclear cell (MNC) fraction was isolated by centrifugation on Ficoll-Paque PLUS (GE Healthcare, Uppsala, Sweden). Granulocyte colony-stimulating factor–mobilized PB from normal donors and BM MNC of primary AML patients (table S20) were obtained from the hematology repository in Singapore General Hospital. Human cell usage in this study was reviewed and approved by the SCBB Research Advisory Ethics Committee and the SingHealth Centralised Institutional Review Board. For cell surface phenotype analysis, cells were stained with the respective antibodies on ice for at least 30 min. All antibodies used for flow cytometry are listed in table S21. Flow cytometry analyses were performed with LSRFortessa Analyzer (BD Biosciences), and FACS was performed using FACSAria III (BD Biosciences). Data were analyzed using FlowJo 10.6.1 (Tree Star Inc.).

### Modified REP culture

γδ T cells were first enriched from CB MNCs using an EasySep Human γδ T cell isolation kit (STEMCELL Technologies) according to the manufacturer’s protocol. For separate expansion of V_δ_1^+^, V_δ_2^+^, and V_δ_1^−^2^−^ subsets, cells were first FACS-purified. Cells were then mixed with feeder cells consisting of irradiated normal human PBMCs and EBV-LCLs at the ratio of 1:50:100 in RPMI 1640 (Gibco) supplemented with 10% fetal bovine serum (FBS) and cytokine combo [recombinant human IL-2 (rhIL-2; 170 U/ml), rhIL-15 (25 U/ml), and rhIL-21 (0.7 U/ml), all from Miltenyi Biotec]. The cell mixture was first seeded onto plates precoated with 0.3 μg/cm^2^ pan TCR_γδ_ antibody (clone IMMU510 from Beckman Coulter) and cultured for 2 days. Subsequently, cells were transferred to G-Rex flasks (Wilson-Wolf, USA, for bulks cell or subset expansion) or noncoated 96-well plate (for single–γδ T clone expansion) and cultured for another 2 to 3 weeks according to the manufacturer’s protocol. Fresh cytokine comb was added to the culture every 2 to 3 days. At weekly interval, the total number of γδ T in culture was enumerated with the supplementation of irradiated PBMCs and EBV-LCLs at the ratio of 1:10:20, respectively. To reduce the volume of cells in culture, only a fraction of the cells was reseeded back into G-Rex flasks for another week of culture. Overall fold expansion and absolute number of γδ T cell at final cell harvest were calculated, assuming that all cells were expanded.

### In vitro cytotoxic assay

Target cells were labeled with ^51^Cr for 2 hours before being co-cultured with the expanded γδ T cells at various E:T cell ratios for 5 to 6 hours. Radioactive counts in the supernatant were then read with a Wallac Wizard 1470 gamma counter (PerkinElmer). Negative and positive controls were determined in target cells with no treatment and treated with 2% Triton X-100, respectively. Percent specific cytotoxicity was determined as [(sample-negative control)/(positive control-negative control)] × 100%. For all cell line targets, duplicate wells were set up, and the average radioactive counts were recorded for each E:T ratio within the same experiment. Graph shows the means ± SEM of at least three independent experiments. For each primary cell target, single well was set up for each E:T ratio in a single experiment.

### In vitro cytokine secretion profiling

Harvested REP_γδ_ were first washed and resuspended in fresh RPMI 1640 (Gibco) supplemented with 10% FBS and rhIL-2 (170 U/ml; Miltenyi Biotec). Cells were then incubated at 37°C for 1 day before culture supernatant was collected. Secreted cytokines were measured with a Luminex assay using ProcartaPlex customized human cytokine 19-plex panel [IL-1β, IL-4, IL-5, IL-6, IL-9, IL-10, IL-13, IL-17A, IL-18, IL-22, IL-12/IL-23 (p40), IL-27, IFN-γ, TNF-α, CCL2, CCL4, CD62E, granzyme A, and granzyme B; Thermo Fisher Scientific] according to the manufacturer’s instructions. This sandwich reaction occurred in wall-less DropArray (DA)–Bead plates (Curiox) according to the manufacturer’s instructions (https://youtu.be/P6ShRhE1GKE). Briefly, the wall-less DA-Bead plate was blocked with Dulbecco's phosphate-buffered saline (dPBS) containing 1% bovine serum albumin for 30 min. Ten microliters of culture supernatant was loaded onto the plate with preloaded magnet beads and incubated for 2 hours. The reaction was further incubated with 10 μl of detection antibody for 1 hour and 5 μl of streptavidin-phycoerythrine for 30 min. All incubations occurred on a 1-cm span orbital shaker at 385 rpm at room temperature. The plate was washed three times in the LT-MX washer between these three incubation steps. Plates were read on a Milliplex analyzer (Millipore) with Luminex xPONENT software. Calculated cytokine concentrations (picograms per milliliter) for each sample (*n* = 4 × 3 γδ T cell subtypes) were fed into ClustVis (*58*) for relative comparison visualized in heatmap. For each cytokine, median of the four samples within each γδ T cell subtype was taken. Values on each row (cytokine) were centered, and unit variance scaling was applied. Both rows (cytokines) and columns (γδ T cell subtypes) were clustered using Euclidean distance and average linkage.

### In vivo assays in immunodeficient mice

NSG mice were purchased from the Jackson Laboratory and maintained in Vivarium in Duke-NUS Medical School. Six- to 10-week-old mice were sublethally irradiated with 220 cGy of 137Cs γ-rays before transplantation. All animal experiments were performed with the approval of the SingHealth Institutional Animal Care and Use Committee, and experiments were performed in accordance with the SingHealth Institutional Animal Care and Use Committee guidelines. For hematopoietic repopulation, between 10^6^ and 10^7^ CB or AML cells were intravenously injected into mice within 24 hours after irradiation. Expanded γδ T cells were infused into mice via tail vein according to different experimental setups. For GvHD studies, 1 × 10^5^ CD34-enriched CB cells were infused into mice alone or together with 1 × 10^7^ to 2 × 10^7^ REP_γδ_. In the control group, between 1 × 10^7^ and 2 × 10^7^ PBMCs were infused into mice. In coinfusion experiments, expanded REP_γδ_ were intravenously injected into mice between 30 and 120 min after the first cell infusion (either CB or AML). For treatment of preestablished AML-PDX, 1 × 10^7^ (for targeting against BM845) or 2.6 × 10^7^ (for targeting against BM1043) expanded REP_γδ_ were infused 1 to 2 months after AML cell transplantation. Mice were then analyzed at 3 to 4 weeks after REP_γδ_ infusion. In all mice, rhIL-2 of 6.4 × 10^6^ U/kg, rhIL-15 of 1.3 × 10^5^ U/kg, and rhIL-21 of 2 × 10^3^ U/kg were intraperitoneally injected twice a week starting from day 2 after REP_γδ_ infusion (all cytokines from Miltenyi Biotec).

### Mouse sample analysis and secondary transplantation

For REP_γδ_ tissue-homing experiments where absolute human cell counts were computed, BM cells collected from two tibias and two femurs, 25% of whole mouse LV, whole mouse SPN, and 500 μl of mouse PB were used for further processing for flow cytometry analysis. Single-cell suspensions of mouse BM, LV, and SPN samples in staining buffer (phosphate-buffered saline and 2% FBS) were obtained by vortexing or physical grinding and filtering over 0.4-μm cell strainer. Red blood cells (RBCs) were first lysed with 0.8% NH_4_Cl on ice for 5 min before staining with antibodies. Mouse PB samples collected in K3-EDTA–coated tubes were directly stained with antibodies before RBC lysis was performed. To enable absolute cell count calculation, AccuCheck counting beads (Invitrogen) were added to the sample before flow cytometry analysis was performed. Human γδT cells were defined as CD45^+^CD3^+^TCRγδ^+^, while human AML cells were defined as CD45^+^CD3^−^TCRγδ^−^ in REP_γδ_ tissue-homing experiments and CD45^+^CD15/33^+^CD19/20^−^CD3^−^ in all other mouse experiments. All AML-PDX models were validated to have repopulated exclusively with human myeloid cells, with undetectable level of human B (CD45^+^CD19/20^+^CD15/33^−^CD3^−^) and T (CD45^+^CD3^+^CD19/20/15/33^−^) cells before any REP_γδ_ treatment. For secondary transplantation, total BM cells were flushed from all long bones (two tibias and two femurs) and pelvis and processed as described above. For BM395, samples collected from primary mice within the same treatment group (three mice per group) were pooled together and depleted of mouse cells using an EasySep mouse/human chimera isolation kit (STEMCELL Technologies, no. 19849) before intravenous injection into secondary recipient mice (two secondary mice per group). For BM1043, because two of the four untreated mice and two of the three REP_γδ_-treated mice either had succumb to the disease or became moribund, BMs of the surviving mice at the point of harvest were separately transplanted into individual secondary mouse.

### Single-cell immune profiling library preparation

CB_γδ_ and REP_γδ_ harvested from bulk cultures were first stained with anti-CD3 and anti-TCR_γδ_ antibodies and purified by FACSAria III (BD Biosciences). The purified cells were then stained with a panel of TotalSeq-C antibodies (table S22) according to the manufacturer’s protocol and processed according to 10X Genomics’ standard workflow for Chromium Single Cell V(D)J with Feature Barcoding technology for Cell Surface Protein to generate the transcriptome (5′GEX) and featured barcode library. To generate the TCR_γδ_ V(D)J library, a two-step target enrichment PCRs were adopted from the previously published protocol (*59*). Libraries were pooled and sequenced on NovaSeq 6000.

### Statistical analysis

Sample sizes for various experimental analyses were indicated in the text descriptions in Results or figure legends. Three individual CB samples were used for day 14 single-cell profiling, with one of these also used for serial time-course (D0 and D7) analysis. Where applicable, mean and SEM were reported unless otherwise stated. Details of parameters used in the bioinformatics analysis are provided in Supplementary Materials and Methods.

## Supplementary Materials

### This PDF file includes:

Supplementary Materials and Methods Figs. S1 to S5 Tables S1 to S23 References
