# Supplementary material for: Cord blood–derived Vδ2+ and Vδ2− T cells acquire differential cell state compositions upon in vitro expansion
Source: Sci Adv. 2023 Jun 16;9(24):eadf3120. doi: 10.1126/sciadv.adf3120 (PMC10275585; doi:10.1126/sciadv.adf3120)
Supplement: Supplementary file 1 — Supplementary Materials and Methods Figs. S1 to S5 Tables S1 to S23 References [file sciadv.adf3120_sm.pdf]

Science Advances
AAAS

Supplementary Materials for

Cord blood derived $V_\delta 2^+$ and $V_\delta 2^-$ T cells acquire differential cell state compositions upon in vitro expansion

Jeremy Wee Kiat Ng *et al.*

Corresponding author: Alice Man Sze Cheung, alice.cheung@sgh.com.sg, alice.cheung@duke-nus.edu.sg

Sci. Adv. **9**, eadf3120 (2023)
DOI: 10.1126/sciadv.adf3120

This PDF file includes:

Supplementary Materials and Methods
Figs. S1 to S5
Tables S1 to S23
References

Supplementary Materials and Methods

TCRγδ spectratyping

Genomic DNA were extracted and purified from *in vitro* expanded $REP_{\gamma\delta}$ using QiaAmp DNA mini kit (Qiagen). Amplifications of the complementarity determining region 3 (CDR3) of TRG and TRD gene were performed using IdentiClone TCRG and TCRD Gene Clonality Assay (Invivoscribe) according to manufacturer's instructions. PCR products were size separated using ABI 3500 and data analyzed with the GeneScan software (Applied Biosciences).

scRNA-seq data processing

Single cell RNA-seq were pre-processed using the default Seurat pipeline. For surface protein (FB-seq), normalization was performed using centered log-ratio (CLR). 5'-GEX reads were aligned against the hg38 reference transcriptome provided by 10X Genomics. A reference for TRD and TRG genes was created using the IMGT database (accession date: December 2020) and used for clonotype identification. All data alignments and quantifications were performed using the multi pipeline in CellRanger. The following cells were removed prior to analysis: i: cells with only detectable TRG or TRD expression (ie: unpaired TCR) or unproductive TCRs, ii: cells with > 10% of reads mapping to mitochondria genes and iii: cells with less than 200 genes. A total of 4574 cells from 5 samples from 3 cords (3 samples at Day 14, and additional Day 0 and Day 7 sample from one cord) with paired RNA-seq and TCR-seq were retained for downstream analysis following filtering. All configuration files, data and scripts are available upon request from the author.

Cell cluster analysis

Samples from the same experiment were combined by merging, while samples between different experiments were combined by using the integration method implemented in Seurat (v4.0.2) (60). Cell cycle scores of individual cells were computed using the ScoreCellCycle function implemented in Seurat (v4.0.2), and cell cycle stage was assigned based on the S and G2M scores using the default setting in Seurat. Three thousand most variable genes were identified, and their expression scaled by regressing out the cell cycle score. Thereafter, dimension reduction was performed using Principal Component Analysis (PCA). The first twenty principal components were then used for low dimension embedding using Uniform Manifold Approximation and Projection (UMAP) with default parameters. Cell clustering was performed using the Louvain algorithm implemented in Seurat with a resolution of 1.2. Marker gene analysis was performed using the FindAllMarkers function implemented in Seurat. The raw counts were used for differential gene expression (DEG) analysis. DEG testing was performed using the MAST algorithm implemented in MAST, and only genes with an adjusted p-value of < 0.05 were considered as being differentially expressed. Adjustment for multiple hypothesis testing was done using the FDR. Only upregulated genes with a fold change of more than 1.5 and expressed in more than 90% of cells in the cluster were considered as cluster markers. Gene Ontology (GO) enrichment for each cell cluster was performed using the PANTHER webserver. Enrichment analysis was performed using the Fisher Exact Test. Multiple testing correction was using the False Discovery Rate (FDR).

Calculation of innateness score

Innateness score was calculated as previously described by Gutierrez-Arcelus et al (34). Reference data in the form of raw count matrix and metadata was downloaded from Gene Expression Omnibus (GEO accession: GSE124731). Cells annotated as being $V_\delta1$ and $V_\delta2$

were removed from the reference dataset. The reference dataset was then processed as described by as previously described (34). Briefly, gene expression was scaled, and the top 1545 most variable genes were used for dimension reduction. Dimension reduction was performed using PCA, and the first twenty principal components were used for UMAP embedding. Clustering was performed using the Louvain algorithm using default parameters. Following visual inspection to ensure that cells were well clustered, the loading score for each gene in the first principal component was extracted. The innateness score for each cell is calculated using the following formula:

$$\mathrm{Score} = \sum_{n=1}^{k} (\mathrm{Loading}_k \times \mathrm{Expression}_k)$$

Where $\mathrm{loading}_k$ and $\mathrm{expression}_k$ refers to the loading and scaled expression for the k-*th* gene.

Gene module (GM) analysis

To identify gene modules (GM), marker genes of all cell clusters were used as an input into Monocle3 (61) for gene clustering using the Louvain algorithm with a resolution of 0.1. Scores for each GM in each cell cluster were calculated by aggregating the expression scores of all gene member in all cells of the same cluster in Monocle3.

Label transfer analysis and gene signature scoring

Both reference and query datasets were pre-processed separately using SCTransform. Following pre-processing, transfer anchors were identified using the first thirty principal components for embedding. Label transfer was performed using the standard label transfer workflow in Seurat. Finally, labels of the highest scoring reference cell cluster/group were then transferred into individual query cell using the identified transfer anchors. Gene signature

scoring was performed using the AddModuleScore function in Seurat. The reference gene signatures used in this study are listed in Table S23.

Trajectory inference

Trajectory inference was performed using Slingshot (61) (v1.8.0) on the UMAP embedding produced by Seurat following cluster analysis. Cell cluster 9 (C9) was used as the starting point for trajectory inference. Slingshot infers principal curves, which describe the trajectory of a cell lineage (62). Cells on each principal curve were extracted for visualization. Visualization was performed using ggplot2 as a custom R-script.

Supplementary Figure 1

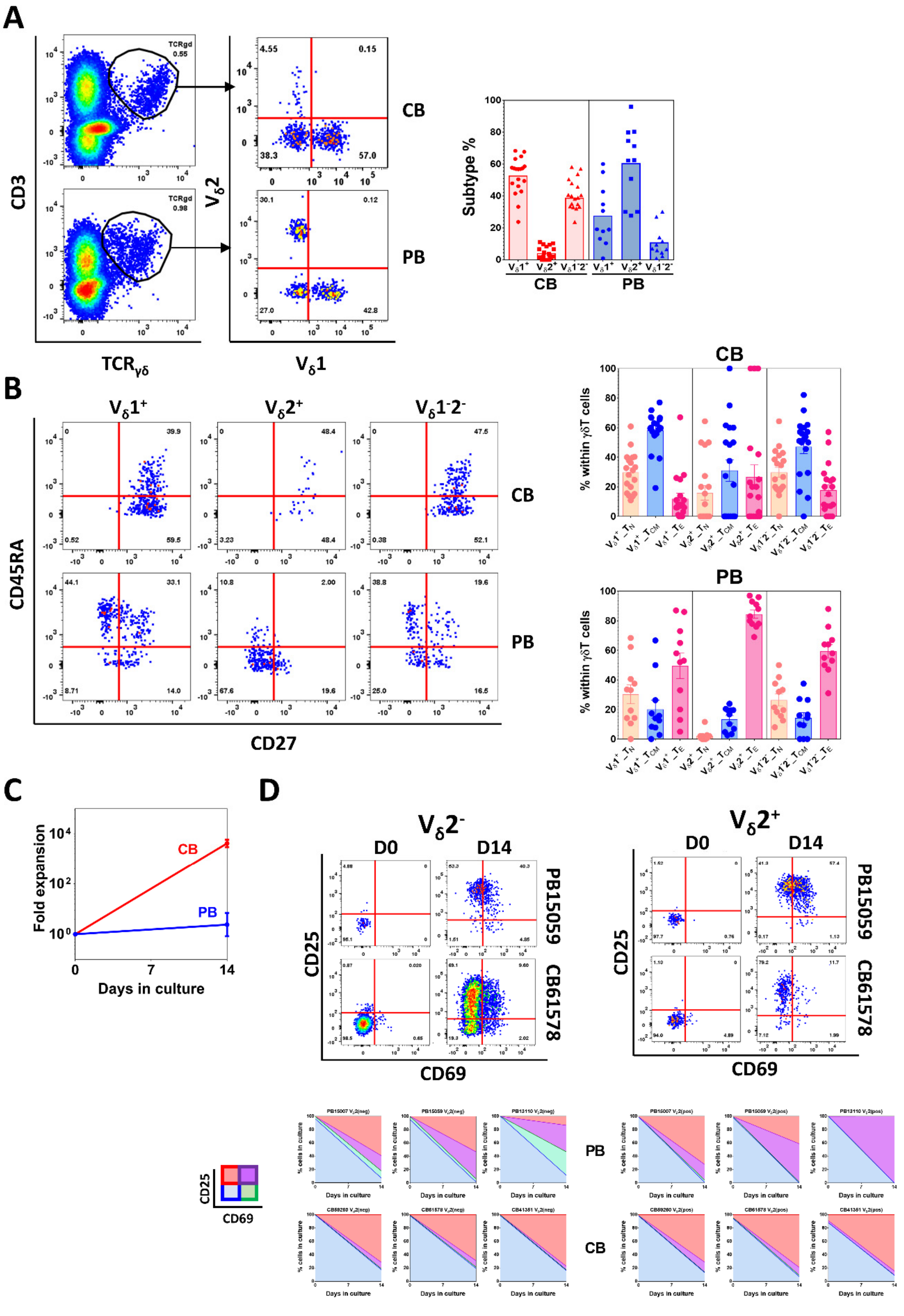

Supplementary Figure 1

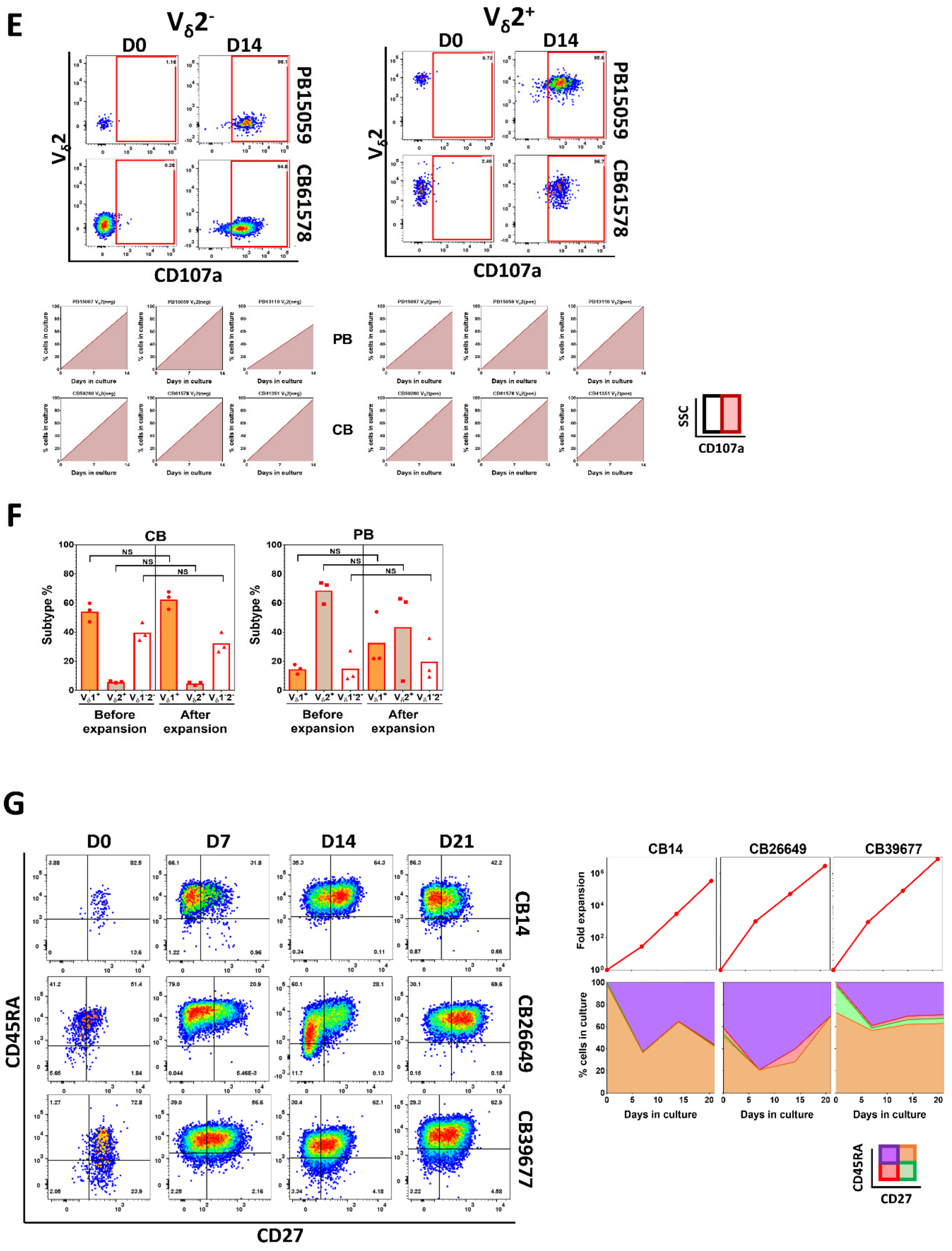

Supplementary Figure 1

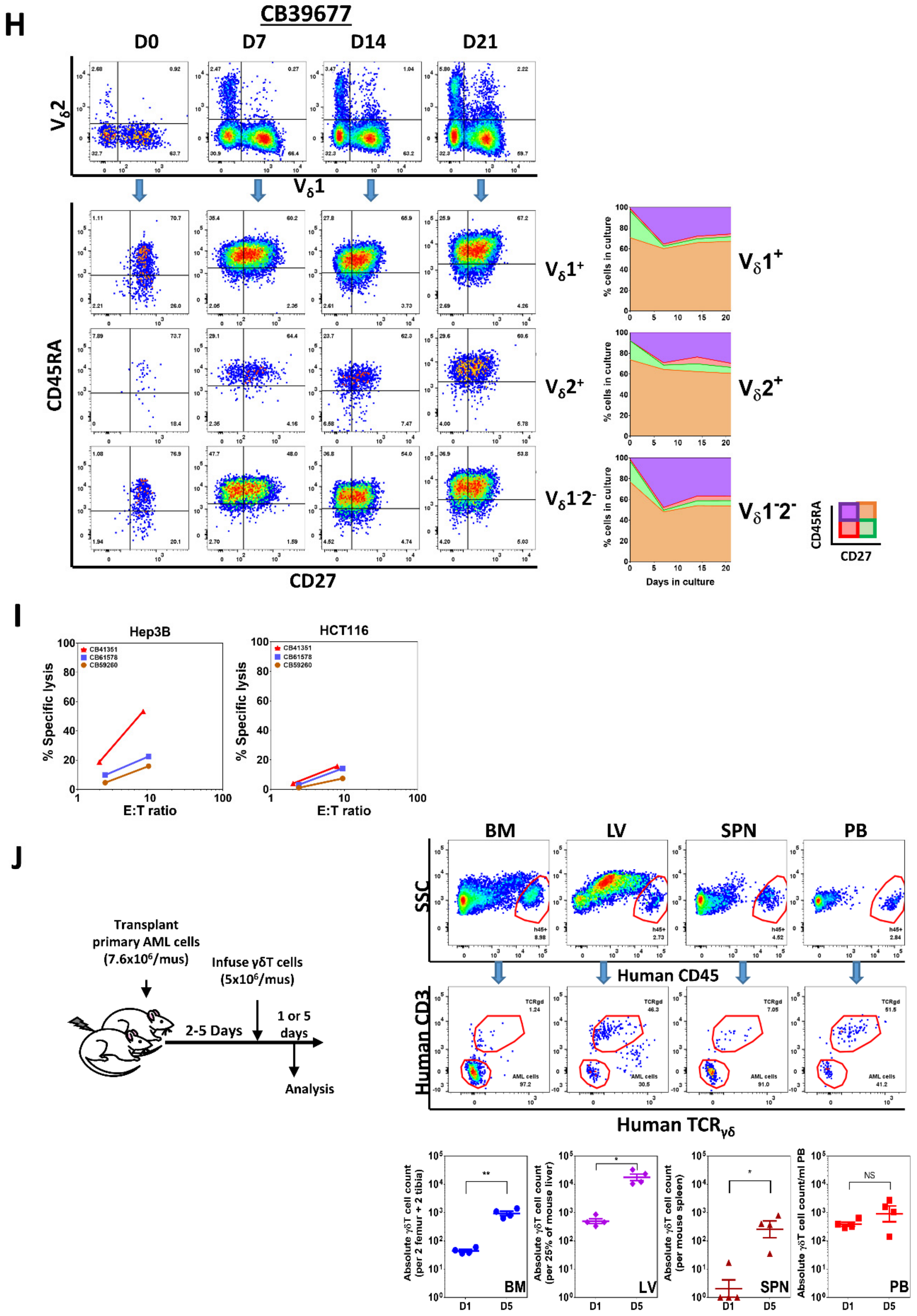

Supplementary Figure 1

K

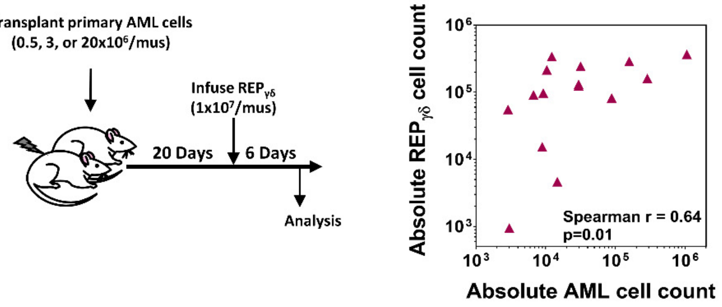

L

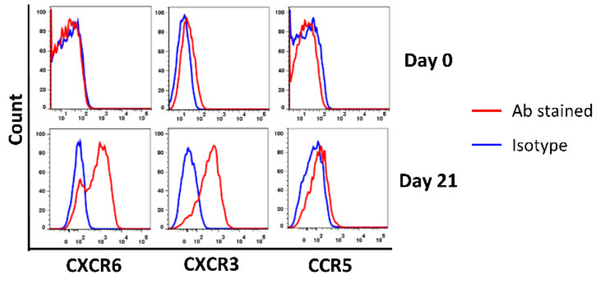

M

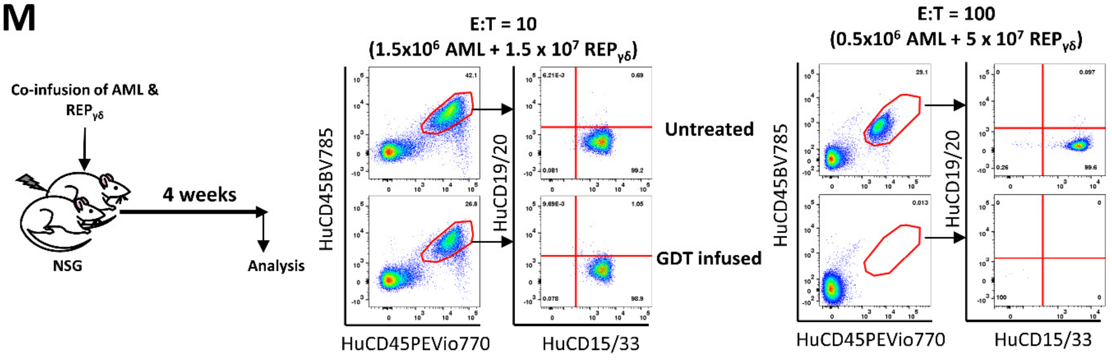

N

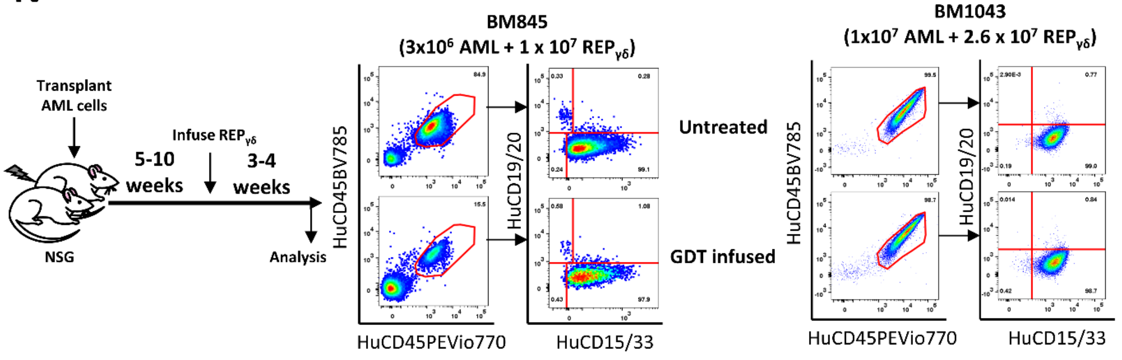

Supplementary Figure 1

O

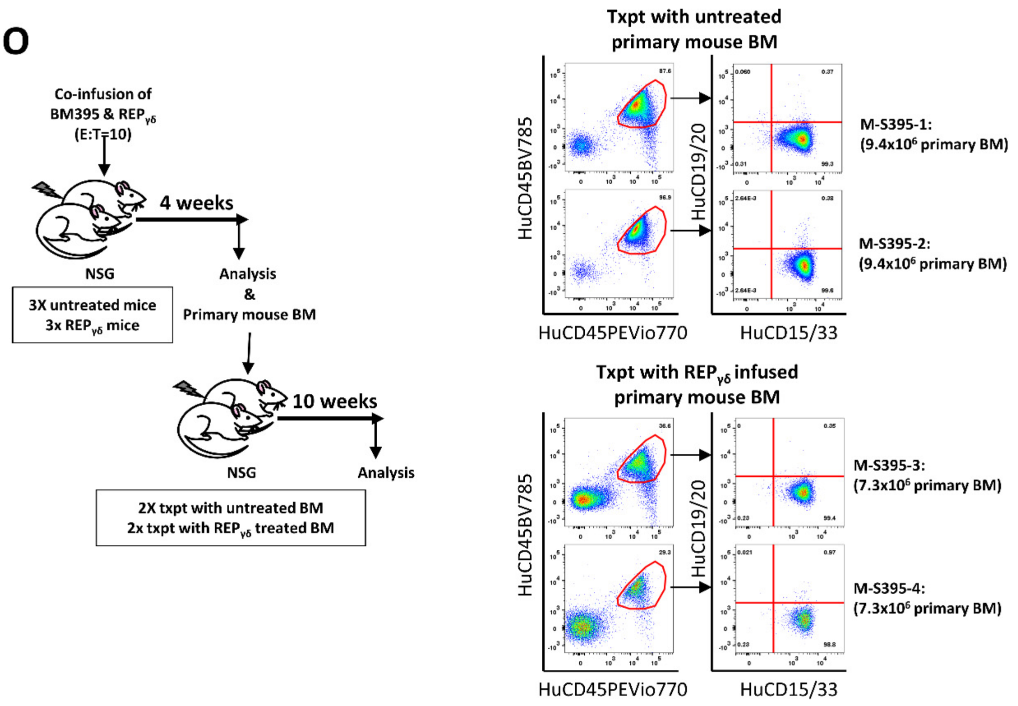

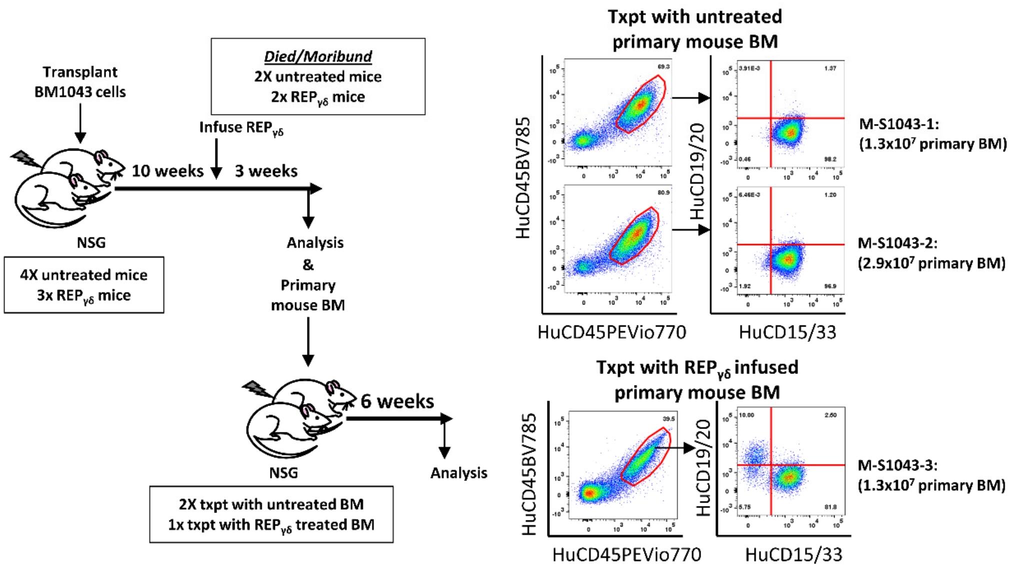

Supplementary Figure 1

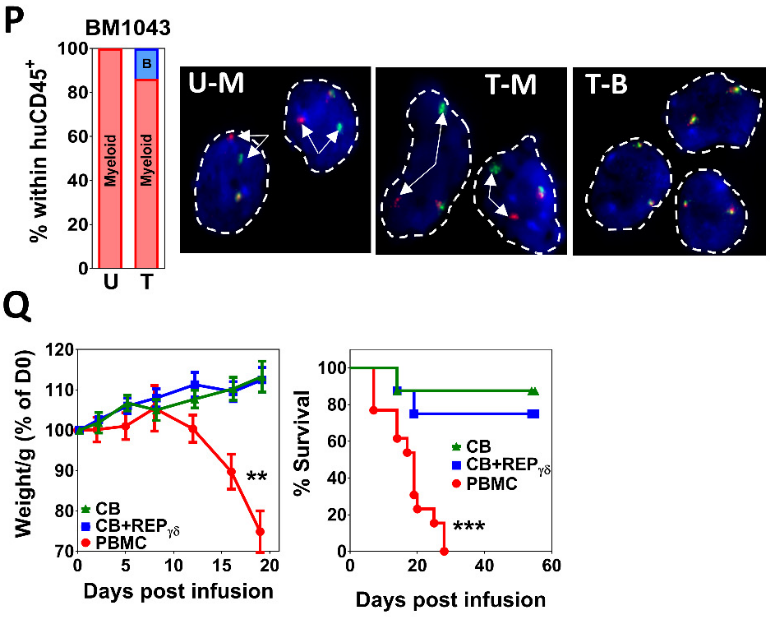

Fig S1. REP culture expansion of CB$_{\gamma\delta}$.

(**A & B**) Representative FACS plots (left panels) and percentage of the Vδ1$^+$, Vδ2$^+$ and Vδ1$^-$/2$^-$ cells (A, right panel) or percentage of T$_N$ (CD45RA$^+$CD27$^+$), T$_{CM}$ (CD45RA$^-$CD27$^+$) and T$_E$ (CD45RA$^{+/-}$CD27$^-$) cells (B, right panel) among CB$_{\gamma\delta}$ (n=19) and PB$_{\gamma\delta}$ (n=11). (**C**) Average (± standard error of mean, SEM) fold expansion of CB$_{\gamma\delta}$ and PB$_{\gamma\delta}$ (n=3 each) over 14 days in REP cultures. (**D & E**) Representative FACS plots (top panels) and proportion of Vδ2$^-$ or Vδ2$^+$ cells with differential cell surface expression of CD25 and CD69 (D, bottom panel) or CD107α (E, bottom panel) in individual CB$_{\gamma\delta}$/PB$_{\gamma\delta}$ sample on D0 and D14 days of REP cultures (n=3 each). (**F**) Vδ1$^+$, Vδ 2$^+$ & Vδ1$^-$2$^-$ subtype composition in CB$_{\gamma\delta}$/PB$_{\gamma\delta}$ before and after 14 days *in vitro* expansion (n=3 each). (**G & H**) Representative FACS plots (left panels), fold expansion of CB$_{\gamma\delta}$ (G, top right panel) and cumulative percentage of CD45RA$^+$CD27$^+$, CD45RA$^-$CD27$^+$, CD45RA$^-$CD27$^-$ and CD45RA$^+$CD27$^-$ cells (G, bottom right panel and H, right panel) over 21 days of REP cultures within total CB$_{\gamma\delta}$ (G) or Vδ1$^+$, Vδ 2$^+$ & Vδ1$^-$2$^-$ subset (H) of individual CB

sample. (**I**) Chromium release assay of Hep3B and HCT116 at the indicated effector to target cell (E:T) ratio. Graph shows the mean of radioactive counts from triplicate wells for each E:T ratio. (**J**) Schematic of *in vivo* $REP_{\gamma\delta}$ tissue homing experiment (left panel). Representative FACS plots showing the detection of human AML and $REP_{\gamma\delta}$ (top right panel) as well as the calculated absolute number of $REP_{\gamma\delta}$ in the indicated mouse tissue (bottom right panel). (**K**) Experimental schematic of *in vivo* chemotaxis of $REP_{\gamma\delta}$ towards xenografted human AML cells (left panel). Calculated absolute number of human AML and $REP_{\gamma\delta}$ in the spleen (SPN) of $REP_{\gamma\delta}$ infused AML-PDX, n=15. (**L**) Representative FACS plots of CXCR6, CXCR3 and CCR5 cell surface staining in non-manipulated (D0) $CB_{\gamma\delta}$ and D21-$REP_{\gamma\delta}$. (**M & N**) Experimental schematic of co-infusion of AML and $REP_{\gamma\delta}$ into NSG (M, left panel) and infusion of $REP_{\gamma\delta}$ into established AML-PDX (N, left panel). Representative FACS plots of human AML detection in BM of untreated and $REP_{\gamma\delta}$ infused mice (right panels). (**O**) Schematics of secondary transplantation of mouse BM engrafted with human AML cells (BM395 and BM1043) with or without *in vivo* exposure to $REP_{\gamma\delta}$ in primary mice (left panels). Representative FACS plots of human AML detection in BM of individual secondary mouse recipient (right panels). (**P**) Percentage of myeloid ($CD33^+$) and B ($CD19/20^+$) cells within the total repopulated human $CD45^+$ cells in secondary transplanted mice of BM1043 (left panel). U & T refers to mice transplanted with untreated and $REP_{\gamma\delta}$ infused primary mouse BM respectively. FISH of MLL gene translocation. Cell outline is marked by white dotted lines. White arrows marked the separation of the green and orange signal of the MLL dual colour break apart rearrangement probe, indicating the presence of MLL translocation (right panel). M & B refers to human myeloid and B cells respectively. (**Q**) Mice were infused with either 10^7 PBMC (n=12) or 10^4 allogeneic $CD34^+$ CB cells with (n=8) or without (n=8) 10^7 $REP_{\gamma\delta}$. The average body weight (± SEM) (left panel) and cumulative percentage of survival of mice in each group over time (right panel) were recorded.

Supplementary Figure 2

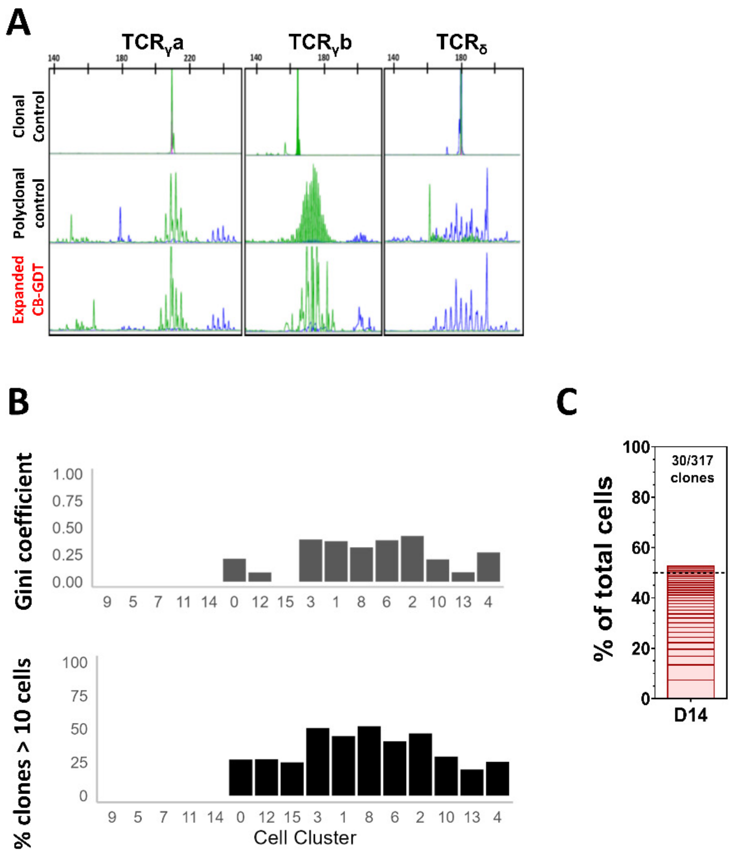

Fig S2. Expansion characteristics of $CB_{\gamma\delta}$.

(**A**) TCR_{γ} and TCR_{δ} repertoire analysis by standard PCR-based CDR3 spectratyping. (**B**) Gini coefficient reflecting the even-ness of γδ T cell clonotype distribution (top panel) and frequency of $CB_{\gamma\delta}$ clones with >10 cells (bottom panel) in each cell cluster. (**C**) Cumulative cell percentage of the top 30 largest γδ T clones (out of total 317 clones) in D14 REP culture of sample #47604.

Supplementary Figure 3

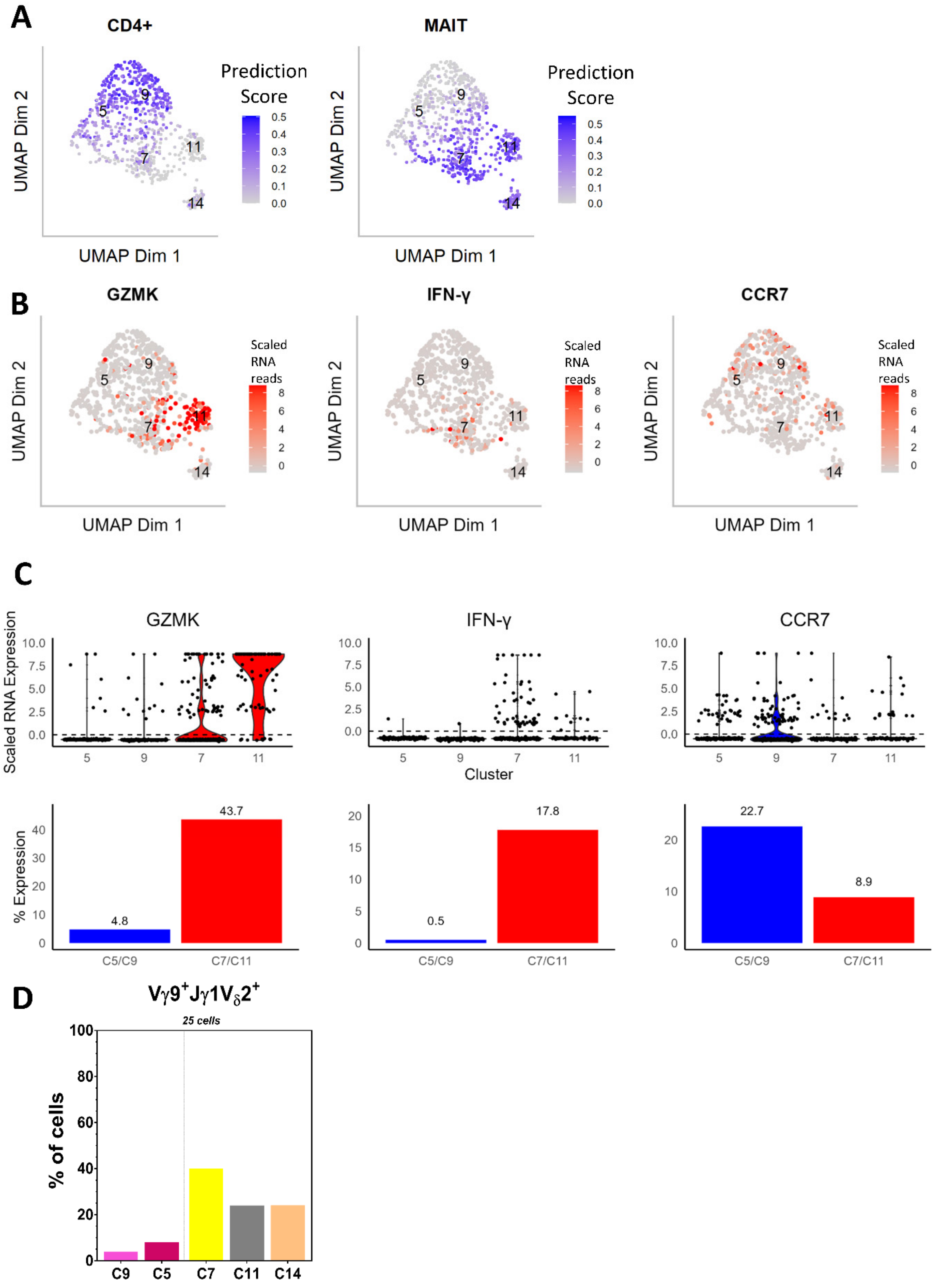

Fig S3. Innate- and adaptive-like transcriptome profile of $CB_{\gamma\delta}$.

(**A**) CD4 (left panel) and MAIT (right panel) cell prediction scores using Seurat label transfer in D0 $CB_{\gamma\delta}$. The reference dataset for CD4 and MAIT cells was obtained from previously published work (34). (**B**) Scaled RNA read counts of GZMK (left), IFN_{γ} (middle panel) and CCR7 (right panel) in D0 $CB_{\gamma\delta}$. (**C**) Top panels show scaled RNA expression of GZMK (left), IFN_{γ} (middle) and CCR7 (right). Each data point represents individual cell. The horizontal dashed line indicates the cut-off used for expression (>0). Bottom panels show the percentage of cells expressing the respective gene within C5/C9 (blue) and C7/C11 (red). (**D**) Distribution of $V_{\gamma}9^{+}J_{\gamma}1V_{\delta}2^{+}$ cells across D0 cell clusters.

Supplementary Figure 4

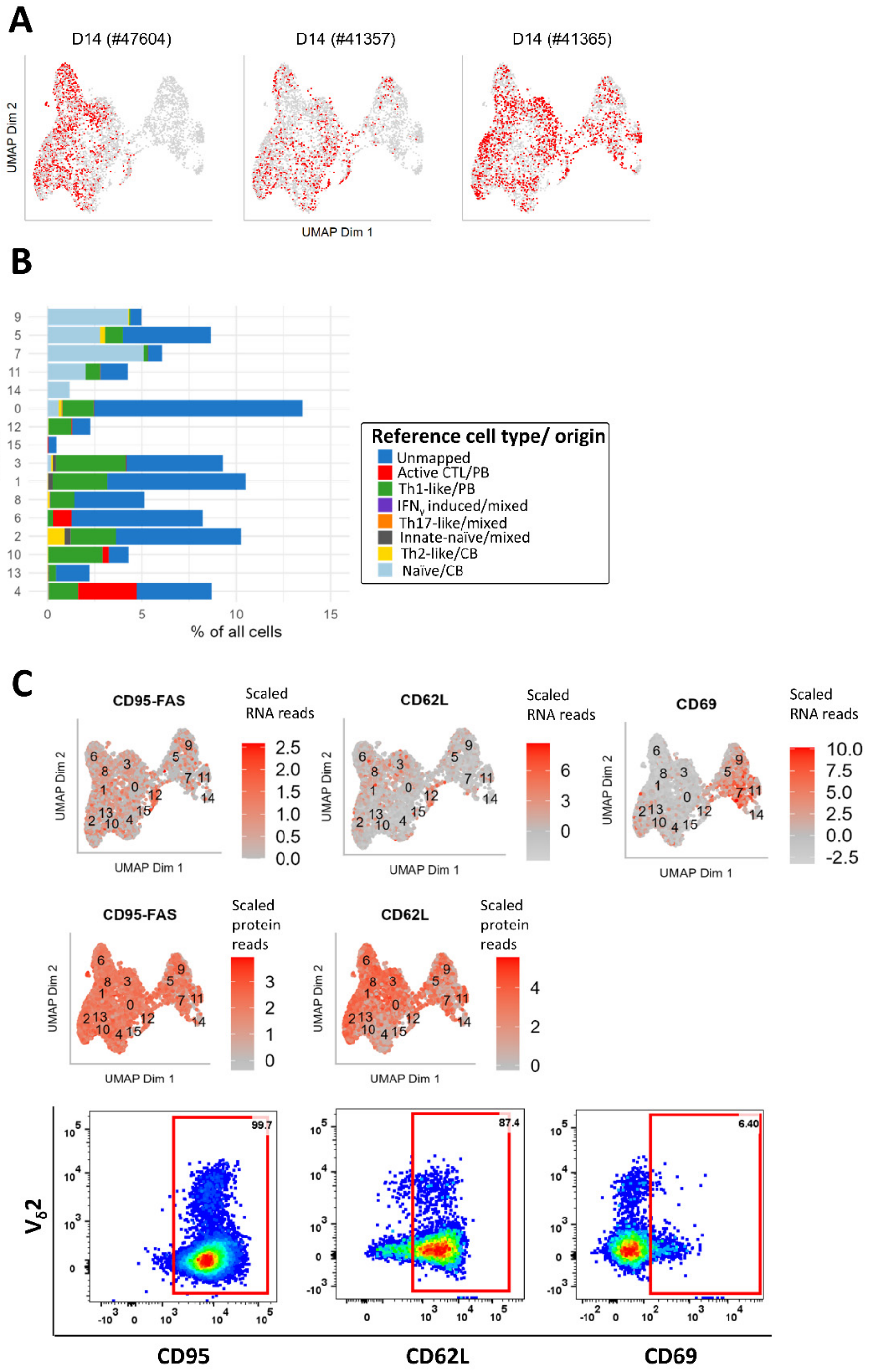

Supplementary Figure 4

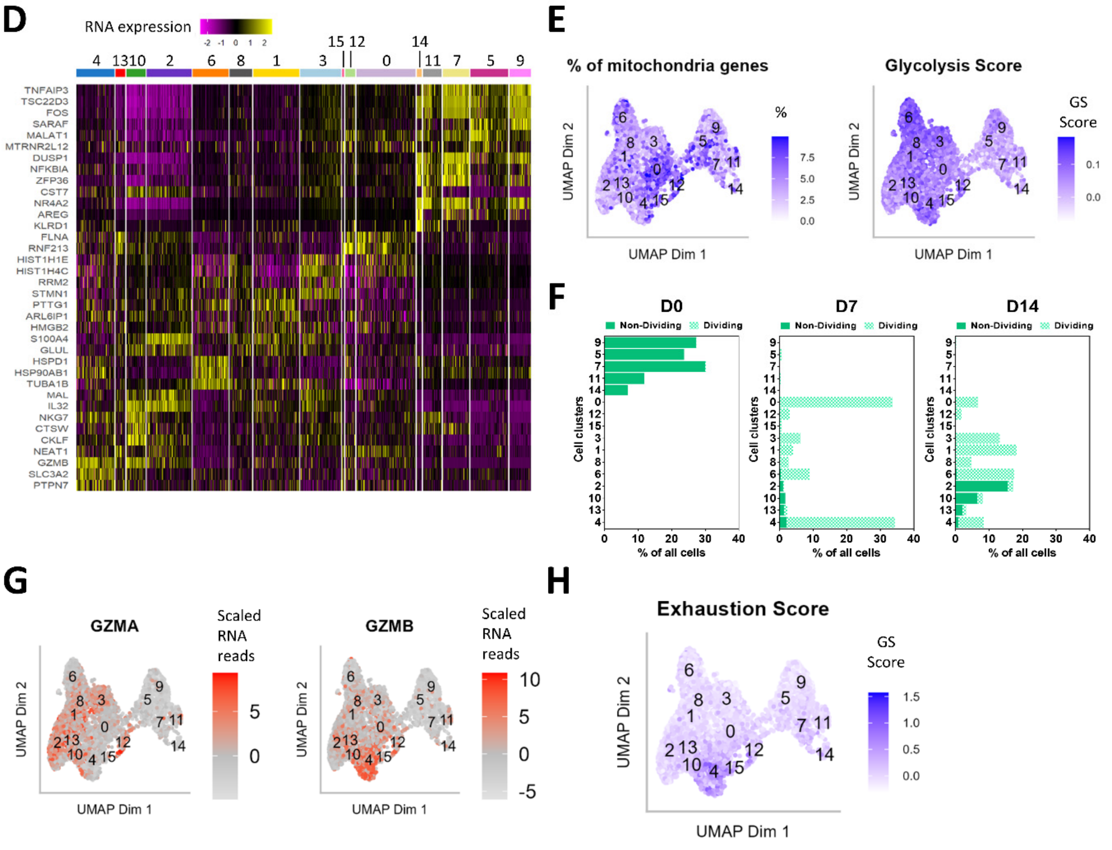

Fig S4. Cluster analysis of in vitro expanded REP$_{\gamma\delta}$.

(**A**) UMAP using scRNA-seq of D14-REP$_{\gamma\delta}$ from 3 different CB samples. (**B**) Percentage of all D14-REP$_{\gamma\delta}$ cells in each UMAP cell cluster that was significantly (Seurat label transfer prediction score of ≥0.5) mapped to reference γδ T cell types/origins published previously (19). (**C**) Scaled read counts of RNA (top panel) and cell surface protein (middle panel) of the indicated genes across cell clusters (top and middle panel). Representative FACS plots of cell surface expression of the indicated genes in total D14-REP$_{\gamma\delta}$ cells (bottom panel). (**D**) Heatmap showing the normalized RNA read counts of the top five most enriched gene in each of the sixteen cell clusters. (**E**) Percentage of mitochondria genes (left panel) and gene signature (GS) scoring for glycolysis (right panel) across cell clusters. (**F**) Cumulative percentage of cells in G1 and S/G2M of cell cycle within each cell cluster before (D0, left panel) and at D7 (middle

panel) or D14 (right panel) of REP culture. (**G**) Scaled RNA read counts of GZMA and GZMB across cell clusters. (**H**) GS score of cell exhaustion across cell clusters.

Supplementary Figure 5

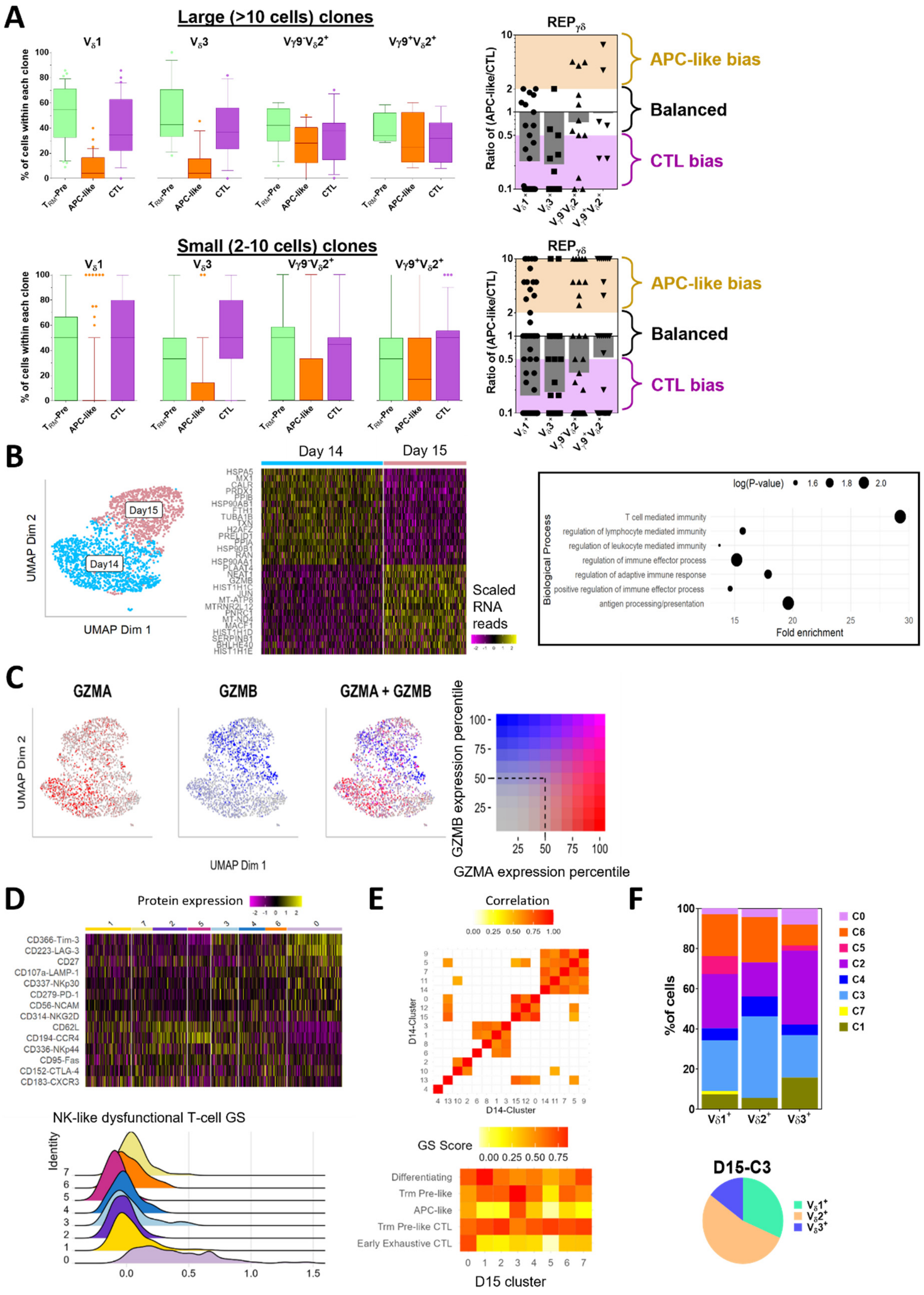

Fig S5. Differentiation trajectory of $CB_{\gamma\delta}$ in REP culture.

(**A**) Percentage of T_{RM}-precursor like, APC-like and CTL cells among large (>10 cells, top left panel) and small (2-10 cells, bottom left panel) $REP_{\gamma\delta}$ clones of the indicated clonotype. Line within box indicates the median, with box limits extend from the 25th to 75th percentiles of all clones. Whiskers are drawn down to the 10th percentile and up to the 90th. Ratio of APC-like cells to CTL within each $REP_{\gamma\delta}$ clone of the indicated clonotype (right panel). Bars show the geometric mean of all clones of the same clonotype. Clones with ratio of >2, 0.5-2 and <0.5 are categorised as being "APC-like bias", "Balanced" and "CTL bias" clones respectively. (**B**) Left panel: UMAP embedding of D14 & D15 cells, showing 2 distinct cell clusters separated by experimental conditions. Middle panel: Heatmap showing the normalized RNA expression of the top twenty most differentially expressed genes between D14 and D15 cells. Right panel: Plot of GO Slim Biological Processes that were significantly enriched in D15 cells compared to D14 cells. The size of the dots corresponds to the negative log-10 transformed FDR values. (**C**) Expression of GZMA/GMZB in D14 & D15 cells. Cells with GZMA (left panel) and GZMB (middle panel) RNA expression quantiles of ≥50% are indicated as red and blue respectively. Blended plot (GZMA+GZMB, right panel) shows the combined expression quantiles of both granzymes in individual cells. Cells with expression quantiles below 50% for both genes are shown in grey. (**D**) Top panel: Heatmap showing normalized expression of cell surface proteins in D15 cell clusters. Bottom panel: Ridge plot showing the distribution of T cell exhaustion scores in D15 cells within each cluster. (**E**) Correlation of marker gene scores in D14 cells. The top fifteen most upregulated genes in each D14 cell cluster were used to form sixteen gene sets (one per D14 cell cluster). Thereafter, correlations between the score of each gene sets were calculated using the Pearson correlation coefficient. For visualization, only correlations above 0.5 were colored (top panel). D14 cell clusters with highly correlated gene sets were then combined to form the following cell types: Differentiating (D14-C0/12/15), T_{RM}

precursors-like (D14-C1/3/8), APC-like (D14-C6), T_{RM} precursor-like CTL(D14-C2/10/13) and Early exhaustive CTL (D14-C4). The top fifteen marker genes of each D14 member cell cluster within individual cell type were collated to represent the gene signature (GS) for that cell type. Average GS scores of each cell type in individual D15 cell cluster were then calculated and shown as heatmap (bottom panel). (**F**) Top panel: Distribution of $V\delta1^+/2^+/3^+$ $\gamma\delta$ T cells with T_{RM} precursor-like expression profile across all D15 cell clusters. Bottom panel: Proportion of $V\delta1^+/2^+/3^+$ $\gamma\delta$ T cells with T_{RM} precursor-like expression profile within D15-C3.

Table S1: Percentage of T_N, T_{CM} and T_E cells within $V_\delta1^+$, $V_\delta2^+$, and $V_\delta1^-2^-$ γδ T cells in CB and PB samples

	% Within $V_\delta1^+$			% Within $V_\delta2^+$			% Within $V_\delta1^-2^-$		
**Sample**	**T_N, CD45RA$^+$CD27$^+$**	**T_{CM}, CD45RA$^-$CD27$^+$**	**T_E, CD45RA$^{+/-}$CD27$^-$**	**T_N, CD45RA$^+$CD27$^+$**	**T_{CM}, CD45RA$^-$CD27$^+$**	**T_E, CD45RA$^{+/-}$CD27$^-$**	**T_N, CD45RA$^+$CD27$^+$**	**T_{CM}, CD45RA$^-$CD27$^+$**	**T_E, CD45RA$^{+/-}$CD27$^-$**
**CB#1**	39.9	59.5	0.52	48.4	48.4	3.23	47.5	52.1	0.38
**CB#2**	60.8	39.2	0	64.3	21.4	14.3	64.3	28.6	7.14
**CB#3**	40.3	59.7	0	0	100	0	42.9	57.1	0
**CB#4**	28.1	65.6	6.34	50	37.5	12.5	44.2	51	4.76
**CB#5**	40	60	0	0	0	100	20	60	20
**CB#6**	24.7	67.1	8.24	20	60	20	38.5	53.8	7.69
**CB#7**	15.2	77	7.86	50	50	0	20.2	71.7	8.08
**CB#8**	14.1	71.8	14.06	0	0	0	0	0	0
**CB#9**	19.9	54.7	25.43	8.33	50	41.63	19.2	61	19.82
**CB#10**	10.9	63	26.02	0	0	0	18.7	56	25.3
**CB#11**	37.9	58.6	3.45	20	60	20	41.2	29.4	29.4
**CB#12**	33.3	66.7	0	0	0	0	37.5	12.5	50
**CB#13**	48.6	40.5	10.82	0	0	100	34.6	50	15.4
**CB#14**	22.2	66.7	11.1	0	0	0	14.3	61.9	23.82
**CB#15**	32.7	60	7.27	0	0	0	35.7	57.1	7.14
**CB#16**	15.6	55.9	28.45	15.4	61.5	23.09	18	45.6	36.44
**CB#17**	13.5	19.2	67.35	0	0	100	26.7	16.7	56.6
**CB#18**	31.2	62.5	6.25	0	75	25	17.9	82.1	0
**CB#19**	36.2	57.4	6.38	27.3	27.3	45.5	25	50	25
**Average**	*29.74*	*58.16*	*12.08*	*15.99*	*31.11*	*26.59*	*29.81*	*47.19*	*17.74*
**SD**	*13.39*	*13.09*	*16.07*	*21.70*	*31.77*	*35.51*	*15.04*	*20.80*	*16.72*
**SEM**	*3.07*	*3.00*	*3.69*	*4.98*	*7.29*	*8.15*	*3.45*	*4.77*	*3.84*
**PB#1**	14.3	0	85.7	0	23.9	76.1	12.5	0	87.5
**PB#2**	37.5	50	12.5	0	16.7	83.37	50	0	50
**PB#3**	0	66.7	33.3	2.08	6.94	91	33.3	0	66.7
**PB#4**	40.6	7.81	51.59	11.6	19.6	68.9	37.5	9.38	53.1
**PB#5**	19.3	15.8	64.87	2.04	4.76	93.2	8	16	76
**PB#6**	33.5	8.37	58.16	2.68	19.5	77.7	32	9.84	58.2
**PB#7**	62.3	17.4	20.3	1.82	10.2	88	41.9	27.1	31
**PB#8**	33.1	14	52.81	2	19.6	78.4	19.6	16.5	63.8
**PB#9**	14.2	12.6	73.2	0.85	2.56	96.6	18.4	26.5	55.1
**PB#10**	10.6	2.78	86.61	0.77	3.35	95.8	21.4	14.3	64.3
**PB#11**	68.4	26.3	5.26	0	20.4	79.7	15.6	37.5	46.92
**Average**	*30.35*	*20.16*	*49.48*	*2.17*	*13.41*	*84.43*	*26.38*	*14.28*	*59.33*
**SD**	*21.43*	*20.53*	*28.31*	*3.27*	*7.93*	*9.09*	*13.35*	*12.31*	*15.07*
**SEM**	*6.46*	*6.19*	*8.54*	*0.99*	*2.39*	*2.74*	*4.03*	*3.71*	*4.54*
**CB vs PB p value**	0.92	0.00	0.00	0.05	0.08	0.00	0.54	0.00	0.00

***Table S2: Calculated absolute number of* γδ T cells (x10^6) in REP culture**

	**PB15007**	**PB15059**	**PB13110**	**CB59260**	**CB61578**	**CB41351**
**Day 0**	0.015	0.004	0.008	0.021	0.024	0.013
**Day 14**	0.015	0.082	0.006	146.922	53.260	63.525

Table S3: Percentage of $CD25^+$, $CD69^+$ and $CD107a^+$ cells within $V_\delta 2^-$ and $V_\delta 2^+$ γδ T cell population before and after 14 days in REP culture

**Day 0**	**% within $V_\delta 2^-$**					**% within $V_\delta 2^+$**				
	**$CD69^-CD25^-$**	**$CD69^+CD25^-$**	**$CD69^+CD25^+$**	**$CD69^-CD25^+$**	**$CD107a^+$**	**$CD69^-CD25^-$**	**$CD69^+CD25^-$**	**$CD69^+CD25^+$**	**$CD69^-CD25^+$**	**$CD107a^+$**
**PB15007**	95.90	3.51	0.00	0.58	0.97	98.60	0.23	0.00	1.14	2.42
**PB15059**	95.10	4.88	0.00	0.00	1.16	97.70	1.52	0.00	0.76	0.72
**PB13110**	100.00	0.00	0.00	0.00	0.88	99.70	0.00	0.00	0.26	2.16
**CB59260**	99.40	0.37	0.00	0.23	0.74	98.30	0.34	0.00	1.36	1.21
**CB61578**	98.50	0.87	0.02	0.65	0.26	94.00	1.13	0.00	4.89	2.40
**CB41351**	98.60	1.04	0.00	0.35	0.44	87.90	0.00	3.03	9.09	4.55
**PB vs CB p-value**	0.30	0.24	0.37	0.40	0.03	0.16	0.88	0.37	0.12	0.44

**Day 14**	**% within $V_\delta 2^-$**					**% within $V_\delta 2^+$**				
	**$CD69^-CD25^-$**	**$CD69^+CD25^-$**	**$CD69^+CD25^+$**	**$CD69^-CD25^+$**	**$CD107a^+$**	**$CD69^-CD25^-$**	**$CD69^+CD25^-$**	**$CD69^+CD25^+$**	**$CD69^-CD25^+$**	**$CD107a^+$**
**PB15007**	7.14	10.70	22.90	59.30	91.00	1.29	3.02	23.30	72.40	92.30
**PB15059**	1.51	4.85	40.30	53.30	98.10	0.17	1.13	57.40	41.30	95.60
**PB13110**	11.40	35.20	39.80	13.60	71.30	0.00	0.00	100.00	0.00	100.00
**CB59260**	17.20	1.53	9.53	71.70	96.20	13.10	0.65	15.00	71.20	97.20
**CB61578**	19.90	2.02	9.60	69.10	94.80	7.12	1.99	11.70	79.20	96.70
**CB41351**	16.20	0.96	5.41	77.40	98.10	8.76	0.60	6.04	84.60	99.50
**PB vs CB p-value**	0.02	0.17	0.01	0.10	0.30	0.01	0.77	0.09	0.13	0.49
**PB D0 vs D14 p-value**	0.00	0.21	0.00	0.04	0.00	0.00	0.47	0.05	0.15	0.00
**CB D0 vs D14 p-value**	0.00	0.11	0.00	0.00	0.00	0.00	0.36	0.02	0.00	0.00

Table S4: Percentage of $V_\delta 1^+$, $V_\delta 2^+$, and $V_\delta 1^- 2^-$ cells within total γδ T cell population before and after 14 days in REP culture

$V_\delta 1^+$%	PB15007	PB15059	PB13110	CB59260	CB61578	CB41351
**Day 0**	15.0	11.2	17.8	55.2	59.9	47.1
**Day 14**	22.1	21.8	54.1	64.1	67.7	55.7

$V_\delta 2^+$%	PB15007	PB15059	PB13110	CB59260	CB61578	CB41351
**Day 0**	74.0	59.4	72.4	6.5	5.3	5.8
**Day 14**	60.7	63.0	6.5	5.6	5.2	3.7

$V_\delta 1^- 2^-$%	PB15007	PB15059	PB13110	CB59260	CB61578	CB41351
**Day 0**	9.9	27.2	8.2	38.0	34.6	46.8
**Day 14**	14.0	9.4	35.9	29.9	26.6	40.1

Table S5: Calculated absolute count of human REP $_{\gamma\delta}$ in AML-PDX mouse tissues 1 and 5 days post infusion.

	Day 1			
**Mouse ID**	**BM**	**LV**	**SPN**	**PB**
**AP2L**	38	456	1	633
**AP2N**	59	453	1	323
**AP3L**	36	329	17	288
**AP3N**	46	841	1	383
	**Day 5**			
**Mouse ID**	**BM**	**LV**	**SPN**	**PB**
**AP1L**	1,382	32,467	784	1,582
**AP1N**	1,078	23,073	35	1,035
**AP4L**	627	11,943	404	140
**AP4N**	782	10,295	366	2,687
**D1 vs D5 p-value**	0.001	0.011	0.043	0.127

BM: bone marrow; LV: liver; SPN: spleen; PB: peripheral blood

Calculated counts are expressed per i) 2 femurs+ 2 tibias in the BM; ii) 25% of whole mouse LV; iii) whole mouse SPN and iv) 1ml of PB

Table S6: Calculated absolute count of human AML and REP $_{\gamma\delta}$ in AML-PDX mouse bone marrow and spleen 6 days post infusion.

		BM		SPN	
**Mouse ID**	**Txpt AML cell dose**	**AML**	**REP$_{\gamma\delta}$**	**AML**	**REP$_{\gamma\delta}$**
**BH1R**	**5 x 10^5**	905,984	22,727	2,890	55,484
**BH1DR**		2,455,883	130,935	10,419	215,530
**BH2L**		2,333,609	124,559	6,698	91,358
**BH3R**		1,415,233	150,731	12,246	340,845
**BH4R**		913,762	6,397	3,018	947
**BH4DL**		1,744,941	92,336	8,929	15,368
**BH1N**	**3 x 10^6**	4,320,632	172,166	9,261	97,323
**BH2B**		7,160,879	100,038	29,734	124,472
**BH3L**		3,627,327	132,477	31,789	243,343
**BH4L**		2,556,715	112,826	29,736	131,145
**BH4DR**		2,766,756	21,259	14,725	4,647
**BH1B**	**2 x 10^7**	5,231,089	154,328	158,165	287,535
**BH2R**		8,070,741	227,001	287,551	160,873
**BH2N**		7,168,294	210,567	1,058,117	367,585
**BH4B**		5,298,918	123,655	88,585	82,042
**Spearman r**		0.60		0.64	
**95% confidence interval**		0.11 to 0.86		0.17 to 0.87	
**p-value**		0.02		0.01	

Calculated counts are expressed per i) 2 femurs+ 2 tibias in the BM and ii) whole mouse SPN

Table S7: Number and percentage of D0- & D14-$V_{\delta}1^{+}$/$V_{\delta}2^{+}$/$V_{\delta}1^{-}2^{-}$ clones in single cell cultures

Sample	D0-$V_{\delta}1^{+}$	D0-$V_{\delta}2^{+}$	D0-$V_{\delta}1^{-}2^{-}$	# D0 clones	D14LC*-$V_{\delta}1^{+}$	D14LC*-$V_{\delta}2^{+}$	D014LC*$V_{\delta}1^{-}2^{-}$	# D14LC*	%D14LC*-$V_{\delta}1^{+}$	%D14LC*-$V_{\delta}2^{+}$	%D014LC*$V_{\delta}1^{-}2^{-}$	Overall % D14LC*
CB39458	61	0	35	96	7	0	7	14	11.5%	NA	20.0%	14.6%
CB26666	53	3	38	94	8	0	6	14	15.1%	0.0%	15.8%	14.9%
CB40145	66	1	29	96	15	0	7	22	22.7%	0.0%	24.1%	22.9%
CB40259	54	5	37	96	14	2	8	24	25.9%	40.0%	21.6%	25.0%
**Total**	**234**	**9**	**139**	**382**	**44**	**2**	**28**	**74**	**18.8%**	**22.2%**	**20.1%**	**19.4%**

**LC: Large Clone, >500 cells*

Table S8: Expression profile of GZMK, IFNG and CCR7 in D0-C5/C9 and D0-C7/C11 respectively.

GZMK				
	**C5**	**C9**	**C7**	**C11**
**# Cells**	**183**	**212**	**233**	**92**
**GZMK+***	7	12	62	80
**GZMK-**	176	200	171	12
**Total GZMK+**	19		142	
**Total GZMK-**	376		183	
**% expression**	4.81%		43.69%	
**chi-square (C5/C9 vs C7/C11)**	155.27			
**p-value**	<0.01			
	**Wilcox test (C7/C11 vs C5/C9)**			
**Average logFC (C7/C11 vs C5/C9)**	4.3			
**p-value**	< 0.01			

IFNG				
	**C5**	**C9**	**C7**	**C11**
**# Cells**	**183**	**212**	**233**	**92**
**INFG+***	1	1	50	8
**INFG-**	182	211	183	84
**Total IFNG+**	2		58	
**Total IFNG-**	393		267	
**% expression**	0.51%		17.85%	
**chi-square (C5/C9 vs C7/C11)**	70.18			
**p-value**	<0.01			
	**Wilcox test (C7/C11 vs C5/C9)**			
**Average logFC (C7/C11 vs C5/C9)**	8.1			
**p-value**	< 0.01			

CCR7				
	**C5**	**C9**	**C7**	**C11**
**# Cells**	**183**	**212**	**233**	**92**
**CCR7+***	32	58	13	16
**CCR7-**	151	154	220	76
**Total CCR7+**	90		29	
**Total CCR7-**	305		296	
**% expression**	22.78%		8.92%	
**chi-square (C5/C9 vs C7/C11)**	24.83			
**p-value**	<0.01			
	**Wilcox test (C7/C11 vs C5/C9)**			
**Average logFC (C7/C11 vs C5/C9)**	NS			
**p-value**	NS			

* Scaled expression > 0

NS: Not significant

Table S9: Percentage of D0- & D14-$V_{\delta}1/V_{\delta}2/V_{\delta}3$ in the different cell clusters

	**Total cell #**	**C0**	**C1**	**C2**	**C3**	**C4**	**C5**	**C6**	**C7**	**C8**	**C9**	**C10**	**C11**	**C12**	**C13**	**C14**	**C15**
D0_47604-$V_{\delta}1$	408	0%	0%	0%	0%	0%	30.64%	0%	35.05%	0%	27.45%	0%	1.23%	0%	0%	5.64%	0%
D0_47604-$V_{\delta}2$	240	0%	0%	0%	0%	0%	11.67%	0%	22.08%	0%	21.67%	0%	35.00%	0%	0%	9.58%	0%
D0_47604-$V_{\delta}3$	123	0%	0%	0%	0%	0%	24.39%	0%	29.27%	0%	38.21%	0%	2.44%	0%	0%	5.69%	0%
	**Total cell #**	**C0**	**C1**	**C2**	**C3**	**C4**	**C5**	**C6**	**C7**	**C8**	**C9**	**C10**	**C11**	**C12**	**C13**	**C14**	**C15**
D14_47604-$V_{\delta}1$	507	3.94%	17.55%	21.89%	16.37%	7.50%	0.20%	11.64%	0.00%	5.13%	0.20%	8.88%	0.00%	1.38%	4.73%	0.39%	0.20%
D14_47604-$V_{\delta}2$	536	11.01%	20.15%	11.38%	11.01%	5.97%	0.00%	26.12%	0.19%	2.80%	0.19%	7.09%	0.00%	1.68%	2.43%	0.00%	0.00%
D14_47604-$V_{\delta}3$	316	4.43%	15.82%	19.94%	12.03%	13.92%	0.00%	12.34%	0.00%	7.59%	0.00%	8.86%	0.00%	2.53%	2.22%	0.00%	0.32%
	**Total cell #**	**C0**	**C1**	**C2**	**C3**	**C4**	**C5**	**C6**	**C7**	**C8**	**C9**	**C10**	**C11**	**C12**	**C13**	**C14**	**C15**
D14_41357-$V_{\delta}1$	547	25.41%	6.76%	13.53%	6.40%	11.15%	6.76%	0.0567	0.37%	7.31%	0.55%	4.02%	0.37%	3.47%	5.48%	0.00%	2.74%
D14_41357-$V_{\delta}2$	108	12.96%	10.19%	5.56%	8.33%	16.67%	2.78%	0.0926	0.00%	10.19%	0.00%	3.70%	4.63%	8.33%	6.48%	0.00%	0.93%
D14_41357-$V_{\delta}3$	103	17.48%	7.77%	3.88%	11.65%	11.65%	16.50%	0.0485	0.97%	5.83%	4.85%	5.83%	0.97%	3.88%	3.88%	0.00%	0.00%
	**Total cell #**	**C0**	**C1**	**C2**	**C3**	**C4**	**C5**	**C6**	**C7**	**C8**	**C9**	**C10**	**C11**	**C12**	**C13**	**C14**	**C15**
D14_41365-$V_{\delta}1$	997	18.76%	11.43%	9.23%	0.13139	5.92%	11.74%	3.71%	3.91%	7.22%	0.50%	2.61%	8.12%	2.81%	0.60%	0.10%	0.20%
D14_41365-$V_{\delta}2$	99	17.17%	9.09%	8.08%	0.08081	9.09%	13.13%	8.08%	2.02%	11.11%	1.01%	1.01%	8.08%	3.03%	1.01%	0.00%	0.00%
D14_41365-$V_{\delta}3$	288	15.63%	13.19%	16.32%	0.10764	7.29%	7.64%	6.25%	1.04%	7.99%	0.35%	7.99%	2.08%	2.43%	1.04%	0.00%	0.00%

Table S10: Differentially expressed genes between D0-C7 and D0-C11

Genes	pct.1_C7	pct.2_C11	(pct.1_C7 - pct.2_C11)	p_val	p_val_adj	avg_FC_(C7/C11)	avg_log2(1/FC)
ZNF683	0.717	0.109	0.608	5.72E-24	1.14E-20	6.963322383	-2.799775818
HSPB1	0.751	0.272	0.479	2.43E-15	4.85E-12	28.16587645	-4.81587646
LEF1	0.76	0.315	0.445	2.41E-16	4.81E-13	66.69851308	-6.059582694
CAMK4	0.657	0.217	0.44	2.84E-13	5.68E-10	28.63229489	-4.839571402
IKZF2	0.614	0.174	0.44	5.39E-13	1.08E-09	11.06499288	-3.467930618
SOX4	0.704	0.272	0.432	5.25E-13	1.05E-09	4.530604225	-2.179703469
CD52	0.618	0.207	0.411	3.72E-12	7.43E-09	8.094023282	-3.016856999
NR4A1	0.944	0.533	0.411	8.45E-22	1.69E-18	5.70776513	-2.51292597
RBL2	0.79	0.446	0.344	2.20E-09	4.40E-06	8.588663274	-3.10243361
ARIH1	0.708	0.38	0.328	4.87E-08	9.75E-05	17.6846603	-4.144426602
LIME1	0.674	0.348	0.326	1.69E-07	0.000337036	6.761763175	-2.757399488
TCF7	0.901	0.598	0.303	4.14E-13	8.28E-10	4.466970251	-2.159296647
TOB1	0.811	0.533	0.278	6.88E-07	0.001375182	2.867238087	-1.519661707
JUNB	0.957	0.685	0.272	1.46E-14	2.92E-11	5.168091005	-2.369631475
PPP1R15B	0.803	0.533	0.27	7.78E-08	0.000155561	2.452303588	-1.294137592
GATA3	0.781	0.511	0.27	2.48E-06	0.004963298	12.40644221	-3.633017549
RHOH	0.824	0.576	0.248	4.38E-08	8.76E-05	2.192260528	-1.132419258
RGS1	0.704	0.457	0.247	1.92E-05	0.038442492	3.735622877	-1.901348818
OGT	0.704	0.467	0.237	1.94E-06	0.00388891	27.8055422	-4.797300564
JUN	0.927	0.696	0.231	5.14E-10	1.03E-06	2.336932971	-1.224616355
SNHG15	0.717	0.489	0.228	4.29E-07	0.000858092	13.53310916	-3.758421423
STK17A	0.897	0.674	0.223	2.88E-09	5.77E-06	1.713972657	-0.777344094
KLF6	0.983	0.761	0.222	3.09E-22	6.18E-19	4.00746668	-2.002690525
CD7	0.725	0.511	0.214	1.41E-06	0.002814258	10.35269656	-3.37193469
MCL1	0.798	0.587	0.211	2.34E-06	0.004670361	7.078874864	-2.823520073
CD69	0.957	0.772	0.185	4.00E-07	0.000800786	2.056586976	-1.040252087
RIPOR2	0.738	0.554	0.184	6.59E-07	0.001318248	34.65154613	-5.114847821
IFITM1	0.979	0.837	0.142	7.80E-16	1.56E-12	3.492907922	-1.804428612
FOSB	0.953	0.815	0.138	7.62E-07	0.001524624	1.718537381	-0.781181233
PPP2R5C	0.82	0.707	0.113	3.39E-06	0.006773057	5.673138323	-2.50414704
PPP1R15A	0.97	0.87	0.1	2.50E-13	5.00E-10	2.408432644	-1.268094577
FOS	1	0.946	0.054	9.39E-11	1.88E-07	1.276335026	-0.352007072
DUSP1	0.996	0.946	0.05	1.74E-10	3.49E-07	1.478462111	-0.564097271
ZFP36L2	0.97	0.946	0.024	1.32E-07	0.00026446	1.715515305	-0.778641997
RPS27	0.966	0.989	-0.023	4.18E-14	8.37E-11	0.121814201	3.037245759
RPL34	0.953	0.978	-0.025	8.24E-07	0.001648043	0.287811364	1.796804539
RPS24	0.888	0.913	-0.025	9.83E-06	0.019666829	0.520963961	0.940744521
RPL30	0.944	0.989	-0.045	4.81E-14	9.61E-11	0.30323459	1.721493764
RPL13	0.936	0.989	-0.053	4.10E-14	8.20E-11	0.224935259	2.152418274
RPL32	0.893	0.967	-0.074	1.94E-07	0.000387488	0.379167847	1.399091463
RPL21	0.88	0.957	-0.077	9.77E-09	1.95E-05	0.273075212	1.872629734
RPL31	0.815	0.902	-0.087	8.48E-07	0.001696988	0.273251062	1.871700994
RPS12	0.845	0.957	-0.112	2.27E-05	0.045436974	0.434012974	1.204189926
RPL13A	0.858	0.978	-0.12	3.87E-08	7.74E-05	0.296658944	1.753122813
RPS15A	0.867	0.989	-0.122	5.97E-13	1.19E-09	0.320321583	1.642407085
RPL3	0.845	0.967	-0.122	1.69E-11	3.37E-08	0.329334772	1.602373253
CD5	0.588	0.717	-0.129	2.01E-07	0.000401463	0.100880283	3.309283864

RPL27A	0.824	0.957	-0.133	6.94E-11	1.39E-07	0.136767951	2.870197891
GZMM	0.717	0.87	-0.153	2.56E-06	0.005124462	0.326146824	1.616406514
CREM	0.558	0.717	-0.159	1.38E-06	0.002755873	0.153411174	2.704524523
RPS8	0.798	0.957	-0.159	5.00E-09	1.00E-05	0.341475187	1.550147343
RPS29	0.803	0.967	-0.164	2.81E-10	5.62E-07	0.127222128	2.974578474
RPL36A	0.73	0.913	-0.183	1.70E-07	0.000339245	0.438703867	1.188680671
LITAF	0.695	0.902	-0.207	1.09E-11	2.18E-08	0.177171878	2.496778469
RNF19A	0.562	0.772	-0.21	4.60E-12	9.19E-09	0.089708651	3.478609075
CD247	0.403	0.62	-0.217	1.93E-07	0.000385906	0.121280929	3.043575387
RPLP1	0.648	0.87	-0.222	8.90E-11	1.78E-07	0.243137938	2.040153073
FYN	0.549	0.772	-0.223	1.45E-07	0.000289206	0.077213433	3.695004326
NINJ1	0.502	0.739	-0.237	5.63E-06	0.011261596	0.175850932	2.507575117
DNAJB1	0.631	0.87	-0.239	9.61E-11	1.92E-07	0.264538035	1.918452927
NFKBIZ	0.446	0.707	-0.261	7.05E-07	0.001409051	0.183827952	2.443571939
RPS2	0.575	0.848	-0.273	1.25E-07	0.00024916	0.49397526	1.017489305
ARL4C	0.369	0.728	-0.359	6.91E-09	1.38E-05	0.407338469	1.295700027
MT2A	0.249	0.62	-0.371	2.19E-10	4.39E-07	0.177963534	2.490346439
CCL5	0.322	0.717	-0.395	5.90E-10	1.18E-06	0.446228781	1.164144528
SLC7A5	0.352	0.75	-0.398	1.40E-18	2.80E-15	0.059579762	4.069033836
KLRB1	0.352	0.761	-0.409	8.16E-22	1.63E-18	0.173930651	2.523415897
TNFRSF1B	0.176	0.717	-0.541	5.07E-22	1.01E-18	0.02050185	5.608102099
NKG7	0.172	0.717	-0.545	4.08E-22	8.15E-19	0.022857591	5.451182805
ZBTB16	0.082	0.63	-0.548	3.56E-25	7.11E-22	0.008980508	6.798987196
CST7	0.249	0.837	-0.588	5.23E-31	1.05E-27	0.006015799	7.377027814
GZMK	0.266	0.87	-0.604	2.05E-27	4.09E-24	0.15583544	2.681904724

Table S11: Distribution of CB $_{\gamma\delta}$ cell subsets across D0 cell clusters

			Cell # in each cluster						% cell in each cluster					
**TRDV**	**TRGV**	**TRGJ**	**9**	**5**	**7**	**11**	**14**	**Total**	**9**	**5**	**7**	**11**	**14**	**Total**
TRDV1	TRGV2		36	29	16	0	2	**83**	43.4%	34.9%	19.3%	0.0%	2.4%	**100%**
TRDV1	TRGV3		17	18	26	0	5	**66**	25.8%	27.3%	39.4%	0.0%	7.6%	**100%**
TRDV1	TRGV4		13	19	21	0	4	**57**	22.8%	33.3%	36.8%	0.0%	7.0%	**100%**
TRDV1	TRGV5		16	12	19	0	2	**49**	32.7%	24.5%	38.8%	0.0%	4.1%	**100%**
TRDV1	TRGV8		17	27	36	2	4	**86**	19.8%	31.4%	41.9%	2.3%	4.7%	**100%**
TRDV1	TRGV9		7	14	21	3	5	**50**	14.0%	28.0%	42.0%	6.0%	10.0%	**100%**
TRDV2	TRGV2		6	3	3	1	1	**14**	42.9%	21.4%	21.4%	7.1%	7.1%	**100%**
TRDV2	TRGV3		5	6	10	7	1	**29**	17.2%	20.7%	34.5%	24.1%	3.4%	**100%**
TRDV2	TRGV4		8	3	7	4	1	**23**	34.8%	13.0%	30.4%	17.4%	4.3%	**100%**
TRDV2	TRGV5		12	4	6	3	3	**28**	42.9%	14.3%	21.4%	10.7%	10.7%	**100%**
TRDV2	TRGV8		6	3	8	13	3	**33**	18.2%	9.1%	24.2%	39.4%	9.1%	**100%**
TRDV2	TRGV9	TRGJ1	1	2	10	6	6	**25**	4.0%	8.0%	40.0%	24.0%	24.0%	**100%**
TRDV2	TRGV9	TRGJP	6	3	7	39	7	**62**	9.7%	4.8%	11.3%	62.9%	11.3%	**100%**
TRDV2	TRGV9	TRGJP1	0	0	1	5	0	**6**	0.0%	0.0%	16.7%	83.3%	0.0%	**100%**
TRDV3	TRGV2		17	10	14	0	2	**43**	39.5%	23.3%	32.6%	0.0%	4.7%	**100%**
TRDV3	TRGV3		6	8	5	1	1	**21**	28.6%	38.1%	23.8%	4.8%	4.8%	**100%**
TRDV3	TRGV4		3	2	7	0	1	**13**	23.1%	15.4%	53.8%	0.0%	7.7%	**100%**
TRDV3	TRGV5		4	1	2	0	1	**8**	50.0%	12.5%	25.0%	0.0%	12.5%	**100%**
TRDV3	TRGV8		11	8	3	1	1	**24**	45.8%	33.3%	12.5%	4.2%	4.2%	**100%**
TRDV3	TRGV9		1	1	2	1	1	**6**	16.7%	16.7%	33.3%	16.7%	16.7%	**100%**

Table S12: Percentage of cell surface CD95$^+$, CD62L$^+$ and CD69$^+$ cells within total REP $_{\gamma\delta}$ analysed by flow cytometry

**Sample ID**	**CB12**	**CB13**	**CB59260**	**CB61578**	**CB41351**
**CD95**	99.7	93.6	ND	ND	ND
**CD62L**	ND	ND	87.2	74.2	87.5
**CD69**	ND	ND	11.3	11.7	6.4

ND: Not done

Table S13: Marker gene list of 16 REP $_{\gamma\delta}$ cell clusters

Genes	pct.1	pct.2	p_val	p_val_adj	cluster	avg_log2FC
ITGAD	0.943	0.011	1.18E-93	1.95E-89	14	1.993581405
KLRD1	1	0.095	5.37E-103	8.86E-99	14	2.927429792
AREG	0.906	0.072	6.41E-76	1.06E-71	14	3.287528789
NR4A2	1	0.191	4.98E-63	8.21E-59	14	3.745992671
IL7R	0.912	0.11	1.84E-157	3.03E-153	9	1.955818497
TCF7	0.925	0.15	1.43E-34	2.36E-30	14	1.633146034
NR4A2	0.921	0.163	5.32E-134	8.78E-130	9	2.162703701
ZNF331	0.93	0.185	3.95E-141	6.51E-137	9	2.18191676
ZNF331	0.943	0.213	1.90E-35	3.14E-31	14	2.356693446
DUSP2	1	0.289	1.57E-52	2.59E-48	14	3.066160665
RBM38	0.962	0.255	2.61E-33	4.30E-29	14	1.771617052
CMC1	0.962	0.265	6.50E-204	1.07E-199	14	2.762999107
TSC22D3	1	0.306	9.34E-33	1.54E-28	14	1.95455746
DDX3Y	0.925	0.24	1.01E-29	1.66E-25	14	1.48426328
PIK3IP1	0.916	0.235	1.25E-121	2.06E-117	9	1.554146568
RBM38	0.907	0.229	1.39E-137	2.29E-133	9	1.931363929
MSL2	0.906	0.242	3.12E-106	5.15E-102	14	2.165403305
TSC22D3	0.943	0.281	5.84E-137	9.63E-133	9	2.472908085
TSPYL2	0.962	0.3	9.22E-27	1.52E-22	14	1.480016998
DUSP2	0.916	0.265	3.05E-99	5.03E-95	9	1.636181748
CSRNP1	0.962	0.326	6.53E-33	1.08E-28	14	1.746197924
CDK1	0.946	0.318	1.74E-194	2.86E-190	3	1.422692328
DUSP1	1	0.392	6.70E-45	1.11E-40	14	2.547749608
FYN	0.962	0.36	7.92E-63	1.31E-58	14	1.779114601
GZMM	0.981	0.381	1.04E-36	1.72E-32	14	1.70730799
FOSB	0.917	0.318	1.22E-173	2.00E-169	7	2.164350182
SOX4	0.925	0.326	3.27E-33	5.40E-29	14	2.052094076
MAP3K8	0.981	0.387	1.66E-96	2.73E-92	14	2.005987967
DUSP1	0.96	0.37	2.80E-117	4.62E-113	9	1.925697297
ELL2	0.943	0.367	3.57E-49	5.88E-45	14	1.695116927
TNFAIP3	0.96	0.394	1.99E-160	3.29E-156	9	2.555009722
RGCC	0.906	0.341	2.94E-24	4.85E-20	14	1.772540791
DUSP1	0.921	0.365	2.09E-201	3.45E-197	7	2.791460068
DDIT4	0.943	0.388	1.81E-30	2.99E-26	14	1.631726831
YPEL5	0.981	0.427	4.47E-26	7.38E-22	14	1.46094535
CCNA2	0.944	0.395	1.42E-128	2.35E-124	3	1.020325869
TNFAIP3	0.962	0.416	2.19E-17	3.61E-13	14	0.752900278
REL	0.943	0.398	7.15E-32	1.18E-27	14	1.57203605
YPEL5	0.938	0.407	4.79E-156	7.90E-152	9	2.023304725
CBLB	1	0.471	1.16E-07	0.001915221	15	0.750814306
IFITM2	1	0.472	1.09E-41	1.80E-37	14	2.160520446
TNFAIP3	0.906	0.391	4.78E-176	7.89E-172	7	2.678892434
HSPD1	0.997	0.808	2.35E-230	3.88E-226	6	1.44998749
CXCR4	0.956	0.442	6.41E-111	1.06E-106	9	1.751297301
ADAM19	1	0.486	2.07E-07	0.003409563	15	0.733148448

CENPM	0.911	0.398	1.99E-132	3.29E-128	3	1.034067563
HELZ	1	0.491	3.65E-09	6.01E-05	15	0.945765009
PIM3	0.962	0.453	9.13E-30	1.51E-25	14	1.57497042
NCAPH	0.904	0.396	9.63E-114	1.59E-109	3	0.920598983
UBE2C	0.911	0.415	1.70E-123	2.81E-119	3	1.27243714
KNL1	0.909	0.413	6.40E-06	0.105499627	15	0.689569572
DNAJB1	0.962	0.469	6.22E-39	1.03E-34	14	1.622706861
RPS4Y1	1	0.512	2.19E-19	3.61E-15	14	1.142131196
NFKB1	0.981	0.496	1.80E-30	2.97E-26	14	1.643556602
NUSAP1	0.967	0.486	6.80E-187	1.12E-182	3	1.510689614
ASPM	0.908	0.428	1.53E-88	2.53E-84	3	0.723818873
IFITM2	0.928	0.449	1.10E-127	1.81E-123	7	1.767448238
SETX	1	0.529	1.55E-06	0.025579907	15	0.630200883
HSP90AB1	1	0.939	3.32E-172	5.48E-168	6	1.449130228
GAS5	0.969	0.501	8.09E-154	1.33E-149	9	1.602038677
LINC01578	0.981	0.523	3.67E-18	6.06E-14	14	0.984929476
GADD45B	0.962	0.506	7.59E-38	1.25E-33	14	1.955660805
C1QBP	0.947	0.542	1.67E-201	2.75E-197	6	1.372651381
FABP5	0.955	0.485	2.53E-158	4.18E-154	6	1.357039815
BTG1	1	0.551	3.43E-33	5.66E-29	14	1.901941343
TOP2A	0.927	0.481	4.22E-124	6.95E-120	3	1.32201639
BTG1	0.978	0.534	4.57E-160	7.54E-156	9	2.331996352
RPS4Y1	0.934	0.496	3.41E-108	5.63E-104	9	1.581065194
PCLAF	0.941	0.508	3.80E-140	6.27E-136	3	1.142525301
NFKB1	0.912	0.481	2.22E-102	3.66E-98	9	1.588270784
LEPROTL1	0.947	0.522	8.17E-145	1.35E-140	9	1.589226005
GADD45B	0.912	0.49	8.12E-91	1.34E-86	9	1.468761344
LINC01578	0.93	0.508	2.69E-92	4.44E-88	9	1.295985999
TFRC	1	0.583	4.85E-07	0.00799702	15	0.916617609
FAM111A	0.904	0.487	1.32E-97	2.18E-93	3	0.851681773
SMC2	0.906	0.491	2.33E-88	3.85E-84	3	0.837398352
FOS	0.993	0.581	1.05E-226	1.73E-222	7	3.012462673
KIF22	0.913	0.503	3.36E-105	5.54E-101	3	0.92007371
TUBA1B	0.997	0.96	7.42E-125	1.22E-120	6	1.343743345
MKNK2	0.906	0.5	9.88E-33	1.63E-28	14	1.239893406
NME1	0.963	0.448	3.97E-198	6.55E-194	6	1.338220298
CASP8	0.909	0.507	1.08E-05	0.178275238	15	0.818876017
FOS	1	0.602	4.06E-41	6.70E-37	14	2.528256372
BTG1	0.928	0.532	7.96E-153	1.31E-148	7	2.256214608
PNRC1	0.986	0.592	6.68E-168	1.10E-163	7	2.037221828
KLF2	1	0.607	1.01E-15	1.67E-11	14	1.116102743
CKAP5	0.909	0.518	0.000126607	1	15	0.708809598
PNRC1	1	0.611	1.37E-46	2.26E-42	14	2.254599742
PRDX3	0.931	0.603	7.55E-161	1.25E-156	6	1.190215041
ATAD2	0.906	0.517	6.26E-75	1.03E-70	3	0.774323681
SRA1	0.955	0.567	0.000116966	1	15	0.590360508
FOS	0.974	0.587	2.49E-145	4.11E-141	9	2.368210061

RUNX3	1	0.613	1.27E-05	0.209140991	15	0.627033218
PNRC1	0.978	0.597	1.03E-145	1.69E-141	9	1.978152309
HIST1H1B	0.967	0.586	6.25E-142	1.03E-137	3	1.585056919
TK1	0.918	0.538	1.89E-108	3.12E-104	3	1.014430233
ZFP36L2	0.987	0.613	1.36E-160	2.25E-156	9	2.273840656
GNPTAB	0.909	0.535	5.84E-05	0.963939279	15	0.696668197
SRPRA	1	0.626	1.21E-05	0.199357944	15	0.640611242
PPP1R15A	0.993	0.62	4.47E-211	7.38E-207	7	2.14782605
ZFP36L2	1	0.627	2.80E-24	4.62E-20	14	1.544787437
TPX2	0.913	0.54	2.67E-73	4.40E-69	3	0.805511566
TUBB	1	0.965	4.50E-116	7.42E-112	6	1.185427352
HSPE1	0.973	0.641	8.33E-152	1.37E-147	6	1.172639043
HSPA4	0.909	0.541	0.000498501	1	15	0.594821141
ITGA4	0.906	0.542	2.43E-20	4.01E-16	14	1.146659051
BTG2	1	0.637	1.25E-37	2.06E-33	14	1.626147816
USP1	0.908	0.551	8.57E-80	1.41E-75	3	0.75896399
RFC1	0.909	0.552	0.000358928	1	15	0.67302425
IL2RB	0.962	0.609	9.78E-35	1.61E-30	14	1.332270567
H2AFZ	0.997	0.97	2.03E-126	3.35E-122	6	1.1667422
ADGRE5	0.962	0.61	1.91E-26	3.16E-22	14	1.377995227
ZFP36L2	0.96	0.61	2.31E-192	3.80E-188	7	2.505899475
KLF2	0.943	0.594	1.65E-81	2.72E-77	9	1.509705224
BTG2	0.968	0.62	8.51E-113	1.40E-108	7	1.357113352
PPP1R15A	0.969	0.625	1.13E-132	1.86E-128	9	1.784931519
NPM1	0.997	0.974	5.29E-141	8.72E-137	6	1.154136192
HIST1H1B	0.955	0.62	3.53E-05	0.58252946	15	0.953578055
CENPF	0.941	0.606	1.98E-79	3.26E-75	3	0.942360517
RAN	1	0.97	2.11E-172	3.48E-168	6	1.15323178
TUBB4B	0.995	0.809	4.18E-126	6.89E-122	6	1.15306953
FOS	0.909	0.578	8.43E-50	1.39E-45	5	0.712562792
TIMP1	0.91	0.58	4.85E-113	8.00E-109	2	1.246639466
GAPDH	1	1	8.25E-136	1.36E-131	6	1.142082565
TPI1	1	0.991	2.37E-155	3.91E-151	6	1.131895248
HUWE1	0.909	0.581	0.000993555	1	15	0.728351905
BHLHE40	0.924	0.597	6.78E-34	1.12E-29	10	0.76383726
FOS	0.918	0.592	6.00E-28	9.90E-24	11	0.814493367
PPP1R15A	0.962	0.639	3.01E-21	4.96E-17	14	1.401228962
TXN	1	0.968	2.44E-129	4.03E-125	6	1.081789467
YBX1	1	0.981	2.87E-141	4.73E-137	6	1.067370211
GPRIN3	1	0.678	5.09E-07	0.008393086	15	0.993243697
RANBP1	0.973	0.644	6.36E-148	1.05E-143	6	1.05655081
PRNP	0.909	0.589	0.00231027	1	15	0.588752858
PGAM1	0.997	0.922	3.95E-148	6.52E-144	6	1.02326935
RPLP0	1	1	2.58E-164	4.26E-160	6	1.019219458
HSP90AA1	1	1	1.18E-139	1.95E-135	6	1.011538343
PTPN22	0.955	0.639	0.000199776	1	15	0.727830122
KLF2	0.906	0.592	1.44E-113	2.37E-109	7	1.831570087

RRM2	0.972	0.66	1.76E-176	2.90E-172	3	1.621039519
CD69	0.982	0.671	3.77E-214	6.22E-210	7	2.190221102
RSRP1	0.943	0.632	1.82E-22	3.00E-18	14	1.170044673
UBE2S	0.941	0.689	1.37E-71	2.26E-67	6	1.007029178
RRM2	0.971	0.664	6.32E-87	1.04E-82	6	1.00210017
TYMS	0.984	0.728	4.19E-95	6.91E-91	6	0.989944745
ADGRE5	0.903	0.596	1.59E-119	2.61E-115	7	1.392083465
HSPA8	1	1	1.30E-133	2.15E-129	6	0.981035365
RPS2	1	0.996	1.49E-81	2.46E-77	6	0.975637203
CD81	1	0.694	1.63E-23	2.68E-19	14	1.131740708
CCND2	0.981	0.858	5.73E-80	9.45E-76	6	0.947415548
ANTXR2	0.904	0.601	1.02E-31	1.68E-27	12	1.043942081
CHD1	0.925	0.623	1.35E-13	2.22E-09	14	0.86539913
ANTXR2	0.909	0.607	0.001864553	1	15	0.594954446
IDI1	0.943	0.642	5.80E-31	9.57E-27	14	1.267970567
IFITM1	0.996	0.697	2.21E-127	3.65E-123	7	1.689415794
SYNE2	0.933	0.637	6.49E-31	1.07E-26	12	1.266741599
IFITM1	0.996	0.701	1.63E-58	2.69E-54	9	1.011483667
DPP4	0.909	0.614	0.0032802	1	15	0.621986893
DUT	0.989	0.791	5.17E-101	8.53E-97	6	0.933593653
CCL5	0.954	0.662	8.22E-39	1.36E-34	11	1.272521173
MIF	1	0.991	5.29E-129	8.73E-125	6	0.925991931
BTG2	0.912	0.627	5.20E-35	8.58E-31	9	0.762025064
C12orf57	0.943	0.659	4.67E-20	7.70E-16	14	1.13730889
JUND	1	0.718	1.33E-21	2.19E-17	14	1.240835487
PPIA	1	1	7.06E-134	1.16E-129	6	0.920257854
PDE4D	0.903	0.625	4.81E-57	7.93E-53	9	0.95336008
CCT6A	0.992	0.872	4.68E-140	7.71E-136	6	0.919752615
ZFP36L1	0.903	0.626	1.61E-50	2.66E-46	7	0.952014201
PPP1R15A	0.908	0.631	5.64E-23	9.31E-19	11	0.751310638
PRKDC	0.933	0.657	1.19E-40	1.96E-36	12	1.155677102
PRELID1	0.997	0.883	6.55E-145	1.08E-140	6	0.913143229
NIBAN1	1	0.725	0.000533324	1	15	0.672644903
H1FX	0.943	0.668	5.99E-10	9.88E-06	14	0.934186763
C12orf57	0.921	0.648	2.05E-68	3.38E-64	9	1.053960558
LDHA	0.997	0.996	2.34E-109	3.87E-105	6	0.902215376
IFITM1	0.981	0.712	4.36E-25	7.18E-21	14	1.659423956
GPRIN3	0.942	0.674	5.05E-43	8.32E-39	12	1.07581227
SMAP2	0.981	0.714	1.50E-22	2.47E-18	14	1.133932111
ZFP36L1	0.906	0.64	5.16E-16	8.50E-12	14	1.263762911
CD69	0.943	0.677	2.30E-51	3.79E-47	9	1.025001438
ATP2B4	0.99	0.725	3.17E-41	5.22E-37	12	1.257211027
SMAP2	0.969	0.704	2.33E-82	3.84E-78	9	1.118255811
LITAF	1	0.739	3.35E-21	5.53E-17	14	1.085842819
IFITM1	0.954	0.693	2.61E-62	4.31E-58	5	1.047660331
HSPE1	0.925	0.665	3.08E-19	5.08E-15	14	1.205296865
IFITM1	0.964	0.704	5.45E-28	9.00E-24	11	0.753448652

SIVA1	0.936	0.676	1.97E-61	3.26E-57	3	0.617670152
STIP1	0.947	0.657	3.99E-113	6.59E-109	6	0.895642433
CCT5	0.973	0.753	3.25E-122	5.36E-118	6	0.895429511
TUBA1C	0.941	0.534	2.29E-80	3.78E-76	6	0.886273178
PEBP1	0.955	0.675	3.93E-109	6.48E-105	6	0.873201252
TYMS	0.979	0.726	3.37E-167	5.55E-163	3	1.408543302
CCT2	0.955	0.712	2.61E-108	4.31E-104	6	0.869371494
CCNL1	0.989	0.739	1.55E-125	2.55E-121	7	1.341312703
CCNL1	1	0.751	1.95E-24	3.22E-20	14	1.243973679
KDSR	0.939	0.69	1.12E-45	1.84E-41	10	0.868330848
TUBA1A	0.934	0.687	1.06E-69	1.74E-65	9	1.125503179
PRKDC	0.909	0.662	0.001484443	1	15	0.673830515
TRERF1	1	0.756	7.04E-05	1	15	0.906905633
PCLAF	0.963	0.511	3.14E-94	5.18E-90	6	0.863395032
ATP5MC3	1	0.968	7.41E-122	1.22E-117	6	0.850053326
NME2	1	0.987	3.03E-115	5.00E-111	6	0.825471018
SMC3	0.908	0.666	2.79E-61	4.60E-57	3	0.693310757
TGFB1	1	0.759	4.39E-15	7.24E-11	14	0.838856641
NIBAN1	0.962	0.721	3.86E-21	6.37E-17	12	0.791820562
PRF1	0.949	0.71	2.67E-24	4.41E-20	10	0.903328973
ITGB1	0.952	0.715	2.45E-27	4.04E-23	12	1.223103724
CLU	0.936	0.7	1.02E-56	1.69E-52	2	0.752333054
RHOH	0.939	0.703	1.22E-100	2.01E-96	7	1.195961502
CCNL1	0.978	0.742	3.36E-67	5.55E-63	9	1.015565919
HNRNPL	0.962	0.726	2.85E-12	4.70E-08	14	0.73155486
AL133415.1	0.909	0.675	0.000277846	1	15	0.815812881
RHOH	0.938	0.706	2.22E-65	3.66E-61	9	1.023091958
VAMP2	0.906	0.675	6.80E-12	1.12E-07	14	0.76559633
NDUFAB1	0.963	0.688	1.87E-113	3.09E-109	6	0.820558322
EIF4A1	1	0.993	6.53E-118	1.08E-113	6	0.820554903
CUTA	0.962	0.735	2.58E-24	4.25E-20	14	1.007015555
ISG20	0.947	0.72	2.62E-78	4.32E-74	2	0.833900827
NUDT1	0.958	0.731	2.16E-60	3.56E-56	3	0.634098743
EIF4B	0.982	0.756	2.57E-96	4.23E-92	9	1.087494817
GLG1	0.952	0.726	9.44E-22	1.56E-17	12	0.754547602
NHP2	0.952	0.563	2.89E-99	4.77E-95	6	0.81826935
ATP2B4	0.955	0.73	0.002132133	1	15	0.590289206
PRDX1	0.997	0.935	4.31E-80	7.10E-76	6	0.813074459
SMC1A	0.939	0.718	5.74E-48	9.47E-44	3	0.621940156
JUND	0.93	0.71	4.00E-40	6.60E-36	9	0.887567898
CACYBP	0.968	0.69	5.19E-99	8.57E-95	6	0.804334983
MACF1	1	0.78	0.000272232	1	15	0.600551723
SNHG29	0.991	0.772	7.11E-167	1.17E-162	9	1.543196318
TSPO	0.925	0.708	9.70E-72	1.60E-67	2	0.782511591
CCNL1	0.952	0.735	1.66E-44	2.74E-40	5	0.628289013
JUND	0.924	0.708	1.30E-85	2.14E-81	7	1.253428168
DYNC1H1	0.952	0.736	9.55E-24	1.58E-19	12	0.831703815

CCNL1	0.959	0.745	1.71E-23	2.82E-19	11	0.62786989
DSTN	0.908	0.695	2.22E-59	3.67E-55	2	0.701706037
GBP5	0.949	0.736	9.56E-24	1.58E-19	10	0.69793057
SMC1A	0.921	0.71	8.08E-78	1.33E-73	0	0.729468443
PTPN7	0.962	0.751	8.29E-118	1.37E-113	4	0.954166829
RHOH	0.925	0.715	1.51E-08	0.000249819	14	0.712936156
MYO1F	0.926	0.716	1.35E-65	2.23E-61	0	0.633451432
TRERF1	0.962	0.752	5.20E-20	8.57E-16	12	0.790101092
CHD4	0.952	0.742	6.17E-17	1.02E-12	12	0.670496007
GLUL	0.951	0.742	7.92E-64	1.31E-59	2	0.705070603
TRERF1	0.961	0.752	8.36E-14	1.38E-09	13	0.658147667
SMC1A	0.942	0.734	2.06E-20	3.40E-16	12	0.839507381
IKZF2	0.913	0.706	8.05E-20	1.33E-15	12	0.81181495
NOP53	0.982	0.775	9.82E-70	1.62E-65	9	0.88335623
LMNB1	0.932	0.726	4.38E-60	7.23E-56	3	0.681456223
MCM7	0.955	0.584	4.06E-76	6.70E-72	6	0.789336033
ATP2B4	0.931	0.726	1.23E-10	2.03E-06	13	0.617711442
CLU	0.919	0.715	2.06E-23	3.40E-19	10	0.886356927
PRMT2	0.906	0.703	1.84E-08	0.000303981	14	0.602604036
H2AFX	0.969	0.767	1.13E-149	1.87E-145	3	1.184901644
SNHG29	0.981	0.781	1.03E-20	1.69E-16	14	1.09349569
RPS26	1	1	2.96E-106	4.88E-102	6	0.787777334
IKZF2	0.909	0.71	0.000329237	1	15	0.71598597
TNFRSF1B	0.99	0.791	1.76E-29	2.91E-25	12	0.902842956
SMAP2	0.903	0.705	2.05E-40	3.38E-36	7	0.740298645
HIST1H1C	1	0.802	1.15E-07	0.0018957	15	1.191579335
ENO1	1	0.998	1.09E-80	1.79E-76	6	0.78368819
HLA-DQA2	0.909	0.712	7.34E-21	1.21E-16	10	0.721154362
CCT7	0.965	0.722	2.26E-103	3.72E-99	6	0.780247315
EIF4B	0.962	0.765	6.44E-16	1.06E-11	14	0.869165647
EIF3E	0.943	0.748	2.35E-82	3.88E-78	9	0.954661342
SLC38A1	0.971	0.776	1.95E-32	3.21E-28	12	0.920250838
LITAF	0.924	0.73	2.37E-49	3.90E-45	7	0.911942195
HIST1H1D	1	0.808	2.86E-06	0.047127406	15	0.936459419
EIF5A	0.995	0.931	3.36E-88	5.55E-84	6	0.779691143
PRF1	0.904	0.716	4.07E-17	6.72E-13	12	1.456528981
PGD	0.92	0.612	5.08E-69	8.38E-65	6	0.769410079
EIF4B	0.942	0.756	4.20E-73	6.93E-69	7	0.904459042
NOP53	0.96	0.774	5.98E-49	9.87E-45	7	0.707104023
MACF1	0.962	0.777	1.04E-51	1.71E-47	12	1.367492794
MAL	0.936	0.754	3.80E-98	6.27E-94	2	1.19598262
CST7	0.99	0.808	2.84E-44	4.68E-40	11	0.939301868
EIF5	0.981	0.799	1.43E-14	2.35E-10	14	0.783988005
CYCS	0.943	0.762	3.43E-10	5.66E-06	14	0.698474025
SQOR	0.915	0.596	1.26E-69	2.09E-65	6	0.765845219
HNRNPAB	0.96	0.739	2.17E-71	3.57E-67	6	0.744043501
DUT	0.969	0.79	2.43E-112	4.01E-108	3	1.07731581

CIRBP	0.943	0.764	4.53E-86	7.47E-82	9	1.031800571
DIAPH1	0.981	0.802	3.07E-31	5.06E-27	12	0.879014795
ISG15	0.902	0.724	8.19E-15	1.35E-10	13	0.800500139
TGFB1	0.928	0.751	1.90E-48	3.14E-44	7	0.797744506
SNRPG	0.984	0.891	8.85E-100	1.46E-95	6	0.742307532
CCT4	0.973	0.797	6.24E-94	1.03E-89	6	0.742258345
CIRBP	0.943	0.77	7.69E-30	1.27E-25	14	1.181312179
SNHG29	0.946	0.773	5.43E-41	8.95E-37	7	0.703344939
LMNB1	0.913	0.741	3.18E-14	5.25E-10	12	0.678959615
CST7	0.97	0.798	2.39E-54	3.94E-50	2	0.649756473
BRD2	1	0.829	1.18E-20	1.94E-16	14	0.95253915
NUDT1	0.9	0.729	4.20E-71	6.93E-67	0	0.671430895
RESF1	0.942	0.771	1.58E-15	2.60E-11	12	0.790884206
KLF6	1	0.83	3.16E-210	5.21E-206	7	2.010886247
CIRBP	0.932	0.762	8.40E-48	1.39E-43	7	0.729511847
TMPO	0.965	0.796	8.41E-92	1.39E-87	3	0.882185841
TNFRSF1B	0.962	0.794	1.52E-22	2.51E-18	14	1.136038187
SLC25A5	0.995	0.977	4.51E-81	7.44E-77	6	0.741831066
SPTBN1	0.981	0.816	2.03E-33	3.35E-29	12	1.055534524
PDIA6	0.962	0.797	1.93E-39	3.18E-35	12	1.090248491
HIST1H1D	0.958	0.794	6.01E-99	9.91E-95	3	1.074722221
TMPO	0.971	0.808	9.57E-20	1.58E-15	12	0.828964414
CD63	0.939	0.776	8.52E-30	1.41E-25	10	0.711250325
ZC3HAV1	0.912	0.751	8.09E-32	1.33E-27	9	0.654550382
KLF6	1	0.839	4.27E-08	0.000705008	14	0.654498929
CST7	0.97	0.809	7.30E-35	1.20E-30	10	0.904072086
ESYT1	0.952	0.792	2.48E-37	4.09E-33	12	0.969196994
HLA-DMA	0.971	0.811	1.28E-23	2.10E-19	12	0.957513956
STK17A	1	0.841	3.60E-22	5.94E-18	14	1.095572523
JUN	1	0.842	8.39E-10	1.38E-05	14	1.000226166
MKI67	0.991	0.833	1.07E-92	1.76E-88	3	0.998443101
HNRNPA0	0.943	0.785	8.93E-17	1.47E-12	14	0.906659467
ELF1	0.938	0.78	3.00E-48	4.95E-44	9	0.815033138
TUBB4B	0.967	0.81	2.07E-75	3.41E-71	3	0.822515453
ZFP36	0.996	0.84	3.20E-222	5.27E-218	7	2.18923452
DOCK8	0.971	0.815	3.91E-32	6.44E-28	12	0.91677843
KLF6	0.99	0.834	1.36E-25	2.24E-21	11	0.661870566
KTN1	0.933	0.778	3.44E-17	5.68E-13	12	0.636082138
KLF6	0.987	0.833	2.22E-60	3.66E-56	9	1.133390877
TAGLN2	1	0.98	1.30E-73	2.14E-69	6	0.740595459
KLF6	0.98	0.827	2.08E-43	3.44E-39	5	0.723635698
CD164	0.942	0.789	1.83E-32	3.02E-28	12	0.850691048
ZFP36	1	0.848	1.68E-53	2.78E-49	14	2.250070708
JUN	0.986	0.835	9.79E-170	1.61E-165	7	2.069980709
JUN	0.987	0.837	2.50E-59	4.12E-55	9	1.208192459
HNRNPA0	0.93	0.78	9.05E-55	1.49E-50	9	0.817374191
BCLAF1	0.943	0.793	6.20E-08	0.001022841	14	0.614260803

BRD2	0.971	0.822	1.09E-72	1.79E-68	7	0.916861736
BCLAF1	0.935	0.786	1.66E-54	2.74E-50	7	0.777507998
ZFP36	0.987	0.842	4.35E-74	7.17E-70	9	1.28162141
STK17A	0.978	0.834	3.92E-164	6.47E-160	7	1.427038075
SRSF5	1	0.857	6.49E-50	1.07E-45	14	1.582519961
HIST1H1C	0.932	0.79	1.38E-92	2.28E-88	3	1.141467194
CD37	0.971	0.829	9.99E-38	1.65E-33	7	0.630823742
TCP1	0.939	0.628	1.41E-86	2.33E-82	6	0.737068302
BATF	0.98	0.839	1.60E-30	2.64E-26	10	0.762526303
ELF1	0.921	0.78	5.29E-41	8.72E-37	7	0.722940604
ELF1	0.916	0.776	3.46E-43	5.71E-39	5	0.657404222
SRSF5	0.989	0.85	4.38E-115	7.23E-111	7	1.153977922
RNF149	0.981	0.842	1.77E-28	2.92E-24	12	0.907316801
CD96	0.962	0.823	1.06E-21	1.75E-17	12	0.836839231
CD37	0.969	0.831	7.38E-35	1.22E-30	9	0.618814586
RPN2	0.942	0.804	2.81E-23	4.63E-19	12	0.702443245
EIF3L	0.982	0.845	1.38E-100	2.28E-96	9	1.052201238
MKI67	0.981	0.845	7.66E-19	1.26E-14	12	1.354890913
SHISA5	0.949	0.813	1.54E-59	2.54E-55	2	0.586702954
MKI67	0.964	0.83	9.62E-115	1.59E-110	0	1.192135404
RNF149	0.964	0.831	7.94E-88	1.31E-83	2	0.761336877
PNISR	0.952	0.819	1.20E-55	1.98E-51	5	0.694637141
CD82	0.977	0.846	6.33E-93	1.04E-88	4	0.921763497
EIF5	0.925	0.794	9.38E-49	1.55E-44	9	0.745614414
EIF3L	0.981	0.85	1.22E-12	2.00E-08	14	0.708166985
HIST1H1E	1	0.87	2.75E-11	4.53E-07	15	1.67500372
SRSF5	0.982	0.852	1.25E-59	2.06E-55	9	0.887609914
HLA-DQB1	0.97	0.841	1.64E-52	2.70E-48	10	1.04196253
HCST	1	0.871	5.14E-42	8.48E-38	11	0.81359838
CDC25B	0.969	0.84	1.22E-63	2.01E-59	3	0.743176662
ETS1	1	0.871	8.04E-11	1.33E-06	14	0.731059791
GPX4	0.919	0.79	4.28E-59	7.07E-55	4	0.716555172
BATF	0.971	0.842	2.05E-12	3.39E-08	13	0.683423395
SLC2A3	0.989	0.861	1.59E-76	2.62E-72	7	1.080190471
BATF	0.962	0.834	6.70E-44	1.10E-39	4	0.69340491
HIST1H1E	0.986	0.859	2.29E-119	3.77E-115	3	1.575834062
PIM2	0.919	0.792	1.50E-47	2.47E-43	2	0.706694238
SLC2A3	0.987	0.862	6.56E-118	1.08E-113	9	1.443568703
ZFP36	0.969	0.844	1.44E-35	2.37E-31	11	1.102627542
HMGB2	0.988	0.864	1.38E-115	2.28E-111	3	1.11960647
TNFSF10	0.99	0.866	1.99E-20	3.29E-16	12	0.914711852
SRRM2	1	0.876	2.86E-13	4.71E-09	14	0.760105662
ETS1	0.991	0.867	4.32E-41	7.13E-37	9	0.716059755
NFKBIA	0.996	0.873	1.65E-160	2.73E-156	7	1.938485591
RPL23	1	0.877	2.14E-134	3.52E-130	9	1.249651611
ACTG1	1	1	8.34E-77	1.38E-72	6	0.736981215
PPA1	0.929	0.806	5.66E-25	9.34E-21	10	0.751963484

CD82	0.975	0.852	1.28E-30	2.11E-26	10	0.709331044
DDX3X	0.932	0.809	1.45E-35	2.40E-31	7	0.61399837
PSAP	0.962	0.84	3.06E-17	5.04E-13	12	0.631019744
TAPBP	1	0.878	0.00015812	1	15	0.588550386
NFKBIA	1	0.879	4.38E-43	7.22E-39	14	2.009140316
SNRPE	0.931	0.563	2.30E-84	3.80E-80	6	0.73408186
MRPS15	0.96	0.638	8.89E-85	1.47E-80	6	0.728988117
HIST1H1D	0.913	0.793	3.71E-64	6.12E-60	0	0.807087405
FURIN	1	0.88	4.20E-21	6.93E-17	13	0.727681429
HCST	0.989	0.869	7.79E-40	1.28E-35	7	0.670046142
RPL23	1	0.881	4.12E-15	6.80E-11	14	0.923647099
HLA-DMA	0.929	0.81	1.89E-28	3.11E-24	10	0.686124071
HLA-DQB1	0.962	0.843	2.23E-09	3.69E-05	12	0.618663948
HOPX	0.985	0.867	1.70E-101	2.81E-97	2	1.01987832
BCLAF1	0.907	0.789	2.80E-38	4.61E-34	9	0.686187621
NFKBIA	0.991	0.874	7.77E-51	1.28E-46	9	1.01104901
PRKCH	0.943	0.826	6.57E-07	0.010841554	14	0.58746846
MCL1	0.986	0.87	5.34E-96	8.81E-92	7	1.125920709
UCP2	0.979	0.863	9.76E-86	1.61E-81	2	0.827814379
STK17A	0.952	0.837	2.46E-30	4.05E-26	9	0.695895086
TMPO	0.911	0.796	3.03E-53	5.00E-49	0	0.596265264
STMN1	0.991	0.877	2.53E-200	4.17E-196	3	1.593785952
LBR	0.99	0.876	2.60E-63	4.29E-59	12	1.273930762
NCL	0.992	0.963	2.49E-69	4.11E-65	6	0.727241274
TAPBP	0.99	0.876	3.92E-26	6.46E-22	12	0.833118647
ARGLU1	1	0.886	1.21E-11	2.00E-07	14	0.711198798
IQGAP2	0.99	0.88	5.23E-32	8.63E-28	12	0.94138823
DDX17	0.981	0.871	6.38E-10	1.05E-05	14	0.649106142
RPL23	0.986	0.876	3.37E-41	5.56E-37	7	0.648131652
CANX	0.962	0.853	1.00E-60	1.65E-56	12	1.266089268
ETS1	0.975	0.866	5.52E-51	9.10E-47	7	0.802179784
ARGLU1	0.989	0.88	3.07E-52	5.07E-48	7	0.737786728
SURF4	0.904	0.796	1.04E-13	1.72E-09	12	0.633390567
MKI67	0.955	0.848	0.000208031	1	15	0.788518681
CD82	0.962	0.855	7.91E-16	1.30E-11	12	0.742215101
LCP2	0.975	0.868	1.05E-35	1.73E-31	10	0.692925284
TPST2	0.983	0.877	1.84E-124	3.03E-120	2	0.949582178
NOSIP	0.925	0.82	3.82E-63	6.31E-59	9	0.908968203
TNFSF10	0.97	0.865	8.78E-35	1.45E-30	10	0.868061008
SRSF5	0.959	0.854	1.16E-21	1.92E-17	11	0.602293234
SMC4	0.986	0.883	1.11E-77	1.83E-73	3	0.742423537
PA2G4	0.992	0.937	6.65E-84	1.10E-79	6	0.720704526
ACTR2	0.99	0.887	3.15E-17	5.19E-13	12	0.648569964
AKAP13	0.99	0.888	9.65E-28	1.59E-23	12	0.882341784
STMN1	0.979	0.877	3.05E-67	5.03E-63	1	0.71920557
GZMA	0.989	0.888	2.12E-78	3.49E-74	2	1.028864113
GYPC	0.993	0.893	1.43E-38	2.36E-34	7	0.639708822

XBP1	0.975	0.876	1.18E-50	1.94E-46	10	0.924055867
HCST	0.97	0.872	2.39E-36	3.94E-32	10	0.841946138
DDOST	0.904	0.806	4.79E-17	7.89E-13	12	0.651283219
LGALS3	0.991	0.893	5.56E-49	9.17E-45	2	0.592448547
PNISR	0.925	0.829	1.77E-08	0.00029126	14	0.632960669
ARGLU1	0.975	0.879	2.80E-53	4.61E-49	5	0.689235068
CCT3	0.963	0.809	1.01E-86	1.67E-82	6	0.720287767
IFI16	1	0.904	3.88E-13	6.40E-09	12	0.58726721
SARAF	0.996	0.901	9.64E-213	1.59E-208	9	1.990519917
FUS	0.996	0.901	2.10E-45	3.46E-41	9	0.74118904
SPN	1	0.905	2.85E-05	0.46993506	15	0.637410096
SELL	0.923	0.829	8.07E-19	1.33E-14	12	1.015895699
EIF3G	0.981	0.887	2.75E-23	4.53E-19	14	0.959059739
GYPC	0.987	0.894	2.60E-70	4.29E-66	9	0.904529195
RBM39	1	0.907	2.41E-56	3.97E-52	9	0.821244819
PDCD5	0.955	0.66	3.71E-79	6.12E-75	6	0.71721788
HIST1H1E	0.95	0.858	7.50E-51	1.24E-46	0	0.942899786
ACTN4	1	0.908	0.000568287	1	15	0.665052853
STAT1	1	0.909	0.001139718	1	15	0.621235864
SRSF7	1	0.91	3.76E-16	6.20E-12	14	0.876367417
RBM39	1	0.911	7.52E-25	1.24E-20	14	1.09466398
SARAF	0.989	0.9	1.71E-58	2.82E-54	7	0.922110208
COMMD6	0.962	0.873	1.15E-07	0.001901617	14	0.599782602
HIST1H4C	0.993	0.905	1.89E-229	3.12E-225	3	1.965069991
RBM39	0.992	0.904	1.25E-53	2.07E-49	5	0.679625389
SLC2A3	0.949	0.861	1.85E-28	3.05E-24	5	0.606371186
CTSW	0.949	0.862	5.64E-37	9.30E-33	10	1.187266031
SH3BP1	0.955	0.868	3.49E-60	5.76E-56	2	0.593023289
SPN	0.99	0.904	1.90E-39	3.14E-35	12	0.970766314
SELPLG	0.99	0.904	2.64E-26	4.36E-22	12	0.869494795
TNFSF10	0.955	0.869	0.0002472	1	15	0.736555008
ARGLU1	0.969	0.883	1.33E-38	2.20E-34	9	0.674800758
HMGB2	0.952	0.867	1.02E-50	1.69E-46	1	0.813575437
ATP6V0C	1	0.916	1.66E-17	2.74E-13	14	0.800570921
GBP2	0.954	0.87	4.47E-19	7.37E-15	10	0.59952104
TNFSF10	0.945	0.862	1.14E-65	1.88E-61	4	0.911793006
STAT1	0.99	0.907	2.44E-17	4.03E-13	12	0.800466722
NFKBIA	0.959	0.877	1.18E-25	1.95E-21	11	1.120404349
RBM39	0.989	0.907	2.89E-115	4.77E-111	7	1.11804049
DDX17	0.952	0.87	2.34E-19	3.86E-15	12	0.686989233
BZW1	0.981	0.9	4.65E-08	0.000766212	14	0.592643205
RPN1	0.913	0.834	3.09E-16	5.09E-12	12	0.610367576
CALM2	0.984	0.905	5.51E-94	9.09E-90	3	0.750569671
HNRNPDL	0.996	0.918	1.07E-65	1.77E-61	9	0.841234276
FUS	0.978	0.901	3.28E-49	5.41E-45	7	0.733435658
BST2	1	0.923	9.88E-35	1.63E-30	10	0.658362405
FUS	0.981	0.905	5.09E-24	8.40E-20	14	1.076335633

CCND2	0.942	0.866	1.68E-15	2.77E-11	12	0.890001719
SRSF7	0.982	0.906	1.47E-58	2.42E-54	7	0.878784488
SFPQ	1	0.924	7.95E-12	1.31E-07	14	0.755497518
BANF1	0.968	0.661	9.58E-84	1.58E-79	6	0.706028765
RPS17	1	0.924	7.50E-80	1.24E-75	6	0.705874725
GZMA	0.97	0.895	3.46E-22	5.70E-18	10	0.87763608
GBP2	0.942	0.867	1.25E-33	2.06E-29	4	0.603597802
MCL1	0.947	0.873	1.96E-26	3.23E-22	9	0.681433156
HOPX	0.949	0.876	1.34E-32	2.21E-28	10	0.962842955
NUCB1	0.981	0.908	3.24E-25	5.34E-21	12	0.733531169
SF1	0.968	0.895	1.99E-38	3.28E-34	7	0.613693408
SH3BP5	0.985	0.913	1.78E-66	2.94E-62	2	0.643867732
NUCKS1	0.973	0.901	3.11E-68	5.13E-64	0	0.642909236
SARAF	0.974	0.902	6.02E-18	9.92E-14	11	0.64133669
RPS17	0.996	0.926	1.25E-58	2.06E-54	9	0.797428566
C12orf75	0.979	0.909	8.13E-82	1.34E-77	1	0.741524244
TUBA4A	0.995	0.963	4.19E-64	6.91E-60	6	0.699740455
HSPA5	1	0.931	1.16E-17	1.91E-13	14	1.07887783
SF1	0.965	0.896	5.51E-46	9.10E-42	9	0.726511085
DNAJA1	0.942	0.873	4.06E-41	6.70E-37	7	0.653970905
H2AFV	0.972	0.904	1.26E-86	2.07E-82	3	0.788133345
CARHSP1	0.993	0.926	3.08E-83	5.09E-79	3	0.731210904
LAT	0.945	0.878	4.15E-52	6.84E-48	2	0.66512345
SARAF	0.965	0.9	5.46E-71	9.01E-67	5	1.22911605
CLEC2D	0.99	0.925	4.70E-30	7.75E-26	12	0.900916039
GZMA	0.962	0.897	5.52E-10	9.11E-06	12	0.860124962
CKLF	0.998	0.933	1.27E-63	2.09E-59	2	0.706571553
CARHSP1	0.989	0.924	3.01E-74	4.97E-70	0	0.607882499
SRSF9	0.912	0.715	2.31E-60	3.82E-56	6	0.699644088
HLA-DPB1	0.99	0.926	7.32E-10	1.21E-05	12	0.592645112
GZMB	1	0.938	0.000279645	1	15	0.956239893
RPS20	1	0.938	2.30E-47	3.79E-43	7	0.682575547
MANF	0.904	0.57	2.63E-63	4.34E-59	6	0.698052372
TLN1	0.971	0.909	1.46E-18	2.40E-14	13	0.662673406
MRPL37	0.904	0.551	2.23E-72	3.68E-68	6	0.694318676
TAP1	1	0.939	4.60E-21	7.58E-17	12	0.709503893
HSPA5	0.99	0.93	4.48E-16	7.39E-12	12	1.033836136
PPP1R18	0.981	0.921	2.79E-68	4.60E-64	2	0.605389424
PTGES3	0.963	0.86	4.82E-81	7.95E-77	6	0.692640442
HIST1H4C	0.963	0.905	1.04E-64	1.72E-60	0	1.002443138
CKLF	0.995	0.938	2.61E-58	4.31E-54	10	1.101974806
RPS20	0.996	0.939	8.26E-99	1.36E-94	9	1.086801706
IER2	1	0.943	7.67E-80	1.27E-75	7	1.084008099
JAK1	1	0.944	3.57E-08	0.000589132	14	0.596662467
DEK	0.993	0.937	5.65E-76	9.32E-72	3	0.677414199
BAX	0.939	0.883	1.10E-44	1.82E-40	3	0.63708842
SF3B1	0.981	0.925	1.33E-16	2.19E-12	12	0.636653558

HLA-DPA1	1	0.945	1.57E-16	2.58E-12	12	0.882015529
HMGA1	0.91	0.731	1.39E-52	2.30E-48	6	0.686179503
HLA-DPB1	0.98	0.925	3.96E-25	6.53E-21	10	0.659268589
IER2	1	0.946	8.25E-20	1.36E-15	14	1.215598084
NEAT1	0.994	0.941	8.13E-90	1.34E-85	0	0.910097318
FUT7	1	0.947	1.45E-17	2.39E-13	12	0.819991113
GZMB	0.987	0.934	3.98E-126	6.56E-122	4	2.068704059
CTSD	0.959	0.906	1.65E-34	2.71E-30	10	0.773069504
PGAM1	0.977	0.924	1.72E-37	2.84E-33	4	0.618818229
RPL27A	1	0.948	3.22E-104	5.31E-100	9	1.14989192
NEAT1	1	0.948	3.49E-05	0.575642202	15	0.837944721
RPL27A	1	0.949	5.51E-30	9.09E-26	11	0.771672168
JPT1	0.996	0.945	2.00E-82	3.30E-78	1	0.753488402
HLA-DPA1	0.995	0.944	1.98E-27	3.27E-23	10	0.703140076
PDIA3	1	0.95	1.07E-22	1.76E-18	12	0.882343682
RPL27A	1	0.95	2.05E-13	3.39E-09	14	0.815685403
CRIP1	0.984	0.935	2.05E-67	3.39E-63	3	0.824730378
GZMB	0.985	0.936	3.86E-22	6.37E-18	10	0.798490469
ITGB7	0.981	0.932	1.08E-11	1.78E-07	12	0.590719818
SLC9A3R1	0.998	0.95	1.28E-65	2.11E-61	3	0.643484842
P4HB	0.971	0.923	2.06E-16	3.40E-12	12	0.699854706
CD53	0.987	0.941	5.94E-70	9.81E-66	2	0.620140348
CD53	0.99	0.945	9.62E-37	1.59E-32	12	1.005123442
RPL27A	0.993	0.948	2.79E-40	4.60E-36	7	0.690885694
IQGAP1	1	0.955	0.000509469	1	15	0.600719866
CTSD	0.952	0.907	2.76E-14	4.56E-10	12	0.616032929
RPL38	1	0.957	2.05E-126	3.38E-122	9	1.170994774
CD52	1	0.957	1.75E-122	2.89E-118	2	1.02459412
CRIP1	0.977	0.934	7.68E-51	1.27E-46	0	0.62933334
RPL38	1	0.959	2.67E-11	4.41E-07	14	0.681337639
ARL6IP1	0.933	0.893	1.38E-67	2.27E-63	1	1.042452969
RNASEK	0.995	0.955	1.58E-45	2.60E-41	10	0.782687972
SIVA1	0.934	0.679	2.96E-64	4.89E-60	6	0.684707717
COX5A	1	0.97	1.70E-91	2.80E-87	6	0.682444825
SEC61B	0.98	0.94	1.25E-27	2.05E-23	10	0.587801384
HNRNPF	0.995	0.956	9.43E-66	1.56E-61	3	0.620340453
RPL38	0.996	0.957	3.62E-40	5.97E-36	7	0.603675389
FLNA	0.998	0.96	6.87E-151	1.13E-146	0	0.994287436
ACTB	1	1	2.26E-53	3.72E-49	6	0.682378444
BST2	0.962	0.925	1.05E-17	1.74E-13	12	0.718791352
TUBA1B	0.995	0.959	1.47E-121	2.42E-117	3	1.323285903
IQGAP1	0.99	0.954	6.23E-62	1.03E-57	12	1.211107018
ARL6IP1	0.933	0.897	9.07E-12	1.50E-07	12	0.799589048
TUBA4A	0.998	0.962	8.84E-68	1.46E-63	3	0.691249272
EIF3F	0.996	0.96	9.47E-38	1.56E-33	9	0.589094654
VDAC1	0.955	0.726	4.70E-75	7.76E-71	6	0.681528603
AHNAK	1	0.965	1.17E-26	1.93E-22	12	1.078145817

FLNA	1	0.965	2.56E-33	4.22E-29	13	1.072192319
AHNAK	1	0.965	2.48E-06	0.040871912	15	1.035061923
LTB	1	0.965	1.66E-24	2.75E-20	10	0.927301358
AHNAK	1	0.965	6.32E-17	1.04E-12	13	0.886417014
ALOX5AP	1	0.965	1.37E-89	2.26E-85	2	0.826708667
LY6E	0.998	0.963	1.39E-92	2.29E-88	2	0.808159057
RPS29	1	0.966	1.45E-101	2.38E-97	9	1.07066168
NEAT1	0.981	0.947	1.84E-10	3.03E-06	12	0.95882431
AHNAK	0.995	0.961	1.77E-76	2.93E-72	0	0.759173068
RPS29	1	0.966	7.42E-31	1.22E-26	11	0.711068235
LTB	1	0.966	5.72E-09	9.43E-05	13	0.655624112
ALOX5AP	1	0.967	2.77E-60	4.56E-56	10	1.077989702
NEAT1	0.98	0.947	1.10E-16	1.82E-12	13	0.986202352
RPS29	1	0.967	9.15E-16	1.51E-11	14	0.838051029
EIF3A	0.981	0.948	7.99E-24	1.32E-19	12	0.763193353
PHB	0.934	0.616	3.40E-74	5.61E-70	6	0.678976564
HLA-DRA	0.995	0.963	3.61E-33	5.96E-29	10	0.853337273
SSRP1	0.96	0.656	4.45E-71	7.33E-67	6	0.678250611
FTL	1	1	4.11E-53	6.77E-49	6	0.673836833
CTSC	1	0.969	3.30E-44	5.44E-40	10	0.885862534
JUNB	1	0.97	9.57E-179	1.58E-174	7	1.821400101
TUBB	0.995	0.965	1.35E-159	2.23E-155	3	1.488761126
SNRPD1	0.965	0.765	1.02E-75	1.68E-71	6	0.66867433
CTSC	1	0.97	1.58E-22	2.61E-18	12	0.935713318
SRGN	1	0.97	1.14E-46	1.88E-42	7	0.764203888
STOML2	0.923	0.601	1.37E-70	2.25E-66	6	0.666861202
JUNB	1	0.971	3.07E-87	5.06E-83	9	1.300913351
SRGN	1	0.971	2.16E-44	3.57E-40	10	0.901038761
TOMM22	0.902	0.562	4.70E-67	7.75E-63	6	0.664699738
SRGN	1	0.971	1.27E-23	2.10E-19	11	0.687002425
JUNB	1	0.972	5.46E-23	9.01E-19	14	1.337479523
ARL6IP5	0.995	0.967	2.71E-51	4.48E-47	10	0.874923783
EEF1B2	1	0.973	7.55E-119	1.25E-114	9	1.215040643
CCT8	0.949	0.783	8.75E-70	1.44E-65	6	0.660560244
HLA-DRB5	1	0.973	8.05E-23	1.33E-18	12	1.145235622
NKG7	1	0.973	8.10E-38	1.34E-33	11	0.981223711
SRGN	0.997	0.97	1.60E-69	2.64E-65	4	0.86276115
RAD21	1	0.973	2.44E-14	4.03E-10	12	0.673331682
H2AFZ	0.996	0.969	1.97E-62	3.25E-58	1	0.659423093
RPS29	0.993	0.966	4.80E-47	7.92E-43	7	0.656896253
HLA-DRA	0.99	0.964	1.71E-19	2.82E-15	12	1.004586928
TMBIM6	1	0.974	2.28E-28	3.77E-24	12	0.978696617
RPL36A	1	0.975	1.71E-181	2.83E-177	9	1.539550932
LTB	0.989	0.964	3.92E-46	6.47E-42	2	0.928343051
ITGB2	1	0.975	4.65E-22	7.67E-18	12	0.804298009
RPL36A	1	0.975	8.94E-42	1.47E-37	7	0.678817629
FLNA	0.99	0.965	7.13E-13	1.18E-08	12	0.634278144

MT-ATP8	0.984	0.959	1.65E-70	2.72E-66	0	0.61908551
HLA-DRB1	1	0.976	4.15E-36	6.85E-32	10	0.889247307
RPL36A	1	0.976	1.78E-15	2.93E-11	14	0.85258279
S100A11	1	0.976	5.86E-74	9.67E-70	1	0.755388215
PTMA	1	1	2.83E-69	4.67E-65	6	0.660338157
H2AFZ	0.993	0.97	5.15E-116	8.50E-112	3	1.050401399
IL32	1	0.977	1.39E-71	2.29E-67	2	1.013535753
MT-ND6	1	0.977	2.12E-05	0.349168444	15	0.940082336
HLA-DRB1	1	0.977	1.22E-16	2.01E-12	12	0.867436426
MT-ND6	1	0.977	2.04E-17	3.37E-13	12	0.732161122
CD3G	0.99	0.967	3.73E-13	6.16E-09	12	0.620112013
ATP5F1B	0.995	0.983	5.64E-86	9.31E-82	6	0.651583625
TUBA1B	0.983	0.96	6.67E-45	1.10E-40	1	0.603710487
NKG7	0.995	0.973	3.58E-41	5.90E-37	10	1.198499372
HSP90B1	1	0.978	1.59E-18	2.63E-14	12	0.931064986
HLA-DRB5	0.995	0.973	5.26E-32	8.67E-28	10	0.885361139
ALOX5AP	0.99	0.968	1.62E-12	2.68E-08	12	0.733596238
TMBIM6	0.995	0.973	4.03E-33	6.66E-29	10	0.670064939
RPL31	1	0.979	5.71E-129	9.42E-125	9	1.198420772
EZR	0.993	0.972	1.37E-71	2.26E-67	7	0.981607644
NPM1	0.996	0.975	4.74E-55	7.81E-51	9	0.831546254
RPL31	1	0.979	3.90E-16	6.43E-12	14	0.81032003
ACTR3	1	0.979	1.26E-17	2.08E-13	12	0.630665284
LGALS1	1	0.979	9.23E-10	1.52E-05	13	0.619663865
S100A11	0.998	0.977	1.37E-54	2.25E-50	2	0.610308744
BIRC5	0.949	0.499	1.28E-77	2.10E-73	6	0.650887055
PFDN5	1	0.98	3.30E-46	5.44E-42	7	0.587674379
EZR	0.991	0.972	2.19E-78	3.61E-74	9	1.079873131
HNRNPD	0.989	0.868	1.54E-67	2.53E-63	6	0.64955145
PFDN5	1	0.981	6.81E-17	1.12E-12	14	0.794261757
GPI	0.995	0.936	8.86E-73	1.46E-68	6	0.635746374
HMGN2	1	0.983	1.83E-105	3.02E-101	3	1.016355586
RNF213	1	0.983	1.12E-129	1.85E-125	0	0.970241541
RPL31	0.996	0.979	3.85E-47	6.35E-43	7	0.628505594
MSN	1	0.983	1.96E-17	3.24E-13	12	0.589367699
LGALS1	0.994	0.978	5.97E-51	9.85E-47	1	0.71855764
MSN	1	0.984	0.000181636	1	15	0.593492129
RNF213	1	0.985	2.42E-51	3.99E-47	12	1.547969167
RNF213	1	0.985	2.86E-08	0.000471515	15	1.220405018
RPS11	1	0.985	1.46E-97	2.40E-93	9	0.975373358
RNF213	1	0.985	1.14E-20	1.89E-16	13	0.950671992
DDX5	1	0.985	2.96E-50	4.88E-46	9	0.739643823
RPS11	1	0.985	2.75E-14	4.54E-10	14	0.737993062
PFDN5	0.996	0.981	5.51E-42	9.09E-38	9	0.60742964
DDX5	1	0.986	2.65E-30	4.37E-26	14	1.146724882
SLC25A6	1	0.986	4.10E-76	6.76E-72	9	0.847843963
EMP3	1	0.986	4.20E-90	6.94E-86	2	0.718853355

ARL6IP5	0.981	0.968	1.56E-27	2.57E-23	12	0.945771558
MT-ND6	0.99	0.977	9.64E-22	1.59E-17	13	0.868050452
CHCHD2	1	0.999	1.42E-84	2.35E-80	6	0.634674743
HMGN2	0.996	0.983	6.60E-80	1.09E-75	1	0.788666812
MT-ND6	0.989	0.976	1.45E-65	2.39E-61	0	0.697292549
PRRC2C	0.99	0.978	2.90E-24	4.78E-20	12	0.748458223
NKG7	0.985	0.973	6.87E-31	1.13E-26	4	0.717683306
HNRNPU	1	0.988	4.40E-16	7.25E-12	12	0.66110689
PFN1	1	1	3.15E-61	5.19E-57	6	0.633622103
BTF3	1	0.988	1.74E-45	2.86E-41	9	0.646726798
RPL22	1	0.989	2.81E-130	4.63E-126	9	1.179940672
DDX5	0.996	0.985	1.01E-81	1.66E-77	7	0.923762884
S100A4	1	0.99	3.81E-104	6.29E-100	2	1.132174998
HLA-E	1	0.99	4.34E-93	7.16E-89	7	0.966793053
HLA-E	1	0.99	7.82E-17	1.29E-12	14	0.909870295
HLA-E	1	0.99	3.73E-51	6.16E-47	9	0.779205909
RPL22	1	0.99	1.25E-14	2.06E-10	14	0.751566012
DDX5	0.995	0.985	3.11E-47	5.13E-43	5	0.683397382
S100A4	1	0.99	1.90E-21	3.14E-17	8	0.647472474
ITGB2	0.985	0.975	2.29E-22	3.77E-18	10	0.586860407
RPL7	1	0.991	1.33E-179	2.20E-175	9	1.429546697
FKBP1A	0.96	0.818	2.85E-56	4.69E-52	6	0.626964649
CALR	1	0.991	9.04E-19	1.49E-14	12	0.995158911
MT-ATP8	0.971	0.962	1.58E-31	2.61E-27	12	0.993175587
LMNB1	0.955	0.727	1.10E-51	1.82E-47	6	0.626105395
S100A6	1	0.991	2.31E-71	3.81E-67	2	0.809545622
RPL7	1	0.991	3.19E-49	5.27E-45	7	0.660200333
S100A6	1	0.991	5.30E-48	8.74E-44	1	0.602475489
S100A4	0.998	0.99	9.23E-63	1.52E-58	1	0.839409914
MYH9	1	0.992	2.83E-22	4.67E-18	12	0.802031806
RPL7	1	0.992	4.62E-11	7.62E-07	14	0.664677425
MYH9	1	0.992	0.000164631	1	15	0.650071001
COTL1	0.985	0.977	3.43E-40	5.66E-36	1	0.647358259
S100A4	0.998	0.99	3.38E-35	5.57E-31	3	0.616026567
MT-ND3	1	0.993	2.33E-27	3.84E-23	12	0.852750082
MICOS10	0.939	0.68	2.27E-62	3.74E-58	6	0.625056361
LIMD2	1	0.993	5.18E-65	8.54E-61	2	0.616429081
MT-ND4	0.998	0.991	2.91E-65	4.80E-61	0	0.59783753
RPL37A	1	0.994	1.17E-123	1.93E-119	9	1.164885564
IL32	0.985	0.979	2.29E-26	3.77E-22	10	0.875781778
IL2RG	1	0.994	1.58E-22	2.60E-18	12	0.840457973
RPL37A	1	0.994	3.90E-14	6.44E-10	14	0.736327292
IL2RG	1	0.994	1.88E-39	3.10E-35	10	0.70571256
CALR	0.997	0.991	5.46E-55	9.01E-51	6	0.619185137
RPL27	1	0.995	5.86E-99	9.66E-95	9	0.971804876
CD3D	1	0.995	8.10E-44	1.34E-39	10	0.717869986
IL2RG	0.998	0.993	1.52E-86	2.51E-82	2	0.702315486

RPL27	1	0.995	3.64E-12	6.00E-08	14	0.675058607
PABPC1	1	0.996	7.47E-38	1.23E-33	14	1.50249012
PABPC1	1	0.996	5.18E-141	8.55E-137	9	1.417195947
PABPC1	1	0.996	1.24E-110	2.05E-106	7	1.170260889
SET	1	0.96	3.64E-62	6.01E-58	6	0.6188018
PKM	1	0.996	6.13E-58	1.01E-53	4	0.723541614
RPL34	1	0.997	1.89E-191	3.12E-187	9	1.568616255
RPL39	1	0.997	1.65E-173	2.73E-169	9	1.487788143
RPL10A	1	0.997	5.98E-186	9.86E-182	9	1.472888932
RPS2	1	0.997	1.31E-85	2.16E-81	9	1.281787958
RPL13A	1	0.997	8.86E-137	1.46E-132	9	1.25797475
RPS21	1	0.997	8.83E-149	1.46E-144	9	1.199248023
RPL39	1	0.997	7.77E-24	1.28E-19	14	1.059300204
RPL34	1	0.997	9.25E-92	1.53E-87	7	0.965248141
RPL24	1	0.997	1.70E-113	2.81E-109	9	0.960153857
RPL36	1	0.997	1.65E-103	2.71E-99	9	0.937796371
RPL39	1	0.997	8.72E-66	1.44E-61	7	0.820068585
RPL34	1	0.997	9.40E-40	1.55E-35	11	0.793831697
RPL10A	1	0.997	4.07E-55	6.71E-51	7	0.756938867
RPL10A	1	0.997	2.73E-13	4.50E-09	14	0.730136853
MT-ND1	1	0.997	1.81E-95	2.98E-91	0	0.7187152
RPL13A	1	0.997	7.21E-13	1.19E-08	14	0.718283558
RPL13A	1	0.997	7.25E-51	1.20E-46	7	0.681510569
RPS2	1	0.997	6.74E-09	0.000111251	14	0.646272448
RPL39	1	0.997	9.27E-26	1.53E-21	11	0.635473337
RPS21	1	0.997	4.95E-12	8.17E-08	14	0.627190765
RPS21	1	0.997	3.38E-47	5.57E-43	7	0.624023676
RPL34	1	0.997	1.51E-51	2.50E-47	5	0.623706174
RPL24	1	0.997	3.40E-13	5.61E-09	14	0.620725066
RPL13A	1	0.997	3.52E-25	5.81E-21	11	0.609788234
MTRNR2L12	1	0.998	2.37E-14	3.91E-10	15	2.185490689
LCP1	1	0.998	1.22E-75	2.00E-71	12	1.473535921
EEF2	1	0.998	3.19E-176	5.26E-172	9	1.452959599
RPL5	1	0.998	9.74E-178	1.61E-173	9	1.449101113
MTRNR2L12	1	0.998	1.97E-16	3.25E-12	12	1.289373246
RPL9	1	0.998	1.27E-152	2.10E-148	9	1.224869843
RPL37	1	0.998	3.74E-155	6.17E-151	9	1.208283702
RPL34	1	0.998	4.90E-28	8.08E-24	14	1.200830963
RPL35A	1	0.998	1.07E-143	1.76E-139	9	1.150976117
MTRNR2L12	1	0.998	4.63E-48	7.63E-44	0	1.120998897
RPL12	1	0.998	6.74E-110	1.11E-105	9	1.046121711
EEF1G	1	0.998	2.58E-110	4.26E-106	9	1.007770959
MT-ND4L	1	0.998	2.24E-35	3.69E-31	12	0.979037252
MT-ND5	1	0.998	1.16E-07	0.001910044	15	0.932066891
RPL4	1	0.998	7.21E-90	1.19E-85	9	0.907159624
RPL5	1	0.998	9.83E-19	1.62E-14	14	0.873629764
HMGB1	1	0.998	2.68E-97	4.42E-93	3	0.847319342

MTRNR2L12	1	0.998	1.13E-29	1.86E-25	5	0.821426892
RPL37	1	0.998	1.52E-19	2.51E-15	14	0.821309274
EEF2	1	0.998	4.14E-16	6.83E-12	14	0.813506284
RPL35A	1	0.998	1.06E-17	1.75E-13	14	0.796685913
MT-ND1	1	0.998	1.89E-25	3.11E-21	12	0.796138967
EEF2	1	0.998	1.24E-58	2.05E-54	7	0.793743467
CYC1	0.949	0.641	1.80E-65	2.97E-61	6	0.618751199
LCP1	1	0.998	2.31E-05	0.380473786	15	0.75583874
MT-CYB	1	0.998	9.16E-25	1.51E-20	12	0.741030821
MT-ND5	1	0.998	1.39E-20	2.30E-16	12	0.735905126
MYL12A	1	0.998	8.00E-94	1.32E-89	2	0.731415426
UBA52	1	0.998	4.57E-83	7.53E-79	9	0.728785696
MT-ATP6	1	0.998	1.27E-91	2.10E-87	0	0.725338144
RPL37	1	0.998	7.23E-66	1.19E-61	7	0.713065201
RPL9	1	0.998	2.35E-54	3.87E-50	7	0.663198195
EEF1G	1	0.998	9.20E-12	1.52E-07	14	0.657793581
RPL9	1	0.998	2.62E-11	4.33E-07	14	0.656680473
RPL37A	0.996	0.994	1.51E-49	2.49E-45	7	0.648859506
MT-ND5	1	0.998	1.53E-72	2.53E-68	0	0.642595009
RPL5	1	0.998	1.12E-44	1.85E-40	7	0.632101883
HNRNPA1	1	0.998	4.71E-48	7.77E-44	9	0.63044297
RPL35A	1	0.998	3.37E-52	5.55E-48	7	0.620418731
ZNF706	0.931	0.725	1.10E-53	1.82E-49	6	0.618219709
RPS13	1	0.999	1.01E-191	1.66E-187	9	1.507336762
PTPRC	1	0.999	7.86E-93	1.30E-88	12	1.493855154
RPS24	1	0.999	3.93E-190	6.48E-186	9	1.354528354
RPL18A	1	0.999	5.32E-179	8.77E-175	9	1.289627112
RPS5	1	0.999	1.76E-162	2.90E-158	9	1.252992694
RPL21	1	0.999	1.53E-146	2.52E-142	9	1.244111
RPS28	1	0.999	8.67E-149	1.43E-144	9	1.210672123
RPS27	1	0.999	4.58E-130	7.56E-126	9	1.203876548
RPLP2	1	0.999	2.78E-157	4.58E-153	9	1.16941073
RPL29	1	0.999	2.06E-151	3.40E-147	9	1.162503199
RPS14	1	0.999	2.94E-144	4.85E-140	9	1.15911355
RPS25	1	0.999	4.54E-140	7.49E-136	9	1.062806449
RPS16	1	0.999	1.31E-122	2.16E-118	9	1.026933263
RPS13	1	0.999	2.29E-24	3.77E-20	14	1.020016351
RPL23A	1	0.999	1.83E-121	3.01E-117	9	1.014535847
RPS27	1	0.999	2.77E-92	4.56E-88	7	0.927702922
MT-ATP6	1	0.999	2.14E-26	3.53E-22	12	0.915115752
RPS27	1	0.999	6.71E-55	1.11E-50	11	0.911497805
CD74	1	0.999	1.44E-30	2.38E-26	10	0.905068646
GTF3A	0.989	0.893	2.24E-68	3.69E-64	6	0.617990353
MT-CO2	1	0.999	4.23E-29	6.98E-25	12	0.864932123
NACA	1	0.999	1.36E-96	2.25E-92	9	0.862493303
RPS27	1	0.999	1.97E-17	3.25E-13	14	0.85788815
RPL21	1	0.999	2.07E-18	3.42E-14	14	0.855338473

MT-CO3	1	0.999	1.66E-25	2.75E-21	12	0.830695983
RPS14	1	0.999	1.14E-45	1.88E-41	11	0.824215554
RPS14	1	0.999	9.06E-18	1.49E-13	14	0.812213682
MT-CO1	1	0.999	8.52E-28	1.41E-23	12	0.808450471
MT-ATP6	1	0.999	5.08E-06	0.083716677	15	0.804349523
RPS28	1	0.999	1.05E-17	1.73E-13	14	0.792255155
RPS28	1	0.999	7.21E-71	1.19E-66	7	0.780379675
RPS13	1	0.999	2.74E-59	4.51E-55	7	0.771024785
RPL21	1	0.999	4.78E-57	7.89E-53	7	0.728227112
RAC2	1	0.999	1.23E-97	2.03E-93	2	0.703480778
RPS24	1	0.999	3.12E-62	5.15E-58	7	0.699731451
RPS14	1	0.999	3.78E-59	6.23E-55	7	0.684744046
MT-CO2	1	0.999	2.29E-85	3.78E-81	0	0.680099495
RPS25	1	0.999	3.55E-39	5.86E-35	11	0.655500027
MT-CO3	1	0.999	1.64E-72	2.71E-68	0	0.646154502
RPL29	1	0.999	3.52E-13	5.80E-09	14	0.644682968
RPS25	1	0.999	1.21E-13	1.99E-09	14	0.643162533
LSM5	0.939	0.619	2.93E-61	4.83E-57	6	0.61774301
RPLP2	1	0.999	9.84E-14	1.62E-09	14	0.632799631
RPL21	1	0.999	3.21E-22	5.29E-18	11	0.623651468
RPS28	1	0.999	6.83E-26	1.13E-21	11	0.606241793
CD74	1	0.999	1.85E-07	0.003047384	12	0.59816345
MT-CO1	1	0.999	0.000118874	1	15	0.597889801
RPLP2	1	0.999	4.13E-46	6.82E-42	7	0.594976706
EEF1A1	1	1	1.03E-216	1.71E-212	9	1.715057728
RPS3A	1	1	4.64E-226	7.66E-222	9	1.573439662
RPL10	1	1	1.36E-201	2.25E-197	9	1.483741853
RPS8	1	1	4.21E-213	6.94E-209	9	1.474159591
RPL32	1	1	5.14E-203	8.48E-199	9	1.465331106
RPS6	1	1	3.84E-182	6.34E-178	9	1.376612844
RPS18	1	1	2.10E-149	3.47E-145	9	1.327617922
EEF1A1	1	1	8.83E-34	1.46E-29	14	1.315901423
RPL13	1	1	7.85E-174	1.29E-169	9	1.311996744
RPL6	1	1	2.70E-180	4.45E-176	9	1.309183596
RPL26	1	1	1.72E-170	2.84E-166	9	1.305942844
RPL30	1	1	3.50E-174	5.78E-170	9	1.293959995
RPS12	1	1	3.22E-160	5.31E-156	9	1.285966848
RPL3	1	1	5.20E-157	8.58E-153	9	1.254626269
RPS23	1	1	6.71E-150	1.11E-145	9	1.171985563
RPL18	1	1	1.07E-169	1.77E-165	9	1.14794057
RPS7	1	1	3.41E-143	5.62E-139	9	1.143537523
S100A11	1	0.977	2.10E-44	3.46E-40	6	0.612823048
RPL19	1	1	8.59E-148	1.42E-143	9	1.110961263
RPS27A	1	1	3.91E-148	6.45E-144	9	1.099057287
RPS4X	1	1	1.00E-138	1.65E-134	9	1.096585046
EIF1	1	1	1.03E-129	1.71E-125	9	1.095937323
RPL11	1	1	1.84E-156	3.04E-152	9	1.095732013

FTH1	1	1	4.27E-70	7.05E-66	9	1.086124035
EEF1A1	1	1	1.25E-96	2.07E-92	7	1.066495533
RACK1	1	1	3.18E-152	5.24E-148	9	1.055583668
RPS3A	1	1	1.39E-26	2.30E-22	14	1.048992323
RPS15A	1	1	1.57E-125	2.59E-121	9	1.036108008
RPL17	1	1	2.71E-116	4.47E-112	9	1.030439924
DDX39A	0.957	0.714	5.18E-62	8.54E-58	6	0.611965494
RPL15	1	1	5.79E-128	9.55E-124	9	1.015541544
TUFM	0.939	0.759	3.60E-62	5.93E-58	6	0.611543402
MRPS34	0.936	0.678	1.29E-58	2.13E-54	6	0.610159821
RPLP1	1	1	1.49E-105	2.45E-101	9	0.977364737
RPL10	1	1	5.82E-94	9.60E-90	7	0.947925983
RPL30	1	1	1.31E-23	2.15E-19	14	0.93449138
RPL7A	1	1	7.37E-115	1.22E-110	9	0.9293874
RPL3	1	1	6.13E-22	1.01E-17	14	0.922818488
RPL8	1	1	6.58E-84	1.09E-79	6	0.605963
MALAT1	1	1	9.61E-71	1.59E-66	5	0.919777684
RPL32	1	1	3.43E-21	5.66E-17	14	0.913234356
RPL41	1	1	4.58E-119	7.56E-115	9	0.909118088
B2M	1	1	8.64E-136	1.42E-131	2	0.904095139
RPS15A	1	1	9.18E-24	1.51E-19	14	0.900191839
EIF1	1	1	7.17E-99	1.18E-94	7	0.888802092
MALAT1	1	1	1.16E-12	1.92E-08	14	0.864832359
RPL10	1	1	4.16E-18	6.86E-14	14	0.853238341
RPSA	1	1	3.74E-93	6.16E-89	9	0.849731598
RPL8	1	1	1.40E-90	2.31E-86	9	0.837892648
RPS6	1	1	3.53E-17	5.82E-13	14	0.829216756
MALAT1	1	1	1.37E-54	2.26E-50	7	0.822092312
RPLP1	1	1	3.18E-18	5.24E-14	14	0.817110044
RPL14	1	1	6.68E-91	1.10E-86	9	0.802701918
RPS3	1	1	3.44E-104	5.68E-100	9	0.799824274
RPS9	1	1	1.80E-76	2.97E-72	9	0.795105512
RPS3A	1	1	8.36E-70	1.38E-65	7	0.795084008
PPA1	0.973	0.796	1.79E-50	2.95E-46	6	0.605710008
HLA-B	1	1	5.80E-28	9.57E-24	12	0.773335718
EIF1	1	1	8.43E-18	1.39E-13	14	0.76488648
RPL26	1	1	1.47E-70	2.42E-66	7	0.756242582
RPS12	1	1	8.67E-15	1.43E-10	14	0.751506212
RPL30	1	1	6.82E-71	1.13E-66	7	0.748733022
RPL6	1	1	2.13E-16	3.52E-12	14	0.746677071
RPS27A	1	1	1.72E-17	2.84E-13	14	0.744569687
TOMM6	0.944	0.671	4.62E-60	7.63E-56	6	0.602965349
SH3BGRL3	1	1	3.12E-86	5.14E-82	2	0.73560593
RPL13	1	1	9.65E-15	1.59E-10	14	0.722627407
RPL28	1	1	2.83E-71	4.67E-67	9	0.70649122
RPL11	1	1	1.62E-16	2.67E-12	14	0.689032258
B2M	1	1	1.04E-36	1.72E-32	10	0.68850196

RPS7	1	1	1.05E-14	1.74E-10	14	0.687025769
SNRPB	0.987	0.947	7.31E-75	1.21E-70	6	0.601986562
RPL13	1	1	1.76E-59	2.91E-55	7	0.68224386
RPL30	1	1	1.23E-34	2.03E-30	11	0.681401835
ACTB	1	1	1.05E-68	1.73E-64	1	0.676335612
RPS18	1	1	9.67E-12	1.59E-07	14	0.675611624
PTMA	1	1	1.15E-68	1.89E-64	3	0.674104036
RPL12	1	0.998	3.15E-65	5.19E-61	6	0.598200984
RPL10	1	1	4.78E-29	7.89E-25	11	0.67325764
RPL32	1	1	1.01E-54	1.66E-50	7	0.672218395
RPL3	1	1	3.44E-54	5.67E-50	7	0.669183857
VIM	1	1	8.03E-63	1.32E-58	1	0.665186776
C12orf75	0.981	0.911	5.24E-51	8.64E-47	6	0.596083266
RPS15A	1	1	3.10E-43	5.11E-39	11	0.659871194
RPLP0	1	1	1.59E-47	2.63E-43	9	0.659396123
RPL3	1	1	6.75E-32	1.11E-27	11	0.656478868
EEF1A1	1	1	3.07E-21	5.06E-17	11	0.656098338
FAU	1	1	1.30E-76	2.15E-72	9	0.653190076
HLA-C	1	1	1.23E-22	2.03E-18	12	0.649922161
RPS18	1	1	1.38E-46	2.28E-42	7	0.639939577
RPL17	1	1	1.01E-11	1.67E-07	14	0.639543362
MT-ND4L	0.998	0.998	6.92E-78	1.14E-73	0	0.638578651
RPS27A	1	1	4.71E-56	7.76E-52	7	0.636317707
VIM	1	1	1.46E-44	2.40E-40	6	0.594966834
RPL6	1	1	1.50E-50	2.48E-46	7	0.623939633
RPL19	1	1	4.35E-13	7.18E-09	14	0.622507324
RPS23	1	1	2.68E-11	4.43E-07	14	0.612077051
RPL18	1	1	7.59E-13	1.25E-08	14	0.608986418
PTTG1	0.936	0.614	1.70E-50	2.80E-46	6	0.587363716
H3F3B	1	1	2.21E-14	3.64E-10	14	0.605089387
RPS4X	1	1	1.21E-11	2.00E-07	14	0.601072589
TPT1	1	1	3.70E-49	6.10E-45	9	0.598275926
EIF1	1	1	3.57E-23	5.88E-19	11	0.595870972
SDF2L1	0.912	0.686	3.75E-41	6.19E-37	6	0.586548267
RPL19	1	1	1.63E-49	2.68E-45	7	0.593758774
RPS12	1	1	8.41E-43	1.39E-38	7	0.592544572
RPS8	1	1	3.27E-11	5.39E-07	14	0.592406222
RPS15A	1	1	2.41E-52	3.97E-48	7	0.591889332
MT-CO1	0.998	0.999	1.89E-79	3.12E-75	0	0.621040188
HIST1H4C	0.911	0.913	8.63E-11	1.42E-06	8	0.765494785
PPIB	0.99	0.992	3.07E-09	5.07E-05	12	0.619892065
MT-ND5	0.99	0.998	4.28E-16	7.07E-12	13	0.612306012

Table S14: Gene co-expression modules (GM) of 16 REP $_{\gamma\delta}$ cell clusters

GM1	GM2	GM3	GM4	GM5	GM6	GM7	GM8	GM9	GM10	GM11	GM12
ADGRE5	AL133415.1	EEF1B2	BHLHE40	BST2	C1QBP	CCT3	BTG2	ATP5F1B	CHD4	BTF3	CD3D
BTG1	ARL6IP1	EEF1G	CD63	CD164	CD81	CCT5	CD69	ATP5MC3	DIAPH1	COMMD6	CD3G
CIRBP	ASPM	EIF3G	CD82	CD53	GPX4	CCT6A	DDX3X	C12orf75	DOCK8	EEF1A1	CD52
FYN	ATAD2	FAU	CD96	DPP4	CCT2	CCT7	SRGN	CALR	DYNC1H1	EEF2	HLA-B
IL7R	BIRC5	NACA	FURIN	HOPX	CCT4	PTPN7	IFITM1	CANX	EIF3A	EIF3E	HLA-C
NR4A2	CALM2	PFDN5	GZMB	IL32	CCT8	BANF1	IFITM2	CCND2	FLNA	EIF3F	IL2RG
DDX5	CARHSP1	RACK1	IFI16	LGALS3	EIF4A1	CACYBP	PRMT2	COTL1	GPRIN3	EIF3L	ISG15
KLRD1	CCNA2	RPL10A	IQGAP1	S100A4	EIF5A	CHCHD2	ARGLU1	DDOST	HUWE1	HNRNPA1	ISG20
NFKB1	CDK1	RPL11	BATF	CD74	ETS1	COX5A	BCLAF1	FKBP1A	KTN1	RPL10	LAT
NFKBIA	CENPF	RPL12	CCL5	FUT7	FTH1	CYC1	BRD2	FTL	MACF1	RPL15	LIMD2
AREG	CENPM	RPL13	NKG7	SELL	GTF3A	ENO1	CCNL1	GPI	MT-ATP6	RPL18	LTB
ATP6V0C	CKAP5	RPL13A	PRF1	ACTB	H1FX	FABP5	CD37	HNRNPF	MT-ATP8	RPL22	MAL
BZW1	CRIP1	RPL14	ACTN4	ACTG1	HMGA1	GAPDH	ELF1	LDHA	MT-CO1	RPL39	PIM2
C12orf57	DDX39A	RPL17	ACTR2	ACTR3	HNRNPAB	HSP90AA1	FOS	MANF	MT-CO2	RPL5	PSAP
CHD1	DEK	RPL18A	ADAM19	ALOX5AP	HNRNPD	HSPA4	GZMM	NDUFAB1	MT-CO3	RPL6	RNF149
CMC1	DUT	RPL19	AHNAK	ARL6IP5	HNRNPL	HSPA8	HCST	P4HB	MT-CYB	RPL7	SHISA5
CSRNP1	FAM111A	RPL21	AKAP13	B2M	HNRNPU	MICOS10	HLA-E	PDIA3	MT-ND1	RPLP1	TPST2
CUTA	H2AFV	RPL23	ANTXR2	BAX	HSP90AB1	MIF	IER2	PDIA6	MT-ND3	RPS13	UCP2
CXCR4	H2AFX	RPL23A	ATP2B4	CDC25B	HSP90B1	MRPL37	JAK1	PFN1	MT-ND4	RPS14	XBP1
CYCS	H2AFZ	RPL24	CASP8	CLU	HSPA5	MRPS15	JUN	PGAM1	MT-ND4L	RPS24	
DDIT4	HIST1H1B	RPL26	CBLB	CTSC	HSPD1	MRPS34	JUNB	PGD	MT-ND5	RPS28	
DDX3Y	HIST1H1C	RPL27	CKLF	DSTN	HSPE1	NHP2	KLF6	PPA1	MT-ND6	RPS3A	
DNAJA1	HIST1H1D	RPL27A	CLEC2D	EMP3	JUND	NME1	LITAF	PPIA	MTRNR2L12	RPS7	
DNAJB1	HIST1H1E	RPL28	CST7	GLUL	KLF2	NME2	MALAT1	PPIB	MYH9	SLC25A6	
DUSP1	HIST1H4C	RPL29	CTSD	GZMA	LINC01578	PA2G4	MCL1	PRDX1	RUNX3	SNHG29	
DUSP2	HMGB1	RPL3	CTSW	HLA-DMA	MKNK2	PDCD5	NOSIP	PRDX3	SPTBN1		
EIF1	HMGB2	RPL30	DDX17	HLA-DPA1	NCL	PHB	PDE4D	RPN1	TFRC		
EIF4B	HMGN2	RPL31	ESYT1	HLA-DPB1	NPM1	PKM	PNISR	RPN2	TLN1		
EIF5	KIF22	RPL32	GBP2	HLA-DQA2	PEBP1	PRELID1	PPP1R15A	SDF2L1	TNFRSF1B		
ELL2	KNL1	RPL34	GBP5	HLA-DQB1	PTGES3	RAN	PRKCH	SEC61B			
EZR	LBR	RPL35A	GLG1	HLA-DRA	RPS2	RANBP1	RBM39	SRPRA			
FOSB	LMNB1	RPL36	GNPTAB	HLA-DRB1	RPS4Y1	RPS26	RHOH	SURF4			
FUS	LSM5	RPL36A	HELZ	HLA-DRB5	SET	SNRPB	RSRP1	TXN			
GADD45B	MCM7	RPL37	IKZF2	ITGB7	SFPQ	SNRPD1	SF3B1	ZNF706			
GAS5	MKI67	RPL37A	IL2RB	JPT1	SLC25A5	SNRPE	SLC2A3				
GYPC	NCAPH	RPL38	IQGAP2	KDSR	SMAP2	SNRPG	SRSF5				
H3F3B	NUCKS1	RPL4	ITGB1	LGALS1	SRRM2	SQOR	VAMP2				
HNRNPA0	NUDT1	RPL41	ITGB2	LY6E	SRSF9	SRA1	ZC3HAV1				
HNRNPDL	NUSAP1	RPL7A	LCP1	MYL12A	TAGLN2	STIP1	ZFP36				
IDI1	PCLAF	RPL8	LCP2	PPP1R18	TGFB1	STOML2	ZFP36L1				
ITGA4	PRKDC	RPL9	MSN	RAC2	TOMM22	TCP1					
ITGAD	PTMA	RPLP0	MYO1F	RNASEK	TOMM6	TPI1					
LEPROTL1	PTTG1	RPLP2	NEAT1	S100A11	TUFM						
MAP3K8	RAD21	RPS11	NIBAN1	S100A6	UBE2S						
MSL2	RFC1	RPS12	NUCB1	SELPLG	VDAC1						
NOP53	RRM2	RPS15A	PRNP	SH3BGRL3	YBX1						
PABPC1	SIVA1	RPS16	PRRC2C	SH3BP1							
PIK3IP1	SMC1A	RPS17	PTPN22	SH3BP5							
PIM3	SMC2	RPS18	PTPRC	SLC9A3R1							
PNRC1	SMC3	RPS20	RESF1	TAP1							
RBM38	SMC4	RPS21	RNF213	TIMP1							
REL	SSRP1	RPS23	SETX	TMBIM6							
RGCC	STMN1	RPS25	SLC38A1	TSPO							
SARAF	TK1	RPS27	SPN	VIM							
SF1	TMPO	RPS27A	STAT1								
SOX4	TOP2A	RPS29	SYNE2								
SRSF7	TPX2	RPS3	TAPBP								
STK17A	TUBA1B	RPS4X	TNFSF10								
TCF7	TUBA1C	RPS5	TRERF1								
TNFAIP3	TUBA4A	RPS6									
TSC22D3	TUBB	RPS8									
TSPYL2	TUBB4B	RPS9									
TUBA1A	TYMS	RPSA									
YPEL5	UBE2C	TPT1									
ZFP36L2	USP1	UBA52									
ZNF331											

Table S15: GO enrichment analysis of the 12 gene co-expression modules (GM)

GM	Genes	GO enrichment (PANTHER pathway)						GO enrichment (GO-Slim BP)					
		Pathway	Gene list size	Observed	Expected	Enrichment	FDR	Pathway	Gene list size	Observed	Expected	Enrichment	FDR
1	ADGRE5,AREG,ATP6V0C,BTG1,BZW1,C12orf57,CHD1,CIRBP,CMC1,CSRNP1,CUTA,CXCR4,CYCS,DDIT4,DDX3Y,DDX5,DNAJA1,DNAJB1,DUSP1,DUSP2,EIF1,EIF4B,EIF5,ELL2,EZR,FOSB,FUS,FYN,GADD45B,GAS5,GYPC,H3F3B,HNRNPA0,HNRNPDL,IDI1,IL7R,ITGA4,ITGAD,KLRD1,LEPROTL1,MAP3K8,MSL2,NFKB1,NFKBIA,NOP53,NR4A2,PABPC1,PIK3IP1,PIM3,PNRC1,RBM38,REL,RGCC,SARAF,SF1,SOX4,SRSF7,STK17A,TCF7,TNFAIP3,TSC22D3,TSPYL2,TUBA1A,YPEL5,ZFP36L2,ZNF331	Toll receptor signaling pathway (P00054)	61	5	0.2	25.19	3.78E-04	No significantly enriched GO-BP					
		Apoptosis signaling pathway (P00006)	125	4	0.41	9.83	3.28E-02						
		Gonadotropin-releasing hormone receptor pathway (P06664)	237	6	0.77	7.78	1.09E-02						
2	AL133415.1,ARL6IP1,ASPM,ATAD2,BIRC5,CALM2,CARHSP1,CCNA2,CDK1,CENPF,CENPM,CKAP5,CRIP1,DDX39A,DEK,DUT,FAM111A,H2AFV,H2AFX,H2AFZ,HIST1H1B,HIST1H1C,HIST1H1D,HIST1H1E,HIST1H4C,HMGB1,HMGB2,HMGN2,KIF22,KNL1,LBR,LMNB1,LSM5,MCM7,MKI67,NCAPH,NUCKS1,NUDT1,NUSAP1,PCLAF,PRKDC,PTMA,PTTG1,RAD21,RFC1,RRM2,SIVA1,SMC1A,SMC2,SMC3,SMC4,SSRP1,STMN1,TK1,TMPO,TOP2A,TPX2,TUBA1B,TUBA1C,TUBA4A,TUBB,TUBB4B,TYMS,UBE2C,USP1	De novo pyrimidine deoxyribonucleotide biosynthesis (P02739)	15	3	0.04	69.79	2.81E-03	mitotic chromosome condensation (GO:0007076)	6	2	0.02	> 100	1.90E-02
								pyrimidine nucleotide biosynthetic process (GO:0006221)	1.00E+01	2	0.03	69.79	4.12E-02
								pyrimidine nucleotide metabolic process (GO:0006220)	1.20E+01	2	0.03	58.16	4.81E-02
								pyrimidine-containing compound metabolic process (GO:0072527)	2.40E+01	3	0.07	43.62	6.23E-03
								sister chromatid segregation (GO:0000819)	7.00E+01	6	0.2	29.91	1.45E-05
								mitotic sister chromatid segregation (GO:0000070)	56	4	0.16	24.93	2.89E-03
								nuclear chromosome segregation (GO:0098813)	101	7	0.29	24.19	5.33E-06
								chromosome segregation (GO:0007059)	118	8	0.34	23.66	7.28E-07
								DNA packaging (GO:0006323)	54	3	0.15	19.39	4.45E-02
								DNA conformation change (GO:0071103)	74	4	0.21	18.86	6.77E-03
								biological phase (GO:0044848)	57	3	0.16	18.37	4.84E-02
								cell cycle phase (GO:0022403)	57	3	0.16	18.37	4.69E-02
								mitotic cell cycle (GO:0000278)	271	13	0.78	16.74	4.90E-10
								mitotic cell cycle process (GO:1903047)	271	13	0.78	16.74	4.08E-10
								mitotic nuclear division (GO:0140014)	271	13	0.78	16.74	3.50E-10
								double-strand break repair (GO:0006302)	109	5	0.31	16.01	2.29E-03
								nuclear division (GO:0000280)	326	14	0.93	14.99	4.16E-10
								organelle fission (GO:0048285)	343	14	0.98	14.24	6.05E-10
								chromosome organization (GO:0051276)	256	10	0.73	13.63	9.46E-07
								cell cycle process (GO:0022402)	442	16	1.27	12.63	2.69E-10
								DNA recombination (GO:0006310)	115	4	0.33	12.14	3.07E-02
								cell cycle (GO:0007049)	476	16	1.36	11.73	4.03E-10
								cellular response to DNA damage stimulus (GO:0006974)	252	8	0.72	11.08	1.23E-04
								DNA repair (GO:0006281)	197	6	0.56	10.63	2.86E-03
								DNA metabolic process (GO:0006259)	324	7	0.93	7.54	4.50E-03
								microtubule cytoskeleton organization (GO:0000226)	373	8	1.07	7.48	1.59E-03
								microtubule-based process (GO:0007017)	488	9	1.4	6.44	1.51E-03
								cellular response to stress (GO:0033554)	480	8	1.38	5.82	6.83E-03
								cytoskeleton organization (GO:0007010)	723	9	2.07	4.34	1.92E-02
								organelle organization (GO:0006996)	1879	21	5.38	3.9	5.27E-06
								cellular component organization (GO:0016043)	2518	21	7.22	2.91	5.44E-04
								cellular component organization or biogenesis (GO:0071840)	2644	21	7.58	2.77	1.10E-03
								organic cyclic compound metabolic process (GO:1901360)	3041	19	8.71	2.18	4.97E-02
	EEF1B2,EEF1G,EIF3G,FAU,NACA,PFDN5,RACK1,RPL10A,RPL11,RPL12,RPL13,RPL13A,RPL14,RPL17,RPL18A,RPL19,RPL21,RPL23,RPL23A,RPL24,RPL26,RPL27,RPL27A,RPL28,RPL29,RPL3,RPL30,RPL31,RPL32,RPL34,RPL35A,RPL36,RPL36A,RPL37,RPL37A,RPL38,RPL4,RPL41,RPL7A,RPL8,RPL9,RPLP0,RPLP2,RPS11,RPS12,RPS15A,RPS16,RPS17,RPS18,RPS20,RPS21,RPS23,RPS25,RPS27,RPS27A,RPS29,RPS3,RPS4X,RPS5,RPS6,RPS8,RPS9,RPSA,TPT1,UBA52							cytoplasmic translation (GO:0002181)	48	11	0.15	72.59	9.71E-15
								ribosomal large subunit assembly (GO:0000027)	18	3	0.06	52.79	3.67E-03
								ribosomal small subunit biogenesis (GO:0042274)	52	6	0.16	36.55	5.05E-06
								ribosomal large subunit biogenesis (GO:0042273)	52	6	0.16	36.55	4.66E-06
								maturation of SSU-rRNA from tricistronic rRNA transcript (SSU-rRNA, 5.	28	3	0.09	33.94	9.61E-03
								maturation of SSU-rRNA (GO:0030490)	37	3	0.12	25.68	1.94E-02
								translational elongation (GO:0006414)	254	20	0.8	24.94	5.23E-19
								translation (GO:0006412)	254	20	0.8	24.94	2.62E-19
								peptide biosynthetic process (GO:0043043)	257	20	0.81	24.65	2.17E-19
								ribosome biogenesis (GO:0042254)	167	12	0.53	22.76	8.25E-11
								amide biosynthetic process (GO:0043604)	295	20	0.93	21.47	2.14E-18
								ribonucleoprotein complex assembly (GO:0022618)	95	6	0.3	20.01	1.14E-04
								peptide metabolic process (GO:0006518)	318	20	1	19.92	6.98E-18
								ribonucleoprotein complex subunit organization (GO:0071826)	99	6	0.31	19.2	1.34E-04
								ribonucleoprotein complex biogenesis (GO:0022613)	223	12	0.7	17.05	1.87E-09
								cellular amide metabolic process (GO:0043603)	412	20	1.3	15.38	7.33E-16
								non-membrane-bounded organelle assembly (GO:0140694)	150	6	0.47	12.67	9.73E-04
								organonitrogen compound biosynthetic process (GO:1901566)	692	20	2.18	9.15	7.91E-12
								cellular protein metabolic process (GO:0044267)	1765	23	5.57	4.13	4.05E-07
								cellular component biogenesis (GO:0044085)	1024	12	3.23	3.71	6.71E-03
								protein metabolic process (GO:0019538)	2090	23	6.6	3.49	7.60E-06
								cellular macromolecule biosynthetic process (GO:0034645)	2326	21	7.34	2.86	6.59E-04
								macromolecule biosynthetic process (GO:0009059)	2332	21	7.36	2.85	6.51E-04
								organonitrogen compound metabolic process (GO:1901564)	2573	23	8.12	2.83	2.56E-04
								cellular nitrogen compound biosynthetic process (GO:0044271)	2352	21	7.43	2.83	7.09E-04
								gene expression (GO:0010467)	2894	24	9.14	2.63	4.95E-04
								cellular biosynthetic process (GO:0044249)	2731	21	8.62	2.44	5.73E-03

								organic substance biosynthetic process (GO:1901576)	2773	21	8.75	2.4	6.88E-03
								biosynthetic process (GO:0009058)	2778	21	8.77	2.39	6.56E-03
								cellular nitrogen compound metabolic process (GO:0034641)	3265	24	10.31	2.33	4.09E-03
								cellular macromolecule metabolic process (GO:0044260)	3864	24	12.2	1.97	4.23E-02
								regulation of biological process (GO:0050789)	5162	5	16.3	0.31	3.45E-02
								biological regulation (GO:0065007)	5616	5	17.73	0.28	9.35E-03
3		No significantly enriched pathways found						response to stimulus (GO:0050896)	3119	1	9.85	0.1	4.46E-02
4	ACTN4,ACTR2,ADAM19,AHNAK,AKAP13,ANTXR2,ATP2B4,BATF,BHLHE40,CASP8,CBLB,CCL5,CD63,CD82,CD96,CKLF,CLEC2D,CST7,CTSD,CTSW,DDX17,ESYT1,FURIN,GBP2,GBP5,GLG1,GNPTAB,GZMB,HELZ,IFI16,IKZF2,IL2RB,IQGAP1,IQGAP2,ITGB1,ITGB2,LCP1,LCP2,MSN,MYO1F,NEAT1,NIBAN1,NKG7,NUCB1,PRF1,PRNP,PRRC2C,PTPN22,PTPRC,RESF1,RNF213,SETX,SLC38A1,SPN,STAT1,SYNE2,TAPBP,TNFSF10,TRERF1	No significantly enriched pathways found						No significantly enriched GO-BP terms found					
	ACTB,ACTG1,ACTR3,ALOX5AP,ARL6IP5,B2M,BAX,BST2,CD164,CD53,CD74,CDC25B,CLU,CTSC,DPP4,DSTN,EMP3,FUT7,GLUL,GZMA,HLA-DMA,HLA-DPA1,HLA-DPB1,HLA-DQA2,HLA-DQB1,HLA-DRA,HLA-DRB1,HLA-DRB5,HOPX,IL32,ITGB7,JPT1,KDSR,LGALS1,LGALS3,LY6E,MYL12A,PPP1R18,RAC2,RNASEK,S100A11,S100A4,S100A6,SELL,SELPLG,SH3BGRL3,SH3BP1,SH3BP5,SLC9A3R1,TAP1,TIMP1,TMBIM6,TSPO,VIM	T cell activation (P00053)	92	8	0.25	31.97	4.28E-08	antigen processing and presentation (GO:0019882)	41	11	0.11	98.64	1.27E-15
		Huntington disease (P00029)	152	4	0.41	9.68	4.53E-02	positive regulation of leukocyte cell-cell adhesion (GO:1903039)	39	10	0.11	94.27	5.17E-14
		Inflammation mediated by chemokine and cytokine signaling pathw	261	5	0.71	7.04	6.11E-02	positive regulation of T cell activation (GO:0050870)	39	10	0.11	94.27	3.88E-14
								positive regulation of cell-cell adhesion (GO:0022409)	40	10	0.11	91.92	3.87E-14
								leukocyte cell-cell adhesion (GO:0007159)	57	12	0.16	77.4	7.83E-16
								positive regulation of cell adhesion (GO:0045785)	48	10	0.13	76.6	1.61E-13
								regulation of leukocyte cell-cell adhesion (GO:1903037)	51	10	0.14	72.09	2.37E-13
								regulation of T cell activation (GO:0050863)	53	10	0.14	69.37	2.93E-13
								regulation of cell-cell adhesion (GO:0022407)	57	10	0.16	64.5	4.51E-13
								negative regulation of apoptotic signaling pathway (GO:2001234)	21	3	0.06	52.52	1.99E-03
								T cell activation (GO:0042110)	83	11	0.23	48.73	2.96E-13
								regulation of cell adhesion (GO:0030155)	80	10	0.22	45.96	9.04E-12
								regulation of apoptotic signaling pathway (GO:2001233)	31	3	0.08	35.58	5.38E-03
								production of molecular mediator of immune response (GO:0002440)	125	11	0.34	32.35	1.44E-11
								B cell mediated immunity (GO:0019724)	118	10	0.32	31.16	2.61E-10
								immunoglobulin mediated immune response (GO:0016064)	118	10	0.32	31.16	2.43E-10
								positive regulation of lymphocyte activation (GO:0051251)	132	10	0.36	27.85	6.11E-10
								positive regulation of leukocyte activation (GO:0002696)	137	10	0.37	26.84	7.73E-10
								positive regulation of cell activation (GO:0050867)	137	10	0.37	26.84	7.35E-10
								adaptive immune response based on somatic recombination of immun	142	10	0.39	25.89	9.33E-10
								lymphocyte mediated immunity (GO:0002449)	147	10	0.4	25.01	1.23E-09
								regulation of lymphocyte activation (GO:0051249)	148	10	0.4	24.84	1.26E-09
								leukocyte mediated immunity (GO:0002443)	154	10	0.42	23.87	1.69E-09
								regulation of leukocyte activation (GO:0002694)	157	10	0.43	23.42	1.94E-09
								regulation of cell activation (GO:0050865)	159	10	0.43	23.12	2.11E-09
								adaptive immune response (GO:0002250)	165	10	0.45	22.28	2.88E-09
								immune effector process (GO:0002252)	182	11	0.5	22.22	5.13E-10
								lymphocyte activation (GO:0046649)	188	11	0.51	21.51	6.36E-10
								cell-cell adhesion (GO:0098609)	214	12	0.58	20.62	1.38E-10
								leukocyte activation (GO:0045321)	198	11	0.54	20.43	9.29E-10
								cell activation (GO:0001775)	212	11	0.58	19.08	1.57E-09
								apoptotic signaling pathway (GO:0097190)	80	4	0.22	18.38	4.12E-03
								positive regulation of immune system process (GO:0002684)	254	11	0.69	15.92	7.85E-09
								cell adhesion (GO:0007155)	398	13	1.08	12.01	4.11E-09
								biological adhesion (GO:0022610)	398	13	1.08	12.01	3.98E-09
								regulation of immune system process (GO:0002682)	339	11	0.92	11.93	1.40E-07
								cellular protein-containing complex assembly (GO:0034622)	348	10	0.95	10.56	2.34E-06
								protein-containing complex assembly (GO:0065003)	367	10	1	10.02	3.61E-06
								protein-containing complex subunit organization (GO:0043933)	452	11	1.23	8.95	2.31E-06
								regulation of apoptotic process (GO:0042981)	166	4	0.45	8.86	4.99E-02
								immune response (GO:0006955)	541	12	1.47	8.16	1.47E-06
								immune system process (GO:0002376)	694	15	1.89	7.95	2.78E-08
								localization of cell (GO:0051674)	322	6	0.88	6.85	1.18E-02
								cell motility (GO:0048870)	322	6	0.88	6.85	1.16E-02
								locomotion (GO:0040011)	394	6	1.07	5.6	3.18E-02
								cellular component assembly (GO:0022607)	896	10	2.44	4.1	6.90E-03
								positive regulation of cellular process (GO:0048522)	1227	13	3.34	3.9	1.15E-03
								positive regulation of biological process (GO:0048518)	1365	14	3.71	3.77	7.64E-04
								cellular component biogenesis (GO:0044085)	1024	10	2.79	3.59	1.84E-02
								cellular component organization (GO:0016043)	2518	18	6.85	2.63	4.19E-03
								cellular component organization or biogenesis (GO:0071840)	2644	18	7.19	2.5	7.03E-03
5								response to stimulus (GO:0050896)	3119	20	8.48	2.36	6.21E-03
								telomere maintenance via telomerase (GO:0007004)	8	2	0.02	> 100	4.59E-02
								telomere maintenance via telomere lengthening (GO:0010833)	11	2	0.02	83.19	4.17E-02
								protein folding (GO:0006457)	107	9	0.23	38.48	6.79E-09
								positive regulation of gene expression (GO:0010628)	126	4	0.28	14.52	4.53E-02
								translational elongation (GO:0006414)	254	5	0.56	9.01	4.90E-02

6	C1QBP,CCT2,CCT4,CCT8,CD81,EIF4A1,EIF5A,ETS1,FTH1,GPX4,GTF3A,H1FX,HMGA1,HNRNPAB,HNRNPD,HNRNPL,HNRNPU,HSP90AB1,HSP90B1,HSPA5,HSPD1,HSPE1,JUND,KLF2,LINC01578,MKNK2,NCL,NPM1,PEBP1,PTGES3,RPS2,RPS4Y1,SET,SFPQ,SLC25A5,SMAP2,SRRM2,SRSF9,TAGLN2,TGFB1,TOMM22,TOMM6,TUFM,UBE2S,VDAC1,YBX1	No significantly enriched pathways found						translation (GO:0006412)	254	5	0.56	9.01	4.50E-02
								peptide biosynthetic process (GO:0043043)	257	5	0.56	8.9	4.38E-02
								regulation of gene expression (GO:0010468)	2106	15	4.6	3.26	1.38E-02
								gene expression (GO:0010467)	2894	20	6.33	3.16	6.20E-04
								regulation of macromolecule metabolic process (GO:0060255)	2533	16	5.54	2.89	1.68E-02
								cellular macromolecule biosynthetic process (GO:0034645)	2326	14	5.08	2.75	4.87E-02
								regulation of metabolic process (GO:0019222)	2659	16	5.81	2.75	2.65E-02
								macromolecule biosynthetic process (GO:0009059)	2332	14	5.1	2.75	4.67E-02
								cellular nitrogen compound biosynthetic process (GO:0044271)	2352	14	5.14	2.72	4.25E-02
								macromolecule metabolic process (GO:0043170)	4815	26	10.52	2.47	8.80E-04
								cellular nitrogen compound metabolic process (GO:0034641)	3265	17	7.14	2.38	4.40E-02
								cellular macromolecule metabolic process (GO:0044260)	3864	19	8.45	2.25	4.31E-02
								organic substance metabolic process (GO:0071704)	5685	26	12.43	2.09	1.32E-02
								metabolic process (GO:0008152)	5907	26	12.91	2.01	1.81E-02
								nitrogen compound metabolic process (GO:0006807)	5018	22	10.97	2.01	4.13E-02
7	BANF1,CACYBP,CCT3,CCT5,CCT6A,CCT7,CHCHD2,COX5A,CYC1,ENO1,FABP5,GAPDH,HSP90AA1,HSPA4,HSPA8,MICOS10,MIF,MRPL37,MRPS15,MRPS34,NHP2,NME1,NME2,PA2G4,PDCD5,PHB,PKM,PRELID1,PTPN7,RAN,RANBP1,RPS26,SNRPB,SNRPD1,SNRPE,SNRPG,SQOR,SRA1,STIP1,STOML2,TCP1,TPI1	Glycolysis (P00024)	20	4	0.04	91.51	3.25E-05	spliceosomal snRNP assembly (GO:0000387)	15	3	0.03	91.51	8.58E-03
		De novo pyrimidine ribonucleotides biosythesis (P02740)	14	2	0.03	65.36	4.38E-02	ribonucleoside diphosphate metabolic process (GO:0009185)	22	3	0.05	62.39	1.20E-02
		De novo pyrimidine deoxyribonucleotide biosynthesis (P02739)	15	2	0.03	61	3.30E-02	purine ribonucleoside diphosphate metabolic process (GO:0009179)	22	3	0.05	62.39	9.57E-03
								purine nucleoside diphosphate metabolic process (GO:0009135)	22	3	0.05	62.39	7.98E-03
								ATP generation from ADP (GO:0006757)	22	3	0.05	62.39	6.84E-03
								glycolytic process (GO:0006096)	22	3	0.05	62.39	5.98E-03
								ADP metabolic process (GO:0046031)	22	3	0.05	62.39	5.32E-03
								nucleoside diphosphate phosphorylation (GO:0006165)	26	3	0.06	52.79	7.56E-03
								nucleotide phosphorylation (GO:0046939)	27	3	0.06	50.84	7.62E-03
								nucleoside diphosphate metabolic process (GO:0009132)	36	3	0.08	38.13	1.24E-02
								protein folding (GO:0006457)	107	8	0.23	34.21	2.93E-07
								carbohydrate catabolic process (GO:0016052)	45	3	0.1	30.5	1.74E-02
								ATP metabolic process (GO:0046034)	68	4	0.15	26.91	1.35E-02
								generation of precursor metabolites and energy (GO:0006091)	119	4	0.26	15.38	1.83E-02
								mRNA splicing, via spliceosome (GO:0000398)	176	5	0.38	13	8.36E-03
								RNA splicing, via transesterification reactions with bulged adenosine as	176	5	0.38	13	7.72E-03
								RNA splicing, via transesterification reactions (GO:0000375)	176	5	0.38	13	7.16E-03
								purine ribonucleotide metabolic process (GO:0009150)	152	4	0.33	12.04	3.89E-02
								ribonucleotide metabolic process (GO:0009259)	158	4	0.35	11.58	4.28E-02
								purine nucleotide metabolic process (GO:0006163)	161	4	0.35	11.37	4.39E-02
								ribose phosphate metabolic process (GO:0019693)	166	4	0.36	11.02	4.71E-02
								RNA splicing (GO:0008380)	221	5	0.48	10.35	1.79E-02
								ribonucleoprotein complex biogenesis (GO:0022613)	223	5	0.49	10.26	1.75E-02
								mRNA processing (GO:0006397)	229	5	0.5	9.99	1.77E-02
8	ARGLU1,BCLAF1,BRD2,BTG2,CCNL1,CD37,CD69,DDX3X,ELF1,FOS,GZMM,HCST,HLA-E,IER2,IFITM1,IFITM2,JAK1,JUN,JUNB,KLF6,LITAF,MALAT1,MCL1,NOSIP,PDE4D,PNISR,PPP1R15A,PRKCH,PRMT2,RBM39,RHOH,RSRP1,SF3B1,SLC2A3,SRGN,SRSF5,VAMP2,ZC3HAV1,ZFP36,ZFP36L1	Apoptosis signaling pathway (P00006)	125	4	0.25	16.07	1.98E-02	No significantly enriched GO-BP terms found					
		PDGF signaling pathway (P00047)	145	4	0.29	13.85	1.72E-02						
		CCKR signaling map (P06959)	173	4	0.34	11.61	2.21E-02						
		Angiogenesis (P00005)	181	4	0.36	11.1	1.96E-02						
9	ATP5F1B,ATP5MC3,C12orf75,CALR,CANX,CCND2,COTL1,DDOST,FKBP1A,FTL,GPI,HNRNPF,LDHA,MANF,NDUFAB1,P4HB,PDIA3,PDIA6,PFN1,PGAM1,PGD,PPA1,PPIA,PPIB,PRDX1,PRDX3,RPN1,RPN2,SDF2L1,SEC61B,SRPRA,SURF4,TXN,ZNF706	Pentose phosphate pathway (P02762)	9	2	0.01	> 100	2.30E-02	protein N-linked glycosylation via asparagine (GO:0018279)	8	2	0.01	> 100	4.36E-02
		Glycolysis (P00024)	20	2	0.03	60.56	4.78E-02	protein N-linked glycosylation (GO:0006487)	35	3	0.06	51.91	1.84E-02
								response to endoplasmic reticulum stress (GO:0034976)	90	5	0.15	33.64	1.04E-03
								protein folding (GO:0006457)	107	4	0.18	22.64	2.41E-02
								cellular response to stress (GO:0033554)	480	7	0.79	8.83	1.35E-02
								carbohydrate derivative metabolic process (GO:1901135)	437	6	0.72	8.31	3.40E-02
10	CHD4,DIAPH1,DOCK8,DYNC1H1,EIF3A,FLNA,GPRIN3,HUWE1,KTN1,MACF1,MT-ATP6,MT-ATP8,MT-CO1,MT-CO2,MT-CO3,MT-CYB,MT-ND1,MT-ND3,MT-ND4,MT-ND4L,MT-ND5,MT-ND6,MTRNR2L12,MYH9,RUNX3,SPTBN1,TFRC,TLN1,TNFRSF1B	No significantly enriched pathways found						proton transmembrane transport (GO:1902600)	60	5	0.09	57.19	7.92E-05
								aerobic respiration (GO:0009060)	57	4	0.08	48.16	1.99E-03
								cellular respiration (GO:0045333)	65	4	0.09	42.23	2.18E-03
								energy derivation by oxidation of organic compounds (GO:0015980)	81	4	0.12	33.89	3.77E-03
								generation of precursor metabolites and energy (GO:0006091)	119	4	0.17	23.07	1.30E-02
								inorganic cation transmembrane transport (GO:0098662)	310	5	0.45	11.07	2.66E-02
								inorganic ion transmembrane transport (GO:0098660)	334	5	0.49	10.27	3.29E-02
								cation transmembrane transport (GO:0098655)	343	5	0.5	10	3.31E-02
								cation transport (GO:0006812)	428	6	0.62	9.62	1.20E-02
								ion transport (GO:0006811)	606	6	0.88	6.8	4.79E-02
								ribosomal large subunit assembly (GO:0000027)	18	3	0.02	> 100	2.87E-04
								cytoplasmic translation (GO:0002181)	48	4	0.06	68.63	8.10E-05
								translational initiation (GO:0006413)	39	3	0.05	63.35	2.05E-03
								ribosomal large subunit biogenesis (GO:0042273)	52	4	0.06	63.35	1.01E-04
								ribosomal small subunit biogenesis (GO:0042274)	52	3	0.06	47.51	4.00E-03
								ribonucleoprotein complex assembly (GO:0022618)	95	5	0.12	43.35	2.65E-05
								ribonucleoprotein complex subunit organization (GO:0071826)	99	5	0.12	41.59	2.94E-05
								ribosome biogenesis (GO:0042254)	167	7	0.2	34.52	3.63E-07
								translational elongation (GO:0006414)	254	10	0.31	32.42	6.13E-10
								translation (GO:0006412)	254	10	0.31	32.42	3.07E-10

11	BTF3,COMMD6,EEF1A1,EEF2,EIF3E,EIF3F,EIF3L,HNRNPA1,RPL10,RPL15,RPL18,RPL22,RPL39,RPL5,RPL6,RPL7,RPLP1,RPS13,RPS14,RPS24,RPS28,RPS3A,RPS7,SLC25A6,SNHG29	No significantly enriched pathways found						peptide biosynthetic process (GO:0043043)	257	10	0.31	32.05	2.29E-10
								rRNA processing (GO:0006364)	110	4	0.13	29.95	1.34E-03
								rRNA metabolic process (GO:0016072)	117	4	0.14	28.16	1.60E-03
								amide biosynthetic process (GO:0043604)	295	10	0.36	27.92	6.47E-10
								non-membrane-bounded organelle assembly (GO:0140694)	150	5	0.18	27.45	1.68E-04
								peptide metabolic process (GO:0006518)	318	10	0.39	25.9	1.07E-09
								ribonucleoprotein complex biogenesis (GO:0022613)	223	7	0.27	25.85	1.96E-06
								cellular amide metabolic process (GO:0043603)	412	10	0.5	19.99	1.07E-08
								ncRNA processing (GO:0034470)	197	4	0.24	16.72	7.77E-03
								ncRNA metabolic process (GO:0034660)	251	4	0.3	13.12	1.64E-02
								organonitrogen compound biosynthetic process (GO:1901566)	692	10	0.84	11.9	1.12E-06
								organelle assembly (GO:0070925)	348	5	0.42	11.83	5.54E-03
								cellular protein-containing complex assembly (GO:0034622)	348	5	0.42	11.83	5.31E-03
								protein-containing complex assembly (GO:0065003)	367	5	0.45	11.22	6.53E-03
								protein-containing complex subunit organization (GO:0043933)	452	5	0.55	9.11	1.48E-02
								cellular component biogenesis (GO:0044085)	1024	7	1.24	5.63	1.32E-02
								cellular protein metabolic process (GO:0044267)	1765	10	2.14	4.67	2.37E-03
								gene expression (GO:0010467)	2894	14	3.51	3.98	1.70E-04
								protein metabolic process (GO:0019538)	2090	10	2.54	3.94	7.79E-03
								cellular macromolecule biosynthetic process (GO:0034645)	2326	10	2.82	3.54	1.66E-02
								macromolecule biosynthetic process (GO:0009059)	2332	10	2.83	3.53	1.64E-02
								cellular nitrogen compound biosynthetic process (GO:0044271)	2352	10	2.86	3.5	1.65E-02
								cellular nitrogen compound metabolic process (GO:0034641)	3265	13	3.96	3.28	3.35E-03
								organonitrogen compound metabolic process (GO:1901564)	2573	10	3.12	3.2	3.24E-02
								macromolecule metabolic process (GO:0043170)	4815	14	5.85	2.39	2.93E-02
12	CD3D,CD3G,CD52,HLA-B,HLA-C,IL2RG,ISG15,ISG20,LAT,LIMD2,LTB,MAL,PIM2,PSAP,RNF149,SHISA5,TPST2,UCP2,XBP1	T cell activation (P00053)	92	3	0.1	30.52	2.19E-02	No significantly enriched GO-BP terms found					

***Table S16**: Secreted cytokine level by FACS purified D14-REP $_{\gamma\delta}$ subsets*

		Cytokines (pg/ml)																		
**Sample**	**Subset**	**IL-1 beta**	**IL-4**	**IL-5**	**IL-6**	**IL-9**	**IL-10**	**IL-13**	**IL-17A (CTLA-8)**	**IL-18**	**IL-22**	**IL-12/IL-23p40**	**IL-27**	**IFN gamma**	**TNF alpha**	**MCP-1 (CCL2)**	**MIP-1 beta (CCL4)**	**CD62E (E-selectin)**	**Granzyme A**	**Granzyme B**
CB41365	$Vd1^+$	43.14	132.84	40.45	71.62	9	7204.97	52.35	40.63	50.09	71.68	4.37	427.04	10572.76	279.86	111.59	4927.2	1271.71	61286.81	128418995
CB41365	$Vd2^+$	43.14	135.59	35.52	76.96	9.2	8205.55	25.8	36.21	49.59	65.94	4.71	382.79	8374.31	247.34	117.63	4501.52	1256.68	61792.56	66561.69
CB41365	$Vd1^-2^-$	60.98	147.18	49.33	87.99	9.3	12397.13	16	36.98	51.23	68.86	8.1	431.21	10529.03	298.74	130.36	8230.95	1256.68	3219652.58	43243.11
CB45037	$Vd1^+$	56.37	152.76	45.49	54.57	9.23	16311.29	50.9	44.08	56.54	72.71	3.34	393.42	9028.89	309.15	106.06	6848.53	1304.12	3219652.58	41667.92
CB45037	$Vd2^+$	27.04	69.63	14.14	29.5	8.33	1200.53	12.63	19.99	37.53	47.81	1.63	253.87	1910.86	215.25	47.57	1023.84	1135.95	4761.81	2157.83
CB45037	$Vd1^-2^-$	29.83	114.94	21.59	116.05	7.93	3643.84	15.92	30.18	47.81	54.19	2.47	397.65	4098.74	251.35	89.99	3114.97	1157.79	26501.08	7411.29
CB45097	$Vd1^+$	51.73	159.37	34.3	60.64	9.1	11323.04	47.04	39.26	56.54	86.32	6.25	372.11	19400.85	337.96	109.45	6971.17	1316.52	67986.27	128418995
CB45097	$Vd2^+$	53.29	147.69	34.7	62.97	8.59	4173.82	46.31	38.75	64.81	70.23	9.15	480.61	7342.01	335.3	102.76	4637.37	1311.56	36545.53	128418995
CB45097	$Vd1^-2^-$	41.42	170.88	36.33	71.33	8.53	3698.9	33.63	39.51	52.26	69.36	6.99	378.53	9210.53	129.05	125.04	4308.19	1260.44	28489.08	128418995
CB45214	$Vd1^+$	34.77	95.63	30.09	50.69	8.46	3720.52	48.16	31.12	44.86	67	2.11	403.99	3963.25	259.51	77.66	4011.39	1206.23	14781.65	7969.12
CB45214	$Vd2^+$	40.27	111.74	32.49	60.73	8.93	7751.7	46.42	29.77	49.08	67.57	2.33	361.35	4323.9	325.76	86.46	5161.86	1173.14	15629.14	13603.13
CB45214	$Vd1^-2^-$	46.61	133.09	51.05	532.43	9.99	9404.76	59.59	38.75	54.02	77.22	2.45	387.05	6515.87	378.1	126.53	10706.69	1257.93	29156.41	33972.4

Table S17: GO enrichment analysis of up-regulated genes following K562-challenge using PANTHER GO-slim database

**Pathway**	**Gene list size**	**Observed**	**Expected**	**Enrichment**	**FDR**
intrinsic apoptotic signaling pathway in response to DNA damage (GO:0008630)	18	4	0.11	35.74	5.18E-03
T cell mediated immunity (GO:0002456)	22	4	0.14	29.25	6.92E-03
antigen processing and presentation (GO:0019882)	41	5	0.25	19.62	6.89E-03
intrinsic apoptotic signaling pathway (GO:0097193)	34	4	0.21	18.92	1.61E-02
regulation of adaptive immune response (GO:0002819)	36	4	0.22	17.87	1.70E-02
positive regulation of apoptotic process (GO:0043065)	50	5	0.31	16.09	7.17E-03
regulation of lymphocyte mediated immunity (GO:0002706)	41	4	0.25	15.69	2.10E-02
positive regulation of programmed cell death (GO:0043068)	52	5	0.32	15.47	7.48E-03
regulation of immune effector process (GO:0002697)	53	5	0.33	15.17	7.24E-03
peptidyl-tyrosine phosphorylation (GO:0018108)	44	4	0.27	14.62	2.56E-02
positive regulation of immune effector process (GO:0002699)	44	4	0.27	14.62	2.43E-02
positive regulation of cell death (GO:0010942)	55	5	0.34	14.62	6.99E-03
peptidyl-tyrosine modification (GO:0018212)	46	4	0.29	13.99	2.59E-02
regulation of leukocyte mediated immunity (GO:0002703)	47	4	0.29	13.69	2.68E-02
proton transmembrane transport (GO:1902600)	60	4	0.37	10.72	4.75E-02
ribonucleoprotein complex assembly (GO:0022618)	95	5	0.59	8.47	3.48E-02
ribonucleoprotein complex subunit organization (GO:0071826)	99	5	0.62	8.12	3.86E-02
regulation of cell death (GO:0010941)	188	8	1.17	6.84	6.70E-03
regulation of apoptotic process (GO:0042981)	166	7	1.03	6.78	1.73E-02
regulation of programmed cell death (GO:0043067)	171	7	1.06	6.58	1.68E-02
apoptotic process (GO:0006915)	245	10	1.52	6.57	9.44E-03
programmed cell death (GO:0012501)	253	10	1.57	6.36	6.20E-03
cell death (GO:0008219)	270	10	1.68	5.96	4.29E-03
actin cytoskeleton organization (GO:0030036)	294	9	1.83	4.92	1.68E-02
actin filament-based process (GO:0030029)	304	9	1.89	4.76	1.89E-02
cellular protein-containing complex assembly (GO:0034622)	348	9	2.16	4.16	3.53E-02
protein-containing complex assembly (GO:0065003)	367	9	2.28	3.94	4.25E-02
immune system process (GO:0002376)	694	13	4.31	3.01	3.76E-02
intracellular signal transduction (GO:0035556)	820	14	5.1	2.75	4.83E-02
cellular component assembly (GO:0022607)	896	15	5.57	2.69	4.03E-02
positive regulation of cellular process (GO:0048522)	1227	19	7.63	2.49	2.46E-02

Table S18: Distribution of total or T_{RM} precursor-like D15 $V_{\delta}1^{+}$, $V_{\delta}2^{+}$ & $V_{\delta}3^{+}$ cells across cell clusters

	Total D15 cells		
**D15-C**	**TRDV1**	**TRDV2**	**TRDV3**
**0**	12.9%	23.0%	27.2%
**1**	20.7%	17.2%	16.6%
**2**	17.6%	6.9%	23.1%
**3**	5.6%	15.1%	7.1%
**4**	12.0%	12.4%	7.7%
**5**	16.2%	6.5%	4.7%
**6**	8.1%	11.0%	8.3%
**7**	6.7%	7.9%	5.3%
**Total**	100.0%	100.0%	100.0%

	T_{RM} Precursor-like D15 cells		
**D15-C**	**TRDV1**	**TRDV2**	**TRDV3**
**0**	3.0%	4.2%	7.9%
**1**	7.5%	5.6%	15.8%
**2**	26.9%	16.9%	36.8%
**3**	25.4%	40.8%	21.1%
**4**	6.0%	9.9%	5.3%
**5**	9.0%	0.0%	2.6%
**6**	20.9%	22.5%	10.5%
**7**	1.5%	0.0%	0.0%
**Total**	100.0%	100.0%	100.0%

Table S19: Percentage of total or T_{RM} precursor-like $V_{\delta}1^{+}$, $V_{\delta}2^{+}$ & $V_{\delta}3^{+}$ cells in each D15 cell clusters

	Total D15 cells			
**D15-C**	**TRDV1**	**TRDV2**	**TRDV3**	**Total**
**0**	28.9%	42.1%	28.9%	100.0%
**1**	48.7%	32.9%	18.4%	100.0%
**2**	51.6%	16.4%	32.0%	100.0%
**3**	26.3%	57.9%	15.8%	100.0%
**4**	46.7%	39.1%	14.1%	100.0%
**5**	68.2%	22.4%	9.4%	100.0%
**6**	38.7%	42.7%	18.7%	100.0%
**7**	42.9%	41.1%	16.1%	100.0%

	T_{RM} Precursor-like D15 cells			
**D15-C**	**TRDV1**	**TRDV2**	**TRDV3**	**Total**
**0**	25.0%	37.5%	37.5%	100.0%
**1**	33.3%	26.7%	40.0%	100.0%
**2**	40.9%	27.3%	31.8%	100.0%
**3**	31.5%	53.7%	14.8%	100.0%
**4**	30.8%	53.8%	15.4%	100.0%
**5**	85.7%	0.0%	14.3%	100.0%
**6**	41.2%	47.1%	11.8%	100.0%
**7**	100.0%	0.0%	0.0%	100.0%

Table S20: Characteristics of primary AML patient samples used for experiments

Sample ID	FAB	Cytogenetics	Blast %
BM236	M5b	46,XX[20]	49
BM395	M5a	46,XY,add(2)(p11.2)[20]	98
BM454	M5b	48,XY,+8,inv(16)(p13.1q22),+22[20]	21
BM481	M5b	46,XY,del(11)(q23q24)[20]	21
BM845	M2	46,XX[19]*	80
BM1043	M4	46,XX,t(9;11)(p22;q23)[20]	53

** Structural rearrangement on 7q observed in 1 cell.*

Table S21: List of antibodies used for flow cytometry

Human Antigen	Fluorophore	Clone	Brand
CD3	APC	UCHT1	BD Pharmingen
CD3	PECy7	UCHT1	eBioscience
CD3	APC-Cy7	SK7	BD Pharmingen
CD15	APC or PE	HI98	BD Pharmingen
CD19	BV605	SJ25C1	BD Pharmingen
CD20	BV605	2H7	BD Pharmingen
CD25	APC	BC96	eBioscience
CD27	PerCP-eF710	O323	eBioscience
CD33	APC or PE	P67.6	BD Pharmingen
CD45	PE-Vio770	5B1	Miltenyi Biotec
CD45	BV785	HI30	BD Pharmingen
CD45RA	APC-H7	H100	BD Pharmingen
CD69	FITC	FN50	eBioscience
CD95	PECy7	DX2	eBioscience
CD107a	APC	H4A3	BioLegend
CCR5	APC	2D7	BD Pharmingen
CXCR3	APC-Cy7	G025H7	BioLegend
CXCR6	PE	K041E5	BioLegend
TCRγδ	PE	11F2	BD Pharmingen
Vδ1	FITC	TS8.2	Abcam
Vδ2	BV711	B6	BioLegend

Table S22: List of anti-human TotalSeq-C antibodies (all from Biolegend) for single cell immune profiling

Antigen	Clone	Barcode ID
CD107a (LAMP-1)	H4A3	155
CD152 (CTLA-4)	BNI3	151
CD183 (CXCR3)	G025H7	140
CD194 (CCR4)	L291H4	71
CD223 (LAG-3)	11C3C65	152
CD27	O323	154
CD279 (PD-1)	EH12.2H7	88
CD314 (NKG2D)	1D11	165
CD336 (NKp44)	P44-8	802
CD337 (NKp30)	P30-15	801
CD366 (Tim-3)	F38-2E2	169
CD56 (NCAM)	5.1H11	47
CD62L	DREG-56	147
CD95 (Fas)	DX2	156
TCR γδ	B1	139

Table S23: List of reference gene signatures used in this study

**Hallmark gene set from MSigDB**		**PMID: 34861191**	**PMID: 35113651**	**PMID: 35113651**
Glycolysis	Oxidative Phosphorylation	Exhaustion	TILneo4	TILneo8
ABCB6	ABCB7	HAVCR2	CXCL13	ATP10D
ADORA2B	ACAA1	RGS16	HMOX1	GZMB
AGL	ACAA2	SRGAP3	ETV7	ENTPD1
AGRN	ACADM	DUSP4	ADGRG1	KIR2DL4
AK3	ACADSB	CSF1	PDCD1	LAYN
AK4	ACADVL	TNFRSF18	ENTPD1	HTRA1
AKR1A1	ACAT1	NDFIP2	CCDC50	CD70
ALDH7A1	ACO2	SQLE	TOX	CXCR6
ALDH9A1	AFG3L2	ID3	CD4	HMOX1
ALDOA	AIFM1	SOX4	TIGIT	ADGRG1
ALDOB	ALAS1	CD9	TNFRSF18	LRRN3
ALG1	ALDH6A1	PHLDA1	NMB	ACP5
ANG	ATP1B1	CCL3	MYL6B	CTSW
ANGPTL4	ATP5F1A	CCL4	AHI1	GALNT2
ANKZF1	ATP5F1B	KLRC1	MAF	LINC01480
ARPP19	ATP5F1C	KLRD1	IFNG	CARS
ARTN	ATP5F1D	KLRB1	LAG3	LAG3
AURKA	ATP5F1E	CDK6	CXCR6	TOX
B3GALT6	ATP5MC1	PLS3	IGFLR1	PTPRCAP
B3GAT1	ATP5MC2	AFAP1L2	DUSP4	ASB2
B3GAT3	ATP5MC3	CTSW	ACP5	ITGB7
B3GNT3	ATP5ME	IL2RA	LINC01943	PTMS
B4GALT1	ATP5MF	AHI1	LIMS1	CD8A
B4GALT2	ATP5MG	RBPJ	BATF	GPR68
B4GALT4	ATP5PB	GZMB	PCED1B	NSMCE1
B4GALT7	ATP5PD	GNLY	ITGAL	ABI3
BIK	ATP5PF		YPEL2	SLC1A4
BPNT1	ATP5PO		MAL	PLEKHF1
CACNA1H	ATP6AP1		PPT1	CD8B
CAPN5	ATP6V0B		ELMO1	LINC01871
CASP6	ATP6V0C		MIS18BP1	CCL4
CD44	ATP6V0E1		TMEM173	NKG7
CDK1	ATP6V1C1		ADI1	CLIC3
CENPA	ATP6V1D		SLA	NDFIP2
CHPF	ATP6V1E1		GALM	PLPP1
CHPF2	ATP6V1F		LBH	PCED1B
CHST1	ATP6V1G1		SECISBP2L	CXCL13
CHST12	ATP6V1H		CTSB	PDCD1
CHST2	BAX		C17orf49	PRF1
CHST4	BCKDHA		CORO1B	HLA-DMA
CHST6	BDH2			GPR25
CITED2	CASP7			CD9
CLDN3	COX10			TIGIT
CLDN9	COX11			HLA-DRB5

CLN6	COX15			SYTL3
COG2	COX17			SLF1
COL5A1	COX4I1			NEK1
COPB2	COX5A			CASP1
CTH	COX5B			SMC4
CXCR4	COX6A1			TSEN54
CYB5A	COX6B1			PLSCR1
DCN	COX6C			GNPTAB
DDIT4	COX7A2			HLA-DPB1
DEPDC1	COX7A2L			PLEKHA1
DLD	COX7B			ARHGAP9
DPYSL4	COX7C			ALOX5AP
DSC2	COX8A			SH3BP1
ECD	CPT1A			NCF4
EFNA3	CS			NELL2
EGFR	CYB5A			GATA3
EGLN3	CYB5R3			PPM1M
ELF3	CYC1			TNFRSF1A
ENO1	CYCS			AC022706.1
ENO2	DECR1			MCM5
ERO1A	DLAT			HLA-DRB1
EXT1	DLD			TNFSF10
EXT2	DLST			TRIM21
FAM162A	ECH1			HDLBP
FBP2	ECHS1			ERN1
FKBP4	ECI1			CALHM2
FUT8	ETFA			SASH3
G6PD	ETFB			ACTA2
GAL3ST1	ETFDH			MAST4
GALE	FDX1			CAPG
GALK1	FH			MPST
GALK2	FXN			IGFLR1
GAPDHS	GLUD1			GZMA
GCLC	GOT2			CD27
GFPT1	GPI			ITGAE
GFUS	GPX4			SLA2
GLCE	GRPEL1			RHOC
GLRX	HADHA			COMMD8
GMPPA	HADHB			MYO1G
GMPPB	HCCS			SP140
GNE	HSD17B10			PHPT1
GNPDA1	HSPA9			CD2BP2
GOT1	HTRA2			PLEKHO1
GOT2	IDH1			STAM
GPC1	IDH2			MRPL16
GPC3	IDH3A			IL2RB
GPC4	IDH3B			ID2
GPR87	IDH3G			TESPA1
GUSB	IMMT			GOLGA8B

GYS1	ISCA1			MIS18BP1
GYS2	ISCU			VAMP5
HAX1	LDHA			DAPK2
HDLBP	LDHB			HLA-DPA1
HK2	LRPPRC			TSG101
HMMR	MAOB			IL4R
HOMER1	MDH1			CCND2
HS2ST1	MDH2			CTSC
HS6ST2	MFN2			TRAF3IP3
HSPA5	MGST3			NLRC3
IDH1	MPC1			ORAI3
IDUA	MRPL11			GNLY
IER3	MRPL15			MIR155HG
IGFBP3	MRPL34			CARD16
IL13RA1	MRPL35			CD82
IRS2	MRPS11			ECH1
ISG20	MRPS12			JAML
KDELR3	MRPS15			EEF1G
KIF20A	MRPS22			ETFB
KIF2A	MRPS30			DAXX
LCT	MTRF1			RBM4
LDHA	MTRR			HCST
LDHC	MTX2			RAB27A
LHPP	NDUFA1			YPEL2
LHX9	NDUFA2			CHST12
MDH1	NDUFA3			ARPC1B
MDH2	NDUFA4			PDIA4
ME1	NDUFA5			PDIA6
ME2	NDUFA6			AC243960.1
MED24	NDUFA7			TBC1D10C
MERTK	NDUFA8			PTPN6
MET	NDUFA9			PYCARD
MIF	NDUFAB1			BST2
MIOX	NDUFB1			BTN3A2
MPI	NDUFB2			MTG1
MXI1	NDUFB3			MLEC
NANP	NDUFB4			DUSP4
NASP	NDUFB5			GSDMD
NDST3	NDUFB6			SLAMF1
NDUFV3	NDUFB7			IFI6
NOL3	NDUFB8			PCID2
NSDHL	NDUFC1			GIMAP1
NT5E	NDUFC2			ITGA1
P4HA1	NDUFS1			CSNK2B
P4HA2	NDUFS2			CDK2AP2
PAM	NDUFS3			MYO1F
PAXIP1	NDUFS4			AC004687.1
PC	NDUFS6			PTTG1
PDK3	NDUFS7			APOBEC3C

PFKFB1	NDUFS8			TSPAN14
PFKP	NDUFV1			MOB3A
PGAM1	NDUFV2			STXBP2
PGAM2	NNT			LCP2
PGK1	NQO2			PLA2G16
PGLS	OAT			LINC00649
PGM2	OGDH			CST7
PHKA2	OPA1			TADA3
PKM	OXA1L			SIT1
PKP2	PDHA1			APOBEC3G
PLOD1	PDHB			SUSD3
PLOD2	PDHX			CD3G
PMM2	PDK4			CCL5
POLR3K	PDP1			CDC25B
PPFIA4	PHB2			TNFRSF1B
PPIA	PHYH			HMGN3
PPP2CB	PMPCA			THEMIS
PRPS1	POLR2F			ASF1A
PSMC4	POR			CTNNB1
PYGB	PRDX3			FIBP
PYGL	RETSAT			CCDC85B
QSOX1	RHOT1			POLR3GL
RARS1	RHOT2			GIMAP6
RBCK1	SDHA			ARL6IP1
RPE	SDHB			CALCOCO2
RRAGD	SDHC			CCPG1
SAP30	SDHD			KLRB1
SDC1	SLC25A11			ACAA2
SDC2	SLC25A12			ISG15
SDC3	SLC25A20			EIF4A1
SDHC	SLC25A3			CAT
SLC16A3	SLC25A4			MANF
SLC25A10	SLC25A5			XAB2
SLC25A13	SLC25A6			GRINA
SLC35A3	SUCLA2			GLO1
SLC37A4	SUCLG1			LSM2
SOD1	SUPV3L1			SLFN5
SOX9	SURF1			FKBP1A
SPAG4	TCIRG1			AKNA
SRD5A3	TIMM10			TAP1
STC1	TIMM13			LMO4
STC2	TIMM17A			APEH
STMN1	TIMM50			C12orf75
TALDO1	TIMM8B			TMEM14A
TFF3	TIMM9			DNPH1
TGFA	TOMM22			C17orf49
TGFBI	TOMM70			NUDT5
TKTL1	UQCR10			MGAT1
TPBG	UQCR11			CCDC69

TPI1	UQCRB			EIF4EBP1
TPST1	UQCRC1			PDHB
TXN	UQCRC2			ARL3
UGP2	UQCRFS1			UCP2
VCAN	UQCRH			IFI35
VEGFA	UQCRQ			HSBP1
VLDLR	VDAC1			LYST
XYLT2	VDAC2			MRFAP1L1
ZNF292	VDAC3			ITGAL
				AIP
				RASAL3
				CAPN1
				ITGB1
				RBPJ
				LBH
				DYNLL1
				NME2
				MT1F
				SYNGR2
				ABTB1
				ZGPAT
				CD63
				ILK
				SKA2
				TMEM204
				ACO2
				HOPX
				CRIP1
				OXNAD1
				CCS
				GRAP2
				GSTO1
				HADHB
				IL16
				PIN4
				CUEDC2
				CALM3
				SAMSN1
				HM13
				SNAP23
				LPCAT4
				FAAP20
				EFHD2
				PRDX3
				CCM2
				C22orf39
				SDHA
				ARRDC1
				MAP4K1

				NDUFA13
				IL27RA
				C14orf119

REFERENCES AND NOTES

1. S. Mensurado, R. Blanco-Domínguez, B. Silva-Santos, The emerging roles of γδ T cells in cancer immunotherapy. *Nat. Rev. Clin. Oncol.* **20**, 1–14 (2023).

2. L. S. Lamb Jr., P. J. Henslee-Downey, R. S. Parrish, K. Godder, J. Thompson, C. Lee, A. P. Gee, Increased frequency of TCR gamma delta + T cells in disease-free survivors following T cell-depleted, partially mismatched, related donor bone marrow transplantation for leukemia. *J. Hematother.* **5**, 503–509 (1996).

3. K. T. Godder, P. J. Henslee-Downey, J. Mehta, B. S. Park, K. Y. Chiang, S. Abhyankar, L. S. Lamb, Long term disease-free survival in acute leukemia patients recovering with increased γδ T cells after partially mismatched related donor bone marrow transplantation. *Bone Marrow Transplant.* **39**, 751–757 (2007).

4. A. J. Gentles, A. M. Newman, C. L. Liu, S. V. Bratman, W. Feng, D. Kim, V. S. Nair, Y. Xu, A. Khuong, C. D. Hoang, M. Diehn, R. B. West, S. K. Plevritis, A. A. Alizadeh, The prognostic landscape of genes and infiltrating immune cells across human cancers. *Nat. Med.* **21**, 938–945 (2015).

5. E. Foord, L. C. M. Arruda, A. Gaballa, C. Klynning, M. Uhlin, Characterization of ascites- and tumor-infiltrating γδ T cells reveals distinct repertoires and a beneficial role in ovarian cancer. *Sci. Transl. Med.* **13**, eabb0192 (2021).

6. Y. Wu, D. Biswas, I. Usaite, M. Angelova, S. Boeing, T. Karasaki, S. Veeriah, J. Czyzewska-Khan, C. Morton, M. Joseph, S. Hessey, J. Reading, A. Georgiou, M. al-Bakir; TRACERx Consortium, N. J. Birkbak, G. Price, M. Khalil, K. Kerr, S. Richardson, H. Cheyne, T. Cruickshank, G. A. Wilson, R. Rosenthal, H. Aerts, M. Hewish, G. Anand, S. Khan, K. Lau, M. Sheaff, P. Schmid, L. Lim, J. Conibear, R. Schwarz, T. L. Kaufmann, M. Huska, J. Shaw, J. Riley, L. Primrose, D. Fennell, A. Hackshaw, Y. Ngai, A. Sharp, O. Pressey, S. Smith, N. Gower, H. K. Dhanda, K. Chan, S. Chakraborty, K. Litchfield, K. Thakkar, J. Tugwood, A. Clipson, C. Dive, D. Rothwell, A. Kerr, E. Kilgour, F. Morgan, M. Kornaszewska, R. Attanoos, H. Davies, K. Baker, M. Carter, C. R. Lindsay, F. Gomes, F. Blackhall, L. Priest,

M. G. Krebs, A. Chaturvedi, P. Oliveira, Z. Szallasi, G. Royle, C. Veiga, M. Skrzypski, R. Salgado, M. Diossy, A. Kirk, M. Asif, J. Butler, R. Bilancia, N. Kostoulas, M. Thomas, M. MacKenzie, M. Wilcox, A. Nakas, S. Rathinam, R. Boyles, M. Tufail, A. Bajaj, K. Ang, M. F. Chowdhry, M. Shackcloth, J. Asante-Siaw, A. Leek, N. Totten, J. D. Hodgkinson, P. van Loo, W. Monteiro, H. Marshal, K. G. Blyth, C. Dick, C. Fekete, E. Lim, P. de Sousa, S. Jordan, A. Rice, H. Raubenheimer, H. Bhayani, M. Hamilton, L. Ambrose, A. Devaraj, H. Chavan, S. Begum, S. I. Buderi, D. Kaniu, M. Malima, S. Booth, A. G. Nicholson, N. Fernandes, P. Shah, C. Proli, J. Gosney, S. Danson, J. Bury, J. Edwards, J. Hill, S. Matthews, Y. Kitsanta, J. Rao, S. Tenconi, L. Socci, K. Suvarna, F. Kibutu, P. Fisher, R. Young, J. Barker, F. Taylor, K. Lloyd, J. Lester, M. Escudero, A. Stewart, A. Rowan, J. Goldman, R. K. Stone, T. Denner, E. Nye, M. Greco, J. Nicod, C. Puttick, K. Enfield, E. Colliver, A. Magness, C. Bailey, K. Dijkstra, V. Barbè, R. Vendramin, J. Kisistok, M. Sokac, J. Demeulemeester, E. L. Cadieux, C. Castignani, H. Fu, K. Grigoriadis, C. Lee, F. Athanasopoulou, C. Hiley, L. Robinson, T. Horey, P. Russell, D. Papadatos-Pastos, S. Lock, K. Gilbert, K. Selvaraju, P. Ashford, O. Pich, T. B. K. Watkins, S. Ward, E. Lim, A. M. Frankell, C. Abbosh, R. E. Hynds, M. W. Sunderland, K. Peggs, T. Marafioti, J. A. Hartley, H. Lowe, L. Ensell, V. Spanswick, A. Karamani, D. Moore, S. Beck, O. Chervova, M. Tanic, A. Huebner, M. Dietzen, J. R. M. Black, C. M. Ruiz, R. Bentham, C. Naceur-Lombardelli, H. Zhai, N. Kanu, F. Gimeno-Valiente, S. K. Bola, I. G. Matos, M. Shah, F. G. Cancino, D. Karagianni, M. Razaq, M. Akther, D. Johnson, J. Laycock, E. Hoxha, B. Chain, D. R. Pearce, K. Chen, J. Herrero, F. Monk, S. Zaccaria, N. Magno, P. Prymas, A. Toncheva, M. Sivakumar, O. Lucas, M. S. Hill, O. al-Sawaf, S. K. Ung, S. Gamble, S. Wong, D. Lawrence, M. Hayward, N. Panagiotopoulos, R. George, D. Patrini, M. Falzon, E. Borg, R. Khiroya, A. Ahmed, M. Taylor, J. Choudhary, S. M. Janes, M. Forster, T. Ahmad, S. M. Lee, N. Navani, M. Scarci, P. Gorman, E. Bertoja, R. C. M. Stephens, E. M. Hoogenboom, J. W. Holding, S. Bandula, R. Thakrar, J. Wilson, M. Shah, Marcos, V. Duran, M. Litovchenko, S. Vanloo, P. Pawlik, K. Thol, B. Naidu, G. Langman, H. Bancroft, S. Kadiri, G. Middleton, M. Djearaman, A. Osman, H. Shackleford, A. Patel, C. Ottensmeier, S. Chee, A. Alzetani, J. Cave, L. Scarlett, J. Richards, P. Ingram, E. Shaw, J. le Quesne, A. Dawson, D. Marrone, S. Dulloo, C. Wilson, Y. Summers, R. Califano, R. Shah, P. Krysiak, K. Rammohan, E. Fontaine, R. Booton, M. Evison, S. Moss, J. Novasio, L. Joseph, P. Bishop, H. Doran, F.

Granato, V. Joshi, E. Smith, A. Montero, P. Crosbie, N. McGranahan, M. Jamal-Hanjani, A. Hackshaw, S. A. Quezada, A. C. Hayday, C. Swanton, A local human vδ1 T cell population is associated with survival in non small-cell lung cancer. *Nat. Cancer* **3**, 696–709 (2022).

7. A. Choudhary, F. Davodeau, A. Moreau, M. A. Peyrat, M. Bonneville, F. Jotereau, Selective lysis of autologous tumor cells by recurrent gamma delta tumor-infiltrating lymphocytes from renal carcinoma. *J. Immunol.* **154**, 3932–3940 (1995).

8. E. Viey, G. Fromont, B. Escudier, Y. Morel, S. da Rocha, S. Chouaib, A. Caignard, Phosphostim-activated γδ T cells kill autologous metastatic renal cell carcinoma. *J. Immunol.* **174**, 1338–1347 (2005).

9. M. Rigau, S. Ostrouska, T. S. Fulford, D. N. Johnson, K. Woods, Z. Ruan, H. E. G. McWilliam, C. Hudson, C. Tutuka, A. K. Wheatley, S. J. Kent, J. A. Villadangos, B. Pal, C. Kurts, J. Simmonds, M. Pelzing, A. D. Nash, A. Hammet, A. M. Verhagen, G. Vairo, E. Maraskovsky, C. Panousis, N. A. Gherardin, J. Cebon, D. I. Godfrey, A. Behren, A. P. Uldrich, Butyrophilin 2A1 is essential for phosphoantigen reactivity by γδ T cells. *Science* **367**, eaay5516 (2020).

10. M. M. Karunakaran, C. R. Willcox, M. Salim, D. Paletta, A. S. Fichtner, A. Noll, L. Starick, A. Nöhren, C. R. Begley, K. A. Berwick, R. A. G. Chaleil, V. Pitard, J. Déchanet-Merville, P. A. Bates, B. Kimmel, T. J. Knowles, V. Kunzmann, L. Walter, M. Jeeves, F. Mohammed, B. E. Willcox, T. Herrmann, Butyrophilin-2A1 directly binds germline-encoded regions of the vγ9vδ2 TCR and is essential for phosphoantigen sensing. *Immunity* **52**, 487–498.e6 (2020).

11. S. Vavassori, A. Kumar, G. S. Wan, G. S. Ramanjaneyulu, M. Cavallari, S. el Daker, T. Beddoe, A. Theodossis, N. K. Williams, E. Gostick, D. A. Price, D. U. Soudamini, K. K. Voon, M. Olivo, J. Rossjohn, L. Mori, G. de Libero, Butyrophilin 3A1 binds phosphorylated antigens and stimulates human γδ T cells. *Nat. Immunol.* **14**, 908–916 (2013).

12. A. Sandstrom, C. M. Peigné, A. Léger, J. E. Crooks, F. Konczak, M. C. Gesnel, R. Breathnach, M. Bonneville, E. Scotet, E. J. Adams, The intracellular B30.2 domain of

butyrophilin 3A1 binds phosphoantigens to mediate activation of human vγ9vδ2 t cells. *Immunity* **40**, 490–500 (2014).

13. Z. Sebestyen, I. Prinz, J. Déchanet-Merville, B. Silva-Santos, J. Kuball, Translating gammadelta (γδ) T cells and their receptors into cancer cell therapies. *Nat. Rev. Drug Discov.* **19**, 169–184 (2020).

14. P. L. Ryan, N. Sumaria, C. J. Holland, C. M. Bradford, N. Izotova, C. L. Grandjean, A. S. Jawad, L. A. Bergmeier, D. J. Pennington, Heterogeneous yet stable Vδ2$^+$ T-cell profiles define distinct cytotoxic effector potentials in healthy human individuals. *Proc. Natl. Acad. Sci. U.S.A.* **113**, 14378–14383 (2016).

15. K. M. Wragg, H. X. Tan, A. B. Kristensen, C. V. Nguyen-Robertson, A. D. Kelleher, M. S. Parsons, A. K. Wheatley, S. P. Berzins, D. G. Pellicci, S. J. Kent, J. A. Juno, High CD26 and low CD94 expression identifies an IL-23 responsive Vδ2$^+$ T cell subset with a MAIT cell-like transcriptional profile. *Cell Rep.* **31**, 107773 (2020).

16. M. S. Davey, C. R. Willcox, S. P. Joyce, K. Ladell, S. A. Kasatskaya, J. E. McLaren, S. Hunter, M. Salim, F. Mohammed, D. A. Price, D. M. Chudakov, B. E. Willcox, Clonal selection in the human Vδ1 T cell repertoire indicates γδ TCR-dependent adaptive immune surveillance. *Nat. Commun.* **8**, 14760 (2017).

17. S. Ravens, C. Schultze-Florey, S. Raha, I. Sandrock, M. Drenker, L. Oberdörfer, A. Reinhardt, I. Ravens, M. Beck, R. Geffers, C. von Kaisenberg, M. Heuser, F. Thol, A. Ganser, R. Förster, C. Koenecke, I. Prinz, Human γδ T cells are quickly reconstituted after stem-cell transplantation and show adaptive clonal expansion in response to viral infection. *Nat. Immunol.* **18**, 393–401 (2017).

18. T. Dimova, M. Brouwer, F. Gosselin, J. Tassignon, O. Leo, C. Donner, A. Marchant, D. Vermijlen, Effector Vγ9Vδ2 T cells dominate the human fetal γδ T-cell repertoire. *Proc. Natl. Acad. Sci. U.S.A.* **112**, e556–e565 (2015).

19. L. Tan, A. S. Fichtner, E. Bruni, I. Odak, I. Sandrock, A. Bubke, A. Borchers, C. Schultze-Florey, C. Koenecke, R. Förster, M. Jarek, C. von Kaisenberg, A. Schulz, X. Chu, B. Zhang, Y. Li, U. Panzer, C. F. Krebs, S. Ravens, I. Prinz, A fetal wave of human type 3 effector γδ cells with restricted TCR diversity persists into adulthood. *Sci. Immunol.* **6**, eabf0125 (2021).

20. J. P. H. Fisher, M. Yan, J. Heuijerjans, L. Carter, A. Abolhassani, J. Frosch, R. Wallace, B. Flutter, A. Capsomidis, M. Hubank, N. Klein, R. Callard, K. Gustafsson, J. Anderson, Neuroblastoma killing properties of Vδ2 and Vδ2-negative γδ T cells following expansion by artificial antigen-presenting cells. *Clin. Cancer Res.* **20**, 5720–5732 (2014).

21. D. C. Deniger, S. N. Maiti, T. Mi, K. C. Switzer, V. Ramachandran, L. V. Hurton, S. Ang, S. Olivares, B. A. Rabinovich, M. H. Huls, D. A. Lee, R. C. Bast Jr., R. E. Champlin, L. J. N. Cooper, Activating and propagating polyclonal gamma delta T cells with broad specificity for malignancies. *Clin. Cancer Res.* **20**, 5708–5719 (2014).

22. A. R. Almeida, D. V. Correia, A. Fernandes-Platzgummer, C. L. da Silva, M. G. da Silva, D. R. Anjos, B. Silva-Santos, Delta one T cells for immunotherapy of chronic lymphocytic leukemia: Clinical-grade expansion/differentiation and preclinical proof of concept. *Clin. Cancer Res.* **22**, 5795–5804 (2016).

23. G. M. Siegers, H. Dhamko, X.-H. Wang, A. M. Mathieson, Y. Kosaka, T. C. Felizardo, J. A. Medin, S. Tohda, J. Schueler, P. Fisch, A. Keating, Human Vδ1 γδ T cells expanded from peripheral blood exhibit specific cytotoxicity against B-cell chronic lymphocytic leukemia-derived cells. *Cytotherapy* **13**, 753–764 (2011).

24. D. V. Correia, M. Fogli, K. Hudspeth, M. G. da Silva, D. Mavilio, B. Silva-Santos, Differentiation of human peripheral blood Vδ 1^+ T cells expressing the natural cytotoxicity receptor NKP30 for recognition of lymphoid leukemia cells. *Blood* **118**, 992–1001 (2011).

25. J. L. Nours, N. A. Gherardin, S. H. Ramarathinam, W. Awad, F. Wiede, B. S. Gully, Y. Khandokar, T. Praveena, J. M. Wubben, J. J. Sandow, A. I. Webb, A. von Borstel, M. T. Rice, S. J. Redmond, R. Seneviratna, M. L. Sandoval-Romero, S. Li, M. N. T. Souter, S. B. G. Eckle, A. J. Corbett, H. H. Reid, L. Liu, D. P. Fairlie, E. M. Giles, G. P. Westall, R. W.

Tothill, M. S. Davey, R. Berry, T. Tiganis, J. M. Cluskey, D. G. Pellicci, A. W. Purcell, A. P. Uldrich, D. I. Godfrey, J. Rossjohn, A class of γδ T cell receptors recognize the underside of the antigen-presenting molecule MR1. *Science* **366**, 1522–1527 (2019).

26. A. M. Luoma, C. D. Castro, T. Mayassi, L. A. Bembinster, L. Bai, D. Picard, B. Anderson, L. Scharf, J. E. Kung, L. V. Sibener, P. B. Savage, B. Jabri, A. Bendelac, E. J. Adams, Crystal structure of Vδ1 T cell receptor in complex with CD1D-sulfatide shows MHC-like recognition of a self-lipid by human γδ T cells. *Immunity* **39**, 1032–1042 (2013).

27. J. P. H. Fisher, M. Yan, J. Heuijerjans, L. Carter, A. Abolhassani, J. Frosch, R. Wallace, B. Flutter, A. Capsomidis, M. Hubank, N. Klein, R. Callard, K. Gustafsson, J. Anderson, A simple and robust single-step method for CAR-Vδ1 γδ T cell expansion and transduction for cancer immunotherapy. *Front. Immunol.* **13**, 863155 (2022).

28. S. C. De Rosa, J. P. Andrus, S. P. Perfetto, J. J. Mantovani, L. A. Herzenberg, L. A. Herzenberg, M. Roederer, Ontogeny of γδ T cells in humans. *J. Immunol.* **172**, 1637–1645 (2004).

29. M. Papadopoulou, T. Dimova, M. Shey, L. Briel, H. Veldtsman, N. Khomba, H. Africa, M. Steyn, W. A. Hanekom, T. J. Scriba, E. Nemes, D. Vermijlen, Fetal public Vγ9Vδ2 T cells expand and gain potent cytotoxic functions early after birth. *Proc. Natl. Acad. Sci.* **117**, 18638–18648 (2020).

30. S. R. Riddell, K. S. Watanabe, J. M. Goodrich, C. R. Li, M. E. Agha, P. D. Greenberg, Restoration of viral immunity in immunodeficient humans by the adoptive transfer of T cell clones. *Science* **257**, 238–41 (1992).

31. M. E. Dudley, L. T. Ngo, J. Westwood, J. R. Wunderlich, S. A. Rosenberg, T-cell clones from melanoma patients immunized against an anchor-modified gp100 peptide display discordant effector phenotypes. *Cancer J.* **6**, 69–77 (2000).

32. M. S. Davey, C. R. Willcox, S. Hunter, S. A. Kasatskaya, E. B. M. Remmerswaal, M. Salim, F. Mohammed, F. J. Bemelman, D. M. Chudakov, Y. H. Oo, B. E. Willcox, The human Vδ2$^+$

T-cell compartment comprises distinct innate-like Vγ9$^+$ and adaptive Vγ9$^-$ subsets. *Nat. Commun.* **9**, 1760 (2018).

33. M. S. Davey, C. R. Willcox, A. T. Baker, S. Hunter, B. E. Willcox, Recasting human Vδ1 lymphocytes in an adaptive role. *Trends Immunol.* **39**, 446–459 (2018).

34. M. Gutierrez-Arcelus, N. Teslovich, A. R. Mola, R. B. Polidoro, A. Nathan, H. Kim, S. Hannes, K. Slowikowski, G. F. M. Watts, I. Korsunsky, M. B. Brenner, S. Raychaudhuri, P. J. Brennan, Lymphocyte innateness defined by transcriptional states reflects a balance between proliferation and effector functions. *Nat. Commun.* **10**, 687 (2019).

35. F. Davodeau, M. A. Peyrat, M. M. Hallet, J. Gaschet, I. Houde, R. Vivien, H. Vie, M. Bonneville, Close correlation between Daudi and mycobacterial antigen recognition by human gamma delta T cells and expression of V9JPC1 gamma/V2DJC delta-encoded T cell receptors. *J. Immunol.* **151**, 1214–1223 (1993).

36. P. S. Evans, P. J. Enders, C. Yin, T. J. Ruckwardt, M. Malkovsky, C. D. Pauza, In vitro stimulation with a non-peptidic alkylphosphate expands cells expressing Vgamma2-jgamma1.2/Vdelta2 T-cell receptors. *Immunology* **104**, 19–27 (2001).

37. S. Krishna, F. J. Lowery, A. R. Copeland, E. Bahadiroglu, R. Mukherjee, L. Jia, J. T. Anibal, A. Sachs, S. O. Adebola, D. Gurusamy, Z. Yu, V. Hill, J. J. Gartner, Y. F. Li, M. Parkhurst, B. Paria, P. Kvistborg, M. C. Kelly, S. L. Goff, G. Altan-Bonnet, P. F. Robbins, S. A. Rosenberg, Stem-like CD8 T cells mediate response of adoptive cell immunotherapy against human cancer. *Science* **370**, 1328–1334 (2020).

38. L. Gattinoni, E. Lugli, Y. Ji, Z. Pos, C. M. Paulos, M. F. Quigley, J. R. Almeida, E. Gostick, Z. Yu, C. Carpenito, E. Wang, D. C. Douek, D. A. Price, C. H. June, F. M. Marincola, M. Roederer, N. P. Restifo, A human memory T cell subset with stem cell–Like properties. *Nat. Med.* **17**, 1290–1297 (2011).

39. J. A. Shyer, R. A. Flavell, W. Bailis. Metabolic signaling in T cells. *Cell Res.* **30**, 649–659 (2020).

40. J. J. Melenhorst, G. M. Chen, M. Wang, D. L. Porter, C. Chen, M. K. A. Collins, P. Gao, S. Bandyopadhyay, H. Sun, Z. Zhao, S. Lundh, I. Pruteanu-Malinici, C. L. Nobles, S. Maji, N. V. Frey, S. I. Gill, A. W. Loren, L. Tian, I. Kulikovskaya, M. Gupta, D. E. Ambrose, M. M. Davis, J. A. Fraietta, J. L. Brogdon, R. M. Young, A. Chew, B. L. Levine, D. L. Siegel, C. Alanio, E. J. Wherry, F. D. Bushman, S. F. Lacey, K. Tan, C. H. June, Decade-long leukaemia remissions with persistence of $CD4^+$ CAR T cells. *Nature* **602**, 503–509 (2022).

41. F. J. Lowery, S. Krishna, R. Yossef, N. B. Parikh, P. D. Chatani, N. Zacharakis, M. R. Parkhurst, N. Levin, S. Sindiri, A. Sachs, K. J. Hitscherich, Z. Yu, N. R. Vale, Y. C. Lu, Z. Zheng, L. Jia, J. J. Gartner, V. K. Hill, A. R. Copeland, S. K. Nah, R. V. Masi, B. Gasmi, S. Kivitz, B. C. Paria, M. Florentin, S. P. Kim, K. I. Hanada, Y. F. Li, L. T. Ngo, S. Ray, M. L. Shindorf, S. T. Levi, R. Shepherd, C. Toy, A. Y. Parikh, T. D. Prickett, M. C. Kelly, R. Beyer, S. L. Goff, J. C. Yang, P. F. Robbins, S. A. Rosenberg, Molecular signatures of antitumor neoantigen-reactive T cells from metastatic human cancers. *Science* **375**, 877–884 (2022).

42. C. R. Good, M. A. Aznar, S. Kuramitsu, P. Samareh, S. Agarwal, G. Donahue, K. Ishiyama, N. Wellhausen, A. K. Rennels, Y. Ma, L. Tian, S. Guedan, K. A. Alexander, Z. Zhang, P. C. Rommel, N. Singh, K. M. Glastad, M. W. Richardson, K. Watanabe, J. L. Tanyi, M. H. O'Hara, M. Ruella, S. F. Lacey, E. K. Moon, S. J. Schuster, S. M. Albelda, L. L. Lanier, R. M. Young, S. L. Berger, C. H. June, An NK-like CAR T cell transition in CAR T cell dysfunction. *Cell* **184**, 6081–6100.e26 (2021).

43. H. Li, A. M. van der Leun, I. Yofe, Y. Lubling, D. Gelbard-Solodkin, A. C. J. van Akkooi, M. van den Braber, E. A. Rozeman, J. B. A. G. Haanen, C. U. Blank, H. M. Horlings, E. David, Y. Baran, A. Bercovich, A. Lifshitz, T. N. Schumacher, A. Tanay, I. Amit, Dysfunctional CD8 T cells form a proliferative, dynamically regulated compartment within human melanoma. *Cell* **176**, 775–789.e18 (2019).

44. K. Hochheiser, F. Wiede, T. Wagner, D. Freestone, M. H. Enders, M. Olshansky, B. Russ, S. Nüssing, E. Bawden, A. Braun, A. Bachem, E. Gressier, R. McConville, S. L. Park, C. M. Jones, G. M. Davey, D. E. Gyorki, D. Tscharke, I. A. Parish, S. Turner, M. J. Herold, T.

Tiganis, S. Bedoui, T. Gebhardt, Ptpn2 and Klrg1 regulate the generation and function of tissue-resident memory CD8$^+$ T cells in skin. *J. Exp. Med.* **218**, e20200940 (2021).

45. L. Parga-Vidal, F. M. Behr, N. A. M. Kragten, B. Nota, T. H. Wesselink, I. Kavazović, L. E. Covill, M. B. P. Schuller, Y. T. Bryceson, F. M. Wensveen, R. A. W. van Lier, T. J. P. van Dam, R. Stark, K. P. J. M. van Gisbergen, Hobit identifies tissue-resident memory T cell precursors that are regulated by Eomes. *Sci. Immunol.* **6**, eabg3533 (2021).

46. G. Pizzolato, H. Kaminski, M. Tosolini, D. M. Franchini, F. Pont, F. Martins, C. Valle, D. Labourdette, S. Cadot, A. Quillet-Mary, M. Poupot, C. Laurent, L. Ysebaert, S. Meraviglia, F. Dieli, P. Merville, P. Milpied, J. Déchanet-Merville, J. J. Fournié, Single-cell RNA sequencing unveils the shared and the distinct cytotoxic hallmarks of human TCRVδ1 and TCRVδ2 γδ T lymphocytes. *Proc. Natl. Acad. Sci. U.S.A.* **116**, 11906–15 (2019).

47. D. Wu, P. Wu, X. Wu, J. Ye, Z. Wang, S. Zhao, C. Ni, G. Hu, J. Xu, Y. Han, T. Zhang, F. Qiu, J. Yan, J. Huang, Ex vivo expanded human circulating Vδ1 γδT cells exhibit favorable therapeutic potential for colon cancer. *Onco. Targets. Ther.* **4**, e992749 (2015).

48. J. Saura-Esteller, M. de Jong, L. A. King, E. Ensing, B. Winograd, T. D. de Gruijl, P. W. H. I. Parren, H. J. van der Vliet, Gamma delta T-cell based cancer immunotherapy: Past-present-future. *Front. Immunol.* **13**, 915837 (2022).

49. L. Gattinoni, Acquisition of full effector function in vitro paradoxically impairs the in vivo antitumor efficacy of adoptively transferred CD8$^+$ T cells. *J. Clin. Investig.* **115**, 1616–1626 (2005).

50. C. S. Hinrichs, Z. A. Borman, L. Cassard, L. Gattinoni, R. Spolski, Z. Yu, L. Sanchez-Perez, P. Muranski, S. J. Kern, C. Logun, D. C. Palmer, Y. Ji, R. N. Reger, W. J. Leonard, R. L. Danner, S. A. Rosenberg, N. P. Restifo, Adoptively transferred effector cells derived from naïve rather than central memory CD8$^+$ T cells mediate superior antitumor immunity. *Proc. Natl. Acad. Sci. U.S.A.* **106**, 17469–17474 (2009).

51. S. Ravens, A. S. Fichtner, M. Willers, D. Torkornoo, S. Pirr, J. Schöning, M. Deseke, I. Sandrock, A. Bubke, A. Wilharm, D. Dodoo, B. Egyir, K. L. Flanagan, L. Steinbrück, P. Dickinson, P. Ghazal, B. Adu, D. Viemann, I. Prinz, Microbial exposure drives polyclonal expansion of innate γδ T cells immediately after birth. *Proc. Natl. Acad. Sci. U.S.A.* **117**, 18649–18660 (2020).

52. G. Sanchez Sanchez, M. Papadopoulou, A. Azouz, Y. Tafesse, A. Mishra, J. K. Y. Chan, Y. Fan, I. Verdebout, S. Porco, F. Libert, F. Ginhoux, B. Vandekerckhove, S. Goriely, D. Vermijlen, Identification of distinct functional thymic programming of fetal and pediatric human γδ thymocytes via single-cell analysis. *Nat. Commun.* **13**, 5842 (2022).

53. J. Lieberman. Granzyme A activates another way to die. *Immunol. Rev.* **235**, 93–104 (2010).

54. K. R. van Daalen, J. F. Reijneveld, N. Bovenschen. Modulation of inflammation by extracellular granzyme A. *Front. Immunol.* **11**, 931 (2020).

55. J. C. Ribot, N. Lopes, B. Silva-Santos. γδ T cells in tissue physiology and surveillance. *Nat. Rev. Immunol.* **21**, 221–232 (2021).

56. M. Brandes, K. Willimann, B. Moser. Professional antigen-presentation function by human gammadelta T cells. *Science* **309**, 264–268 (2005).

57. M. Brandes, K. Willimann, G. Bioley, N. Lévy, M. Eberl, M. Luo, R. Tampé, F. Lévy, P. Romero, B. Moser. Cross-presenting human γδ T cells induce robust $CD8^+$ αβ T cell responses. *Proc. Natl. Acad. Sci. U.S.A.* **106**, 2307–2312 (2009).

58. T Metsalu, J Vilo. ClustVis: A web tool for visualizing clustering of multivariate data using principal component analysis and heatmap. *Nucleic Acids Res.* **43**, W566–W570 (2015).

59. E. P Mimitou, A. Cheng, A. Montalbano, S. Hao, M. Stoeckius, M. Legut, T. Roush, A. Herrera, E. Papalexi, Z. Ouyang, R. Satija, N. E. Sanjana, S. B. Koralov, P. Smibert, Multiplexed detection of proteins, transcriptomes, clonotypes and CRISPR perturbations in single cells. *Nat. Methods* **16**, 409–412 (2019).

60. Y. Hao, S. Hao, E. Andersen-Nissen, W. M. Mauck III, S. Zheng, A. Butler, M. J. Lee, A. J. Wilk, C. Darby, M. Zager, P. Hoffman, M. Stoeckius, E. Papalexi, E. P. Mimitou, J. Jain, A. Srivastava, T. Stuart, L. M. Fleming, B. Yeung, A. J. Rogers, J. M. McElrath, C. A. Blish, R. Gottardo, P. Smibert, R. Satija, Integrated analysis of multimodal single-cell data. *Cell* **184**, 3573–3587.e29 (2021).

61. X. Qiu, Q. Mao, Y. Tang, L. Wang, R. Chawla, H A. Pliner, C. Trapnell, Reversed graph embedding resolves complex single-cell trajectories. *Nat. Methods* **14**, 979–982 (2017).

62. K. Street, D. Risso, R. B. Fletcher, D. Das, J. Ngai, N. Yosef, E. Purdom, S. Dudoit. Slingshot: Cell lineage and pseudotime inference for single-cell transcriptomics. *BMC Genomics* **19**, 477 (2018).
